# Efficient dispersal and substrate acquisition traits in a marine invasive species via transient chimerism and colony mobility

**DOI:** 10.7717/peerj.5006

**Published:** 2018-06-13

**Authors:** Andrew E. Fidler, Aurelie Bacq-Labreuil, Elad Rachmilovitz, Baruch Rinkevich

**Affiliations:** 1 Institute of Marine Science, University of Auckland, Auckland, New Zealand; 2 École Supérieure de Biotechnologie Strasbourg, Strasbourg, France; 3 National Institute of Oceanography, Haifa, Israel

**Keywords:** Allorecognition, Invasive species, Biofouling, Immunity, Substrate acquisition, Bioinvasion

## Abstract

Over the past three decades the colonial ascidian *Didemnum vexillum* has been expanding its global range, significantly impacting marine habitats and aquaculture facilities. What biological features make *D. vexillum* so highly invasive? Here, we show that juxtaposed allogeneic *D. vexillum* colony fragments (‘ramets’) may, initially, form chimeric entities. Subsequently, zooids of the differing genotypes within such chimeras coordinately retreat away from fusion zones. A few days following such post-fusion retreat movements there is further ramet fission and the formation of zooid-depauperate tunic zones. Using polymorphic microsatellite loci to distinguish between genotypes, we found that they were sectorial at the fusion zones and the subsequent ramet movements resulted in further spatial separation of the paired-genotypes indicating that the fusion events observed did not lead to formation of long-term, stable chimeras. Thus, movements of *D. vexillum* colony ramets from initial fusion zones lead to progressive segregation of genotypes probably minimizing potential somatic/germ-cell competition/parasitism. We speculate that relatively fast (≤10 mm/day) movement of *D. vexillum* colonies on substrates along with frequent, and perhaps unrestrained, transient allogeneic fusions play significant roles in this species’ striking invasiveness and capacity to colonize new substrates.

## Introduction

Historic, and on-going, biological invasions that have accompanied human activities have resulted in many contemporary ecosystems including a significant percentage of non-native species (Ruiz et al., 1997; Kolar & Lodge, 2001; Arim et al., 2006; Ricciardi, 2007; Lowry et al., 2013). The ecological changes associated with the introduction of such non-native species are sometimes profound and may eventually have economic and social consequences for humans (Bax et al., 2003). Consequently, an understanding of biological invasion processes and those organismal traits displayed by highly successful invasive organisms has become the focus of much interest. Typically researchers have explored the ecological, genetic (Pérez-Portela, Turon & Bishop, 2012) and life-history traits of highly invasive species (Ruiz et al., 1997), with less emphasis being placed on behavioral (Holway & Suarez, 1999) and immunology associated traits (Lee & Klasing, 2004), including allorecognition processes (Payne, Tillberg & Suarez, 2004). This is particularly true for organisms, where invasive species out-compete native species using competitive traits associated with space acquisition during inter-specific interactions (Lowry et al., 2013; Gamradt, Kats & Anzalone, 1997; Arens et al., 2011), while attenuating competitive intra-specific interactions (Tsutsui et al., 2000; Tsutsui, Suarez & Grosberg, 2003; Torchin et al., 2003; Mangla et al., 2011). Nonetheless, to date, only a limited number of simple generalisations have emerged regarding those traits that favour the bioninvasive propensity of organisms (Kolar & Lodge, 2001; Arim et al., 2006; Hastings et al., 2004; Simberloff et al., 2013).

Due to their ability to thrive in human-influenced coastal environments, along with their proficiency as strong sedentary competitors, a number of ascidian species have, in historical times, expanded well beyond their ‘original’ (>200 years ago) geographic boundaries and thence to negatively impact locally adapted sessile marine invertebrate communities (Dijkstra, Sherman & Harris, 2007; Lambert, 2007; Carman et al., 2010; Adams et al., 2011; Smith et al., 2012) sometimes inflicting major damage to the impacted ecosystems (Molnar et al., 2008; Simkanin et al., 2012; Aldred & Clare, 2014; Zhan et al., 2015). Species within seven ascidian genera, *Botrylloides, Botryllus, Ciona, Didemnum, Eudistoma, Microcosmus* and *Styela* (Aldred & Clare, 2014; Zhan et al., 2015) are the most prominent bioinvasive ascidian taxa, and Fitridge et al. (2012) listed eleven ascidian genera that contain taxa classified as ‘nuisance’ for aquaculture. General characteristics of these invasive genera are (i) very rapid growth (ii) relatively short times to reach sexual maturity (often in combination with hermaphroditism) (iii) repeated reproductive seasonality (iv) asexual reproduction in colonial species (Aldred & Clare, 2014) and (v) some show mobility across substrate surfaces (Rinkevich & Weissman, 1988; Rinkevich & Fidler, 2014).

Probably native to the north-west Pacific ocean (Lambert, 2009), *Didemnum vexillum* (Kott, 2002), an encrusting colonial ascidian, has expanded its global distribution over the past three decades and significantly impacted both natural habitats and aquaculture facilities (Smith et al., 2012; Lambert, 2009; Stefaniak et al., 2012). This rapid range expansion raises intriguing questions as to which biological traits confer such bioinvasive success (Smith et al., 2012; Simkanin et al., 2012; Lenz et al., 2011; Bullard & Whitlatch, 2009). Invading temperate and cool water regions across the globe (to date a recording in southeast Alaska marks the northernmost latitude of this species; (Miller, 2016)), *D. vexillum* reproduces, both sexually and asexually, very rapidly and fouls marine habitats from 0 to 80 m depth (Rinkevich & Fidler, 2014; Lambert, 2009; http://woodshole.er.usgs.gov/project-pages/stellwagen/didemnum/index.htm).

The morphology of *D. vexillum* colonies varies with substrate type and colony size, with some colonies forming flat, encrusting mats that overgrow adjacent sessile organisms, while other colonies develop lobes and tendrils which easily detach thereby accelerating asexual reproductive spread (Rinkevich & Fidler, 2014; Morris & Carman, 2012; Reinhardt et al., 2012). Furthermore, adjacent allogeneic *D. vexillum* colonies can fuse to form genetically chimeric entities following tunic-to-tunic contact (Smith et al., 2012; Rinkevich & Fidler, 2014). Such fusion processes may provide an efficient substrate acquisition mechanism or provide increased intra-colony genetic diversity that is advantageous in unpredictable environments (Rinkevich, 2004; Rinkevich & Yankelevich, 2004; Rinkevich, 2011).

Here we describe processes associated with inter-colony fusion and ephemeral chimerism between allogeneic *D. vexillum* colonies. More specifically, chimeric entities were generated by juxtaposing *D. vexillum* colony ramets, and the subsequent persistence of the two colony genotypes followed using polymorphic microsatellite loci. In addition we report high mobility (≤10 mm/day) of apparently sessile *D. vexillum* colonies which may be a critical characteristic responsible for rapid substrate procurement and associated bioinvasive success.

## Materials and Methods

### *D. vexillum* colony collection and maintenance

*D. vexillum* fragments (hereafter ‘ramets’) were harvested from submerged man-made structures (0.3–1.5 m below sea surface) in the Nelson city marina (New Zealand: 41°S, 173°E) and then maintained in the laboratory as described in Rinkevich & Fidler (2014). All collection and culturing procedures complied with the legal and ethical requirements of the New Zealand (N.Z.) government, the Cawthron Institute (Nelson, N.Z.) and University of Auckland (Auckland, N.Z.). Note that the waters of the Nelson city marina are publically owned and collection of invasive invertebrates from such waters did not require legal permits. The harvested colonies were separated by a distance of >1.0 meter in an effort to minimize the probability that colonies may be related by asexual reproduction and therefore share identical genotypes. The *D. vexillum* ramets were maintained in 16 L glass tanks with high flow-through (∼1 L/min.) unfiltered, recirculated seawater with 24 h florescent lighting. The recirculated seawater was continuously monitored and regulated (salinity: 34.5 ± 1.0 ppt; pH: 8.13 ± 0.08; water temperature: 17.7 ± 0.7 °C; ambient air temperature: 20.0 ± 1.0 °C, oxidation/reduction potential: 332 ± 21 mV), with continuous aeration provided by air stones. Ramets were fed with algal cultures, ∼100 ml/tank of *Isochrysis galbana* (∼9 × 10^6^cells/ml) provided three times/week. At the end of each experiment tanks, and associated materials, were cleaned of biofouling organisms using fresh water.

### Pairing of *D. vexillum* ramets

Small ramets (∼1.0 cm^2^) were cut from the colony edges and attached with cotton thread to 5.0 × 7.5 cm glass slides, positioned vertically in slide staining racks (Rinkevich & Fidler, 2014; Rinkevich & Shapira, 1998). Ramet surfaces were gently cleaned of surface fouling organisms using small paint brushes while their vertical positioning helped to reduce on-going accumulation of debris, faecal residues and food particles. To initiate inter-ramet fusions the paired ramets were juxtaposed on the glass slides, with their natural growing edges placed at a distance of ∼1 mm. Subsequent growth and movement of the ramets were monitored and recorded using still or time-lapse photography. For subsequent genotyping small sections were dissected from the ramets on the final day of the fusion process and these sub-samples stored (100% (v/v) ethanol, −20 °C).

### Photographic recording of fusion experiments

Set I—Still image photography: For the majority of the pairing experiments the glass plates were taken from the seawater tanks at intervals of 1–2 days and images recorded as rapidly as possible, using a Canon PowerShot G15 camera (Canon, Tokyo, Japan). The whole process of photographing and taking written notes took approximately 10 min with the *D. vexillum* immersed in shallow seawater throughout the time.

Set II—Time lapse photography: For a subset of the pairing experiments time lapse photography (1 image /5 min. over 10–12 days) was used with the camera, in a weather resistant housing, positioned on the outside of the tanks (Brinno TLC 200 Pro digital camera; Brinno, Taipei, Taiwan; infrared filtered 6 mm. CS mount lens). The time lapse camera file was exported into an image sequence using VirtualDub (http://www.virtualdub.org/), and the resulting image sequence then imported into ImageJ 1.48v (http://imagej.nih.gov/ij/) and transformed into a movie (.avi file) using JPEG compression (frame rate: 65 images/s).

### Genomic DNA purification

Ramet samples of varying size (0.2–0.8 cm^2^), containing both tunic and zooids, were placed in ∼1.0 mL 100% (v/v) ethanol which was changed twice in the subsequent week and then stored long-term at −20 °C. Total genomic DNA (gDNA) was extracted from the samples using a commercial kit following the manufacturer’s instructions (GSpin™ Total DNA Extraction Kit; iNtRON Biotechnology, Inc., Gyeonggi-do, South Korea, animal tissue protocol with a tissue lysis step of 3 h, 56 °C). The purified gDNA was stored at −20 °C with DNA concentrations determined using a nanophotometer (Implen GmbH, Munich, Germany).

### Polymorphic microsatellite loci genotyping

The five microsatellite loci used for *D. vexillum* ramet genotyping were from two sources: (i) three loci DVEX05, DVEX23 and DVEX32 (Table S1) were previously reported by Abbott et al. (2011); while (ii) two loci DVEX18 (GenBank accession number KU167099) and DVEX19 (acc. no. KU167100) (Table S1), were identified in this work using 454 pyrosequencing of *D. vexillum* genomic DNA performed by an external contractor (Ecogenics, Balgach, Switzerland). Amplification and florescent labelling of microsatellite loci derived PCR products was achieved using the three primer strategy of Schuelke (Schuelke, 2000). Thus, an 18 bp generic ‘M13-tag’ sequence (5′-TGTAAAACGACGGCCAGT-3′) was added on the 5′ end of the loci specific forward primers Schuelke (Schuelke, 2000) (Table S1). PCR mixes (10.0 µL) consisted of 1x MyTaq™ HS Mix (cat. no. BIO25045, Bioline, London, UK), 30–35 ng of template genomic DNA, M13-tagged-locus specific forward primer (0.06 µM), locus specific reverse primer (0.2 µM) and labelled M13-tag primer (0.2 µM) which as 5′ labelled with one of four alternative fluorescent dyes: 6-FAM™ (blue), VIC^^®^^ (green), NED™ (yellow), PET™ (red) (Table S1). Thermo-cycling conditions for the DVEX18 and DVEX19 loci amplifications were: 94 °C/2 min, 1 cycle ; 94 °C/30 s, 60 °C/30 s, ramping to 72 °C at +0.2 °C/s, 72 °C/30 s, 15 cycles; 94 °C/30 s, 65 °C/30 s, 72 °C/30 s, 30 cycles; 72 °C/10 min; 60 °C/30 min; 15 °C/hold. Thermo-cycling conditions for the DVEX05, DVEX23 and DVEX32 loci followed the ‘touchdown PCR’ protocol of Abbott et al. (Abbott et al., 2011): 95 °C/2 min, 1 cycle; 95 °C/30 s, 62 °C dropping 2 °C/2 cycles to 54 °C/30 s, 72 °C/30 s, 10 cycles; 95 °C/30 s, 54 °C/30 s, 72 °C/30 s, 23 cycles; 72 °C/10 min; 60 °C/30 min; 15 °C/hold. Both thermo-cycling protocols included a 60 °C/30 min step at the end, as this is thought to promote the *Taq* DNA polymerase catalyzed addition of 3′ As to the amplicons thereby reducing length heterogeneity arising from the PCR itself rather than from actual allele length variation. The fluorescently labelled amplicons were stored in the dark at +4 °C before being diluted 1:4 in milliQ water and then pooled so that each microsatellite amplicon in a ‘pool’ was labelled with a different dye and could be identified by their differing emission spectra (Table 1). Amplicon lengths were estimated by an external contractor (Massey Genome Service, Massey University, New Zealand) using capillary electrophoresis (ABI3730 DNA analyser; Applied Biosystems, Waltham, U.S.A.) and GeneScan™-500 LIZ™ size standards (Applied Biosystems). Electropherogram results were used to estimate amplicon sizes, using the software Peak Scanner™ v2.0 (Life Technologies, Carlsbad, U.S.A.).

**Table 1:** Summary descriptions of the outcomes of the *Set I* 12 pairwise ramet. The images described are shown in Fig. S1.

Ramet pair	Summary of outcomes
AxB–1	***Movement summary:*** A short fusion of ramets at Days 3–4, with the smaller genotype A ramet soon moving vertically on the glass slide, away from the larger genotype B ramet, until it reached the top of the glass slide. By Day 11, when the experiment was terminated, two widely separated zooid-containing regions were apparent. ***Genotyping summary:*** At Day 11 the majority of the dissected colony regions displayed only a single genotype, with a clear spatial separation of the A and B genotypes. However, in one remnant region (section ‘a’) both the A and the B genotypes were detected. In this pairing the smaller genotype A ramet was the more mobile and no substantial and sustained fusion of the two ramets was observed.
AxB–2	***Movement summary:*** The matrices of the A and B paired ramets were visibly fused by Day 2. Over the next few days the bulk of the fused/chimeric colony moved vertically upwards across the back of the glass slide, and by Day 8 (when the experiment was terminated) much of the fused colonial entity had moved over the top of the glass slide to the opposite side. A zooid-containing remnant region extended back to the two ramet fragments that still remained at the approximate site of the initial fusion. ***Genotyping summary:*** At Day 8 some central regions (e.g., regions ‘e’ and ‘h’) contained both colony A and B genotypes. In the part that actively migrated vertically, only the colony B genotype was detected (regions: ‘k’, ‘m’, ’n’, ‘o’), or there was largely B with only traces of the genotype A (regions: ‘l’, ‘p’). In this pairing, the genotype B region was the most mobile entity, and its movement was followed by the gradual removal of genotype A zooids from the actively migrating colony region.
AxC–1	***Movement summary:*** Both genotypes differed somewhat in colour, with genotype C being more orange, which helped trace their location in the chimera. Initially (Days 1–2), some fusion of the A and C colony matrices was visible but this fusion was transitory and by Day 3 an area largely clear of zooids was visible between the A and C ramets. Over the subsequent days both partners moved away from each other and by Day 8 they were clearly separate. ***Genotyping summary:*** Eight days from onset some remnant regions (regions: ‘t’, ‘u’, ‘v’, ‘x’, ‘y’), which appeared to largely lack zooids, displayed both A and C genotypes. However, the motile regions, which possessed many zooids, were largely composed of a single genotype, with the notable exceptions being samples ‘a’, ‘e’ and ‘f’, where both genotypes were detected. In this pairing the ramets of both paired genotypes appeared to be equally mobile.
AxC–2	***Movement summary:*** Initially the paired ramets fused, although some clear area remained between the two genotypes during the first few days, followed by a continuous area of fusion between the matrices. Both genotypes were motile and they soon began to move away from each other, a process that was followed by the development of a long, extended shape (Days 3–13). However, despite the separation movement, at Day 13 the two colonies were still connected by an extended region containing zooids. ***Genotyping summary:*** This chimera is the outcome of a large ramet from genotype C fusing with a small ramet from genotype A. At Day 13 there were a few samples (‘h’, ‘e’) where both genotypes were equally detected, and two samples (‘f’, ‘g’) where genotype A was dominant. However, in most sampled regions (*n* = 10), only the ramet C genotype was evident, and there were no dissected regions with only genotype of ramet A.
AxD-1	***Movement summary:*** A and D ramets differed somewhat in colour, with colony D being more orange, which proved helpful in following their behaviours during early fusion states. By Day 2 there appeared to be a complete fusion of the ramet A and D matrices, where both partners mixed together with no indication of one ramet region moving away from the other. At Day 7, when the experiment was terminated, a single fused colony was still apparent. ***Genotyping summary:*** At Day 7 both the ramet A and D genotypes were detected in the remnant regions (regions: ‘a’, ‘j’, ‘k’, ‘l’), which had a few zooids dispersed in transparent tunics. In the regions containing zooids (regions: ‘f’, ‘g’, ‘h’) only genotype D was detected, while I”n three other regions genotype D was clearly the most abundant (regions: ‘e’,‘d’, ‘i’). In two other regions (‘b’ and ‘c’) genotype A was the most abundant, but no sample with only genotype A was detected.
AxD-2	***Movement summary:*** Both colonial ramets differed somewhat in colour, with colony D being more orange, which proved helpful in following their movements after the first fusion events. Complete fusion of the two colonies was apparent (Day 3), and was soon (by Day 5) followed by the clearing of the region between the colonies. The D ramet was highly mobile, and after a few days (days 7–12) it migrated vertically towards the top of the glass slide and then over to the other side of the slide. The A ramet was relatively stationary at the beginning and then more motile in the last few days. At the end of the experiment (Day 12) there was very clear separation of two distinct zooid-containing regions. ***Genotyping summary:*** Six regions (‘i’–‘n’) were largely composed of genotype D zooids, although traces of genotype A were detected in all these regions. In contrast, in regions ‘d’, ‘e’ and ‘f’ only genotype A was detected and in the adjacent sample ‘c’ only traces of genotype D were found. In this pairing ramet D was the more mobile although the ramet A genotype was also motile.
BxC–1	***Movement summary:*** Genotypes B and C differed in colour, with colony C being more orange, which assisted in following their movements at early stages. Visually there appeared to be a complete fusion of the B and C colonies by Day 2. The fusion appeared to persist for all seven days, with the chimeric entity being motile with no strong indication of the B and C genotypes moving away from each other. Some areas in the chimera were cleared of zooids. ***Genotyping summary:*** At Day 7, some of the remnant regions without many zooids (regions: ‘f’, ‘g’, ‘h’, ‘l’) contained both the ramet B and C genotypes. Zooid-containing regions with both genotypes (regions: ‘d’, ‘e’, ‘h’, ‘i’, ‘j’, ‘m’, ‘o’) were detected while at the leading/fast moving edges of the chimera a single genotype was detected in most of the samples (genotype C in regions ‘a’, ‘b’, ‘c’, ‘k’; genotype B in regions ‘n’, ‘p’, ‘q’). Thus, although the fused colony appeared to moving together nonetheless there remained segregation of the B and C genotype zooids within the colony.
BxC–2	***Movement summary:*** By Day 3 of the experiment the tunic matrices of the ramet B and C genotypes came into contact and the fusion that followed appeared limited. From Day 3 onwards the region corresponding to ramet B became highly mobile, moving to the top of the glass slide and then over to the other side of the slide. The region corresponding to ramet C was relatively immobile except for the development of an extension of zooid containing matrix, which moved in the same direction as colony B and attached to colony B. By Day 12 there were two clearly distinct zooid-containing regions, with one located on both the top and ‘reverse’ side of the glass slide and the other still largely in the initial location of the pairing, albeit with an extension stretching towards the genotype B colony. ***Genotyping summary:*** Consistent with the observed morphological movements, in regions ‘h’–‘l’ genotype B was either the dominant or exclusive genotype detected. In contrast, in regions ‘a’–‘g’ genotype C was the dominant genotype detected. In two regions (regions: ‘i’ and ‘g’) both genotypes were detected. In this pairing the genotype B ramet was the more mobile, although genotype C ramet was also somewhat motile, with one section apparently actively fusing with ramet B.
BxD-1	***Movement summary:*** The B and D ramets differed somewhat in colour, with colony D being more orange, which assisted in following their movements. Both ramets had clearly fused and intermixed by Day 2. The entire fused colony then moved together in a coordinated manner until it straddled the top edge of the glass slide. By Day 9 there was an indication that the areas of the B and D genotypes had started to separate, and so by Day 12, when samples were taken for genotyping, the previously fused colony had already separated into two major regions which, on the basis of their colour, were largely composed of B or D genotype zooids. ***Genotyping summary:*** At Day 12 there were a number of mixed regions that contained significant amounts of both the B and the D genotypes (regions: ‘j’–‘o’). At the edges of the zooid-containing regions sampling revealed areas with exclusively a single genotype: genotype B in samples ‘p’, ‘h’, ‘g’ and genotype D in sample ‘f’. Some regions contained both ramet genotypes although one was very minor proportion.
BxD-2	***Movement summary:*** B and D ramets differed in somewhat colour, which assisted in following their movements. After two days there was a clear fusion between the two ramets. From Day 3 onwards part of the ramet D region (identifiable by its relatively orange colour) began to migrate away from the fusion area, forming a separate region. One region migrated vertically, moving over the top of the glass slide by Day 7. ***Genotyping summary:*** Consistent with direct observation, both ramet B and D genotypes were detected in almost all of the regions dissected on Day 7. In samples where both genotypes were detected consistently, one genotype was always much more abundant –the exception being samples ‘k’ and ‘q’ where both genotypes were approximately equally abundant. Only three samples (‘p’, ‘i’, ‘j’) contained only a single genotype, genotype B. Thus, although genotypes B and D fused and moved together, there was a segregation of the B and C genotypes within the developing chimera. One section of the ramet D genotype moved away from the ramet B genotype while the other parts remained closely associated with genotype B.
CxD-1	***Movement summary:*** The C and D ramets came into contact and fused by Day 3, while part of ramet D separated from the remainder of ramet D and migrated vertically to the top of the slide. The part of ramet D that fused with ramet C formed an apparently quite stationary chimera remaining fused until Day 12. The migrating portion of ramet D continued to the top of the slide and over to the other side. ***Genotyping summary:*** The highly mobile section of ramet D that separated before fusion occurred was composed entirely of genotype D (regions: ‘a’, ‘b’, ‘c’, ‘d’, ‘e’, ‘o’). The chimeric colony was mainly composed of the ramet C genotype, although regions with significant amounts of ramet D genotype were detected (regions: ‘f’, ’g’, ‘l’, ‘m’, ‘n’).
CxD-2	***Movement summary:*** The C and D ramets fused, resulting in a chimeric colony that stayed mostly at its initial location. The chimera grew slowly on the substrate, with some clearer areas and ramet intermingling. By Day 7, when the chimera was genotyped, there was an indication that the chimeric colony divided into two sections that started to move apart in opposite directions. ***Genotyping summary:*** Despite the formation of a seemingly mixed chimeric entity, at Day 7 most of the samples revealed only a single detectable genotype (genotype D: regions ‘a’–‘g’; genotype C: regions ‘l’, ‘m’, ‘n’, ‘p’). Samples taken from the center of the chimera revealed a mixture of both genotypes, with the ramet C genotype largely dominant and some traces of the D genotype (regions ‘k’ and ‘o’). Thus although the ramets had formed a chimera, the B and C genotypes remained largely distinct.

To discriminate between the two genotypes that potentially contribute to a particular *D. vexillum* entity, we required the two genotypes to differ by a minimum of one allele at a minimum of two microsatellite loci (Tables S1, S2). The specific loci used in the pairings studied in detail are summarised in Table S2. The relative heights in the electropherogram traces of the genotyping discriminating alleles were used to provide semi-quantitative estimations for the relative amounts of the two genotypes in the gDNA preparations from sections taken from the chimeric entities and, by implication, of the relative biomass of the two genotypes in the chimeric entities. The relative genotype ratios, as deduced from the electropherogram traces, were summarised using the following symbolism where X and Y denote the two different genotypes: X∼Y, approximately equal amounts of both genotypes; X/0 or 0/Y, one of the genotypes not detected; X>Y or Y>X, one of the two genotypes clearly more abundant then the other and X≫Y or Y≫X, one genotype is much more abundant but there is a trace of the other genotype.

### Estimation of ramet surface areas

Digital photographs of the slides from the day of initial fusion and the day of experimental termination were processed using the area/length analysis tool of the NCRI CPCe software (Kohler & Gill, 2006). At day of fusion, the outline of each fragment from either *D. vexillum* ramet was manually traced and converted by the software into surface area values (cm^2^), a protocol employed at the end of the experiments for each sample within a chimera. All the CPCe output files were exported to Microsoft Excel 2016. Then, we imposed the microsatellites’ semi-quantitative ratio results on the area calculations, as follow: a single genotype detected in the sample (X or Y; 100% of the fragment’s surface area to the appropriate genotype), X∼Y (both genotypes in the tissue sample revealed approximately the same DNA amounts, 50% surface area for each genotype), X>Y or Y>X (75% and 25% of the surface area, correspondently) and X≫Y or Y≫X (95% and 5% of the surface area, correspondently). Next, a cumulative surface area / genotype for each chimeric combination was computed, not including the ‘nd’ cases where the microsatellite based discrimination had not been successful. Statistical analyses were performed using the IBM SPSS Statistics software for Microsoft Windows, version 23. After determining normal distribution for all genotypes a one-way analysis of variance (ANOVA) test with internal pairwise comparisons was used to determine the differences between the mean changes per day for the *D. vexillum* genotypes.

## Results

Two sets of experiments were conducted. In the first set, denoted *Set I* (six allogeneic pairwise combinations, all in duplicate), *D. vexillum* movements were documented by taking daily still photographs. In the second set, denoted *Set II* (two allogeneic pairings), time-lapse photography was used. Observations were performed for 1–2 weeks subsequent to observed apparent chimerism (i.e., tunic fusion), followed by the sectioning of the *D. vexillum* colonies to determine the distribution if the paired genotypes.

### Set I: single frame photographic records of *D. vexillum* inter-colony fusion processes

**Figure 1:**
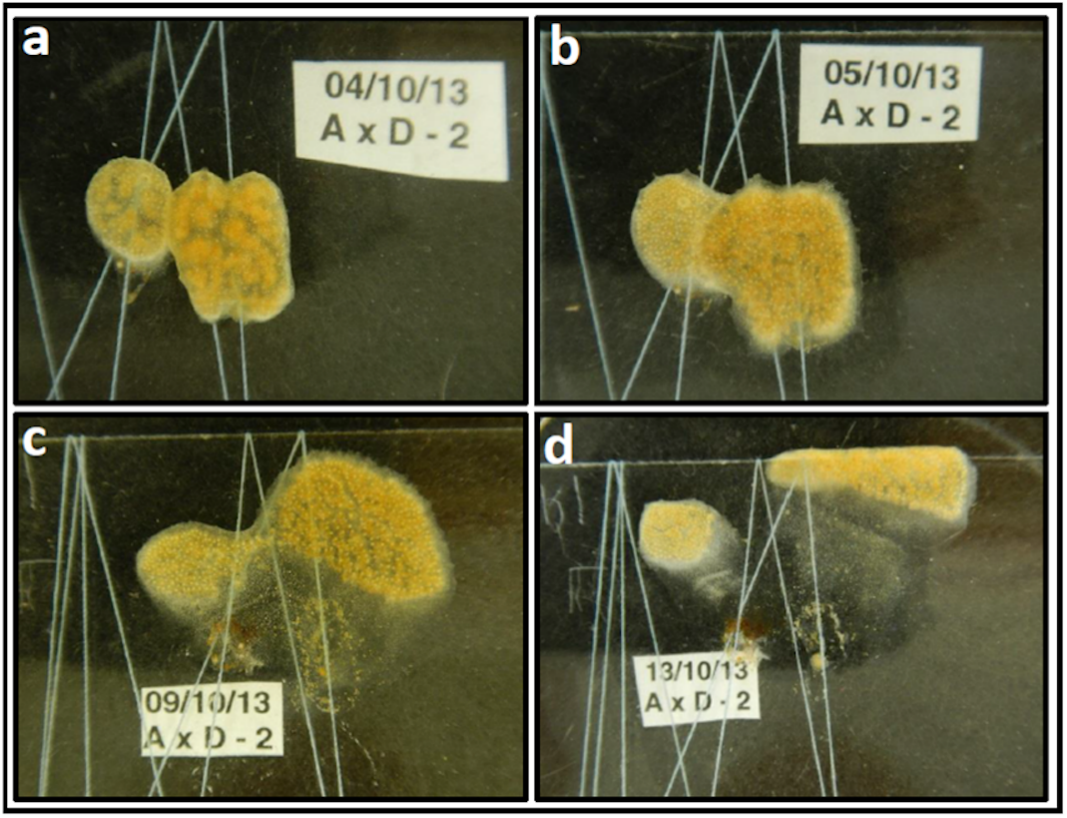
*Didemnum vexillum* ramet pairing experiment example: A x D combination (replicate no 2). The *D. vexillum* ramet pairing shown provides an example of how images were generated by still photography and how a descriptive narrative was derived from such images. This example was selected because the two ramets genotypes differed somewhat in their colour and so were more easily discriminated. (A) One day following contact between the paired ramets (A, mustard-coloured, D, more reddish) there is clearly area of tunic fusion; (B) The following day, a wide frontal zone of fusion was visible but both colour morphs are still clearly distinguishable; (C) six days after the initial ramet fusion, a narrow zone of fused tunic remains visible but the two ramet regions have clearly separated with the ramet D zooids moving upwards/right; (D) nine days following fusion, both the A and D ramet regions have moved away from the site of the initial interaction/fusion. The following day the whole *D. vexillum* area was dissected (Fig. S1) and microsatellite genotyping confirmed that the left region contained the ramet A genotype, most sections on the right contained the genotype of ramet D genotype while the central degenerating/translucent tunic areas had both the A and D genotypes in varying ratios.

Using ramets taken from four (termed: A to D) putatively allogeneic colonies, allogeneic and isogeneic pairings were established. Six possible pairwise combinations of colony ramets (i.e., AxB, AxC, AxD, BxC, BxD, CxD), along with their isogenic pairing controls, were established in duplicate (*n* = 12 allogeneic pairings in total) as described in the materials and methods. Observations and still photographs of the paired ramets were taken on a daily basis. The photographic records of all 6 allogeneic pairings are shown in Fig. S1 with summary written descriptions provided in Table 1. *Set I* pairing experiments were terminated seven to twelve days after initial pair establishment and sub-sections of the *D. vexillum* distributed on the slides were genotyped using polymorphic microsatellite loci thereby allowing semi-quantitative determination of the spatial distribution of the paired ramet genotypes (Fig. S1). As an example Fig. 1 shows images from four separate days following a ramet A vs. ramet D pairing and is illustrative of the general phenomena observed in such allogeneic pairings (Fig. 1). What follows is a summary of the observations made from the ramet pairings and generalisations that emerge (Table 1, Fig. S1). In all ramet pairings (iso- and allogeneic combinations) tunic contacts occurred one to three days after initiation of the experiment and, in some cases, such tunic contacts were followed by apparent fusion of the two ramet matrices. All the isogeneic paired ramets fused to form single colonies. Initial fusions between allogeneic ramets were followed by either morphologically-mixed chimerism (i.e., where zooid containing regions from both partners appeared to be intermingled with each other) or by visibly-sectorial chimerism (i.e., where only very limited mixing of zooid containing regions was observed) (Fig. 1, Table 1, Fig. S1). Whatever the initial degree of chimera mixing, shortly after tunic fusion (∼1–2 days), one or both of the paired ramets started coordinated movement which were apparently directionally away from the fusion zone (Fig. 1C, Fig. S1). The coordinated movement of zooid containing regions was followed by the formation of zooid-depauperate areas which appear as translucent zones that clearly lack the mustard-coloured zooids (Fig. 1, Fig. S1). Such regions, lacking large numbers of zooids, appear to degenerate quite quickly (∼2–3 days after fusion), in contrast with the opaque mustard-coloured tunic regions which contain many zooids and remain apparently healthy. In some cases the gliding-movements of zooid clusters across the glass substrate have gone as far as the glass plate edges and indeed even over to the opposite side of the glass slide (Table 1, Fig. S1), resembling the retreat growth phenomenon in interacting allogeneic pairs of the colonial tunicate *Botryllus schlosseri* (Rinkevich & Weissman, 1988). The sliding movements of the zooid-rich regions and the associated generation of zooid-depauperate zones led in many cases to the splitting of the chimeric entities into two non-equal parts seven to twelve days after initial tunic fusions. Isogeneic *D. vexillum* isogeneic pairs, while fused in the same way as the allogeneic pairs and showed fast growth and movement on the substrates, did not retreat from each other, and when splitting into daughter subclones following fast growth in every direction, these events occurred haphazardly, in different parts of the growing colonies, unrelated to the former fusion sites. It is also evident that chimerism had no apparent impact on either ramet’s survivorship, as all pairs survived over the period of observation.

To assess the degree of genetic chimerism, at the end of the fusion experiments the *D. vexillum* ramets, including both the translucent zones and the zooid-rich regions, were sampled (typically as 12–25 fragments, Fig. S1) for genotyping at polymorphic microsatellite loci that discriminate the two genotypes of the paired ramets. The genotyping results for each pairing experiment are shown in detail in Fig. S1. What follows is a summary of generalisations that emerge from the genotyping and the associated semi-quantitative estimation of genotype ratios in the dissected regions. It appears that in most, perhaps all, of the pairings, the initial chimerism arising from tunic fusions is followed by what appears to be a genotype-based ‘depuration’ process whereby the two genotypes associated with the chimera actively spatially separate from each other. Firstly, zooid intermingling is restricted to the fusion border areas and does not involve the intermixing of genotypes throughout the fused entity (Fig. S1). Secondly, and subsequent to the tunic boundary fusions, one or both of two interacting genotypes start to grow away from the zooidal-intermingled areas to form zones with exclusively, or nearly exclusively, a single genotype. The associated splitting of the formerly chimeric entity into two or more fragments generates translucent, zooid-depauperate zones which contain a mixture of both the paired genotypes (Fig. S1). Note that throughout the observed processes there was no clear evidence of any morphological allorejection type events or of general tissue damage, an observation that should be interpreted with caution as it may simply reflect the low level of magnification used in this study. In addition, replicates of the same pair-wise combinations often have somewhat differing outcomes (Table 1, Fig. S1), suggesting a non-genetic and non-hierarchical nature to the fusion and subsequent depuration processes.

We evaluated each genotype’s total surface area (cm^2^) at both the onset and termination of pairing experiments (Table 2). Then, we calculated percentage changes in area/day for each genotype in each chimera (Table 2). On the days of fusion ramet sizes had a size range of 0.4–3.5 cm^2^ with most 1.0–2.3 cm^2^. At the termination of the experiments (i.e., after 7–13 days) the ramets, occupied 1.3–15.1 cm^2^ of surface area, with most in the range of 3.6–7.9 cm^2^, reflecting a very wide range of 4.5–112.1% increases in area/day with most (13/22) ramet areas increasing at rates of 34–77%/day (Table 2). The ‘nd’ samples were limited and few, representing 1.2–3.1% of the total surface areas (combinations AxC-1, BxD-2 and CxD-2) and 10.2% of the total surface area for combination AxB-2 (Table 2).

**Table 2:** *Didemnum vexillum* experimental *Set I* ramet size increases. Ramets A–D were paired in six pairwise combinations in duplicate and percentage area/day for each ramet was determined. nd - not done. Values for the last days of observations were calculated from the cumulative relative distributions of genotypes (as determined by microsatellite genotyping) in all tissue samples for each chimera. Refer to Fig. S1 for the detailed information on the relative distributions of genotypes in each tissue sample.

Pair #	Days	Size at start		Size at end		nd	Total change (%)		Change/day (%)	
		Left	Right	Left	Right		Left	Right	Left	Right
A–B–1	10	1.1037	1.6224	1.60295	5.34125	0	49.93	371.89	4.99	37.19
A–B–2	8	0.9929	0.8141	1.381375	3.563225	0.5644	38.85	274.91	4.86	34.36
A–C–1	8	2.1269	1.6891	3.161315	5.560285	0.1138	103.44	387.12	12.93	48.39
A–C–2	13	0.4042	1.8311	1.267475	7.670625	0	86.33	583.95	6.64	44.92
A–D–1	7	0.4605	0.8808	1.43135	3.30235	0	97.09	242.16	13.87	34.59
A–D–2	11	0.6103	0.9309	2.714945	5.850655	0	210.46	491.98	19.13	44.73
B–C–1	7	1.7052	1.3678	5.701515	6.755085	0	399.63	538.73	57.09	76.96
B–C–2	10	3.4646	1.0199	7.87965	4.91385	0	441.51	389.40	44.15	38.94
B–D–1	12	1.6587	0.9411	15.10905	7.1159	0	1345.04	617.48	112.09	51.46
B–D–2	7	1.8035	1.1356	3.568155	4.543045	0.2653	176.47	340.74	25.21	48.68
C–D–1	10	1.4454	2.3625	2.826825	6.937175	0	138.14	457.47	13.81	45.75
C–D–2	7	0.7603	0.9377	2.27894	1.71616	0.1339	151.86	77.85	21.69	11.12

As no hierarchy in growth patterns was documented when aligning the growth rates with genotypic combination, the average genotypic growth rates/genotype was performed, averaging the 6 ramets from each genotype. Results (genotypes: *A* = 10.4 ±5.3 cm^2^; *B* = 51.7 ± 28.7 cm^2^; *C* = 40.8 ±20.3 cm^2^; *D* = 39.4 ± 13.7 cm^2^) showed that genotype A represents the slowest growth rates (∼0.25 of genotypes B, C, D) but statistically significantly different from only genotype B (ANOVA, *p* = 0.0165).

### Set II: time-lapse photographic records of *D. vexillum* inter-colony fusion processes

**Figure 2:**
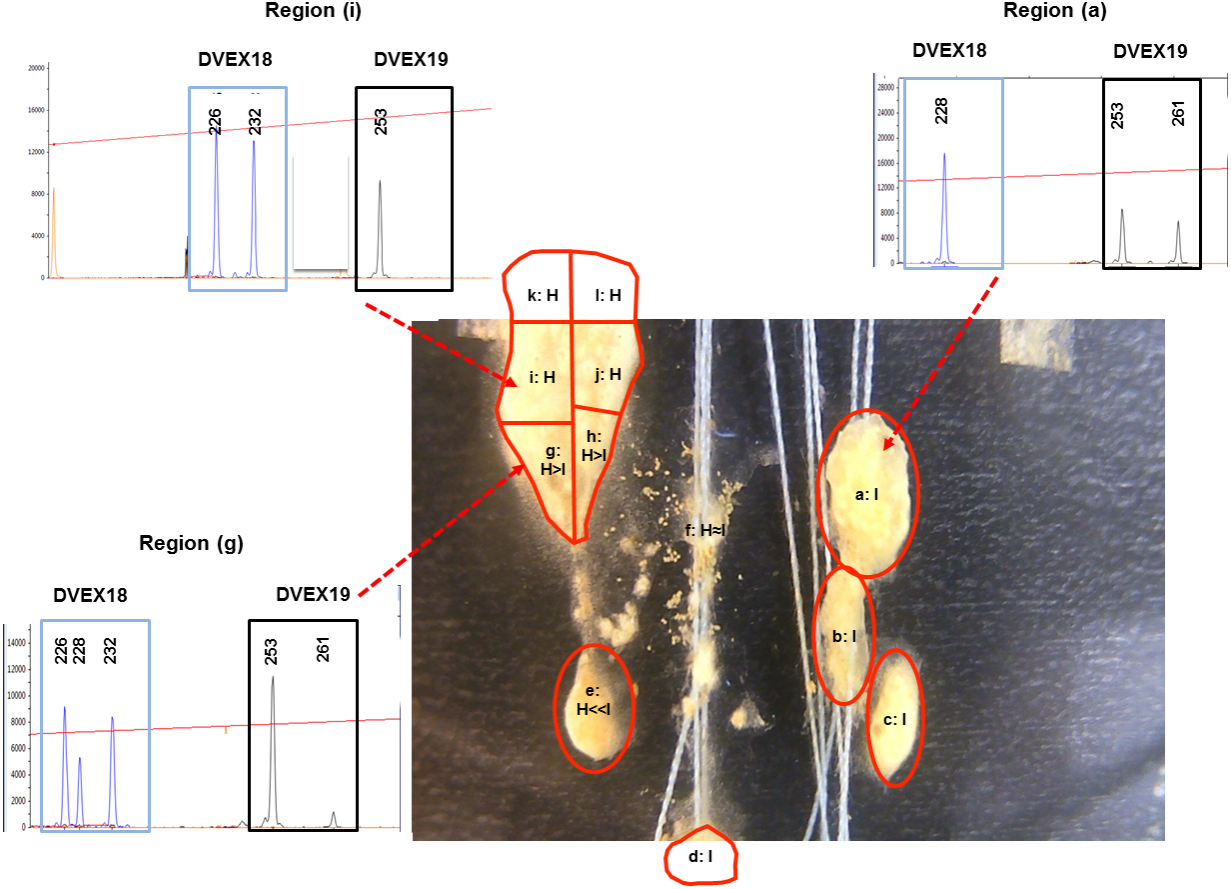
*D. vexillum* genotyping at microsatellite loci. An example of the genotyping of dissected regions at the end of a ramet pairing experiment (ramet pairing *Set II*; combination H x I). Each dissected region (labelled a–l) was genotyped at two microsatellite loci (DVEX18 and DVEX19; Table S2) at which the ramet H (heterozygous DVEX18_226∕232_; homozygous DEX19_253∕253_) and ramet I (homozygous DVEX18_228∕228_ heterozygous DEX19_253∕261_) genotypes differ by at least one allele. Shown are electropherogram traces of the DVEX18 and DVEX19 loci in three dissected regions (a), (i) and (g). The proportion of the H and I genotypes can be estimated from the heights of those peaks corresponding to those alleles that discriminate the two ramet genotypes. Region (a) displayed only the alleles of the ramet I genotype and region (i) only those of ramet H. In contrast region (g) has alleles from both ramet genotypes although the H genotype peaks are dominant.

To obtain a higher resolution description of *D. vexillum* inter-colony fusion and associated mobility we used time lapse photography. As is *Set I*, *D. vexillum* ramets were paired on glass slides and then filmed using a camera placed outside the seawater tanks in which the colonies were maintained. High quality time lapse records were obtained for two allogeneic pairwise combinations (made of ramets of genotypes H, I, L), denoted H x I and H x L. that are analysed here and can be viewed using the supplementary movie files: movie-S1 (H x I) and movie-S2 (H x L). Single frame images separated by 24 h/1 day increments were selected to help summarise the fusion/mobility processes: H x I, Fig. S2; H x L, Fig. S3, For the H x I pairing, where the partner genotypes could be distinguished by two microsatellite loci (Table S2), 12 dissected regions were taken 12 days after the pairing establishment (Fig. 2).

In both the H x I and H x L pairings the edges of the two paired ramets came into visible contact after ∼2–3 days (Figs. S2 and S3, images on Days 2 and 3). However events subsequent to this initial contact differed strikingly between the two pairings. In the H x I combination there was, at least initially, clear fusion of the two ramet matrices and an apparent intermingling of zooids (Fig. S2, Days 3, 4, 5; movie-S1 seconds ∼00:03–∼00:08). By ∼day 5, the H/I chimeric entity started separating into two physically distinct sections in association with retreat movement of at least one or both of the ramet types, with the result that by days 6–7 the fragmentation of the transitory H/I chimeric entity was clearly apparent, resembling an ‘indifference’ reaction (Rinkevich & Weissman, 1992) or the ‘non-fusion’ reaction in botryllid ascidians (Tanaka & Watanabe, 1973). From Days 8–12 ramet fragments were observed moving in a variety of directions, with the movement directions strongly suggesting an active separation process. The separating fragments were separated by a remnant zone largely devoid of zooids while the region corresponding to ramet ‘H’ was moving over the edge of the glass slide to the other side (Fig. S2, Movie S1).

In contrast to H/I combination, the H/L combination does not display any, even transitory, apparent intermingling of the zooid types in a single matrix (Fig. S3, Movie S2). Indeed after ∼2 days of close contact the genotype L ramets started displaying a coordinated movement away from the H ramet that resulted, within 6 days, in only remnant tunic at the previously contacting areas (Fig. S3 Days 3–6, Movie S2 seconds ∼00:01–∼00:09). As with the H x I combination the movements of the ‘H’ and ‘L’ ramets strongly suggest some sort of active separation/avoidance process (Fig. S3, Movie S2). For the H/I pairing it proved possible to discriminate the ‘H’ and ‘I’ genotypes using the microsatellite loci DVEX18 and DVEX19, both of which differed between the two genotypes by at least one allele (Table S2) and to obtain a semi-quantitative measure of the ratios of the two genotypes in the sections taken upon termination of the fusion experiment (Day 12, Fig. 2). Such genotyping results showed that five small zooid-rich regions that emerged from the transitory fused/chimeric entity colony (denoted regions a–e in Fig. 2) were largely composed of zooids of genotype ‘I’, while the others were largely of genotype ‘H’. Two of the apparently decaying regions that remained behind the highly mobile regions (denoted nos. d, e in Fig. 2, Fig. S2) were mainly of the ‘I’ genotype while a third remnant region (fragment f; Fig. 2, Fig. S2) was composed almost equally of both, the ‘H’ and ‘I’ genotypes.

## Discussion

The colonial tunicate *Didemnum vexillum* (Aplousobranchia: Didemnidae), native to the northwest Pacific Ocean (Smith et al., 2012; Lambert, 2009; Stefaniak et al., 2012), was first described by Kott (2002) in New Zealand, where it had become relatively widespread over the preceding decade. Over the subsequent two decades *D. vexillum* has been reported in cool temperate coastal waters throughout the world, including both North American coasts, the Atlantic coast of Europe and also the North Sea (Smith et al., 2012; Lambert, 2009; Stefaniak et al., 2012), where it is successfully out-competing other epifaunal and macrofaunal taxa and thereby significantly impacting the species compositions of natural and anthropogenic benthic communities. Furthermore, a recent report of *D. vexillum* being established in the Mediterranean sea suggests adaptation to warmer waters, with the implication that *D. vexillum* could become even more widespread globally (Ordóñez et al., 2015), if one considers that this species has adapted to the euhaline and tidally well-flushed zones of the Venetian lagoon, Italy (Tagliapietra et al., 2012).

Recruitment of *D. vexillum* is achieved through sexual reproduction and larval settlement (Fletcher, Forrest & Bell, 2013) or via asexual reproduction by colony fragmentation (Stefaniak & Whitlatch, 2014). Indeed the remarkably fast spread of this species is consistent with field and laboratory observations attesting that *D. vexillum* colonies split frequently and separated fragments easily reattach and form new colonies (Rinkevich & Fidler, 2014; Morris & Carman, 2012; Valentine et al., 2007). At least in some locations, *D. vexillum* colonies grow to form tendril-like extensions that readily detach to potentially form new colonies (e.g., Reinhardt et al., 2012; Stefaniak & Whitlatch, 2014).

In common with many other colonial tunicates, adjacent *D. vexillum* colonies can fuse, at least transiently, to form chimeric colonies (Smith et al., 2012; Rinkevich & Fidler, 2014; Sellers, Fagerberg & Litvaitis, 2013, this study). While in botryllid ascidians, allorecognition between colonies is strongly influenced by highly polymorphic genetic loci (Rinkevich, 2004; Rinkevich, 2011; Rinkevich & Weissman, 1992; Rinkevich & Weissman, 1987; Rinkevich, 1996), for *D. vexillum* itself little is known on genetic mechanisms associated with chimeric colony formation (Smith et al., 2012; Rinkevich & Fidler, 2014; Sellers, Fagerberg & Litvaitis, 2013, this study). Moreover, fusion in botryllid ascidians begins via vascular anastomoses (‘cytomictical chimerism’; sensu (Rinkevich & Weissman, 1987)), which enables stem cells circulating throughout the chimeric colony to initiate what is, in effect, ‘cell lineage competition’ between the two genotypes in the chimera. In contrast, in aplousobranch compound ascidians, like those in the genus *Didemnum,* the tunic-embedded zooids are not connected by blood vessels and thus chimerism (i.e., zooids of differing genotypes sharing the same tunic matrix) is not associated with extensive exchange of blood/stem cells throughout the chimeric colony (Bishop & Sommerfeldt, 1999). Fusion of *D. vexillum* colonies likely involves a definable allorecognition reaction as the most peripheral edges of *D. vexillum* colonies are characterised by small extensions that contain distinct aggregates of cells (Rinkevich & Fidler, 2014). In addition, during allogeneic fusions there are major changes in the abundance and distribution patterns of tunic cells (Sellers, Fagerberg & Litvaitis, 2013). More specifically, phagocytic and morula cells congregate at areas of inter-colony contact as do elevated numbers of bladder and filopodial cells (Sellers, Fagerberg & Litvaitis, 2013). The possible exchange of such cell-types, or the possibility of other migratory stem cells moving out from zooids into the common tunic, may provide the possibility of somatic/germ cell parasitism, a phenomenon that was recorded for botryllid ascidians (Sabbadin & Zaniolo, 1979; Pancer, Gershon & Rinkevich, 1995; Stoner, Rinkevich & Weissman, 1999) but was not analyzed in the present study. Thus, it is plausible that cell lineage competition exists between *D. vexillum* genotypes in chimeras and therefore it is reasonable to expect that transient chimerism is one of the mechanisms that avoid or attenuate such competition.

Allogeneic fusions between sedentary marine organisms may be ecologically adaptive, since such fusions could result in organisms that are larger, more genetically diverse, and, potentially, more competitive (Rinkevich & Yankelevich, 2004). Several studies (Rinkevich, 2004; Rinkevich & Yankelevich, 2004; Rinkevich, 2011; Rinkevich & Weissman, 1992; Rinkevich & Weissman, 1987; Rinkevich, 1996; Buss, 1982; Grosberg & Quinn, 1986; Rinkevich, 2002) offer a list of ecological and evolutionary benefits that can be attributed to chimerism. Prominent in the list of benefits are: (i) enhanced genetic variability, (ii) establishment of synergistic complementation, (iii) a guaranteed mate location and (iv) a list of beneficial size-related traits (e.g., increased growth rates, enhanced reproduction and heightened survivorship, improved stability against predation and partial mortality, as well as against harsh environmental conditions) (Rinkevich, 2004; Rinkevich, 2011; Buss, 1982; Grosberg & Quinn, 1986; Rinkevich, 2002). The predicted major potential cost of such chimerism is thought to be the threat of cell lineage competition between genotypes especially for positions in the germ cell compartment (Rinkevich, 2004; Rinkevich, 2011; Buss, 1982; Grosberg & Quinn, 1986). Therefore, it is noteworthy that the *D. vexillum* transitory fusion phenotype did not appear to lead to a mix of zooids/genotypes across the whole colony entity as would be expected of a long-term, stable chimera. Microsatellite genotyping supported the conclusion that each partner largely maintained its genetic homogeneity and it was primarily the fusion zone that contained both genotypes. Thus, the *D. vexillum* fusion/separation phenomenon somewhat resembles the ‘Transitory Fusion (TF)’ process described in the hydroid *Hydractinia* (Buss, 1982).

Another major finding of this study was to document the rapid (≤10 mm/day) movement of *D. vexillum* colony fragments following transient inter-colony fusion events. Such movements, resembling the retreat growth phenomenon of *Botryllus schlosseri* interacting allogeneic pairs; , probably represent a very effective mechanism for rapid substrate acquisition and general spreading (Birkel, Cheng & Lewin, 1981; Carlisle, 1961; Van Thinh, Griffiths & Ngan, 1981; Cowan, 1981). Thus, the transient chimerism observed in *D. vexillum* is more of chronic rejection types documented in botryllid ascidians (such as separation, morphological resorption and retreat growth (Kolar & Lodge, 2001; Rinkevich, 2004; Rinkevich & Weissman, 1992; Rinkevich & Weissman, 1987)) than of acute rejection types (such as bleeding, points of rejections, necrotic areas (Rinkevich & Weissman, 1992; Rinkevich, 2002)). Further, all interacting ramets that were observed exhibited increased occupied surface area/day although the percentage increases varying widely (range: 4.5–112.1%) with most displaying rates of surface area increase of 39–52%/day. In interpreting this data it should be cautioned that the associated genotyping methodology is based on semi-quantitate measurements of relative electropherogram peak heights corresponding to PCR amplicons from different microsatellite alleles and it is not clear whether there is an associated significant increase in *D. vexillum* zooid numbers, or indeed total biomass, over the observed period. Rather the mobile, spreading ramets are characterized by trailing regions of less densely compacted zooid distribution and by the appearance of zones, adjacent formerly fused ramet areas, of strikingly zooid-depauperate tunic. The motility of adult colonial tunicates in general, and didemnid ascidians in particular (Birkel, Cheng & Lewin, 1981), have been mentioned in earlier studies with some reports associated with physical and biological interactions (Carlisle, 1961; Van Thinh, Griffiths & Ngan, 1981). It is suggested the direction of the movement of the ascidian colonies may be dictated by ecological drivers such as the competition for space (Birkel, Cheng & Lewin, 1981; Van Thinh, Griffiths & Ngan, 1981; Cowan, 1981; Ryland, Wigley & Muirhead, 1984; Bak, Sybesma & Duyl, 1981). In this study it appears that, while not rigorously tested, *D. vexillum* movements away from fusion zones tend to be generally ‘upwards’, perhaps indicating that such colonies can respond to gravitational direction.

The observations of *D. vexillum* transient colony fusions reported here provoke the question of if frequent chimerism, even transient chimerism, coupled with highly mobile behavior, is a previously unrecognized, but possibly commonplace, phenomenon amongst colonial marine invertebrates. Indeed, until quite recently it went largely unremarked that a significant number of highly invasive marine invertebrate organisms, including colonial tunicates (Lambert, 2007; Lambert, 2002), form chimeras in nature. The literature further attests to multiple examples of invasive populations of colonial tunicate species having high percentages of chimeric colonies, many of which also display dynamically changing colony structures. Fusions have been reported in other didemnid ascidians such as *Diplosoma listerianum* (Bishop & Sommerfeldt, 1999; Lambert, 2002), *Didemnum fulgens* (López-Legentil et al., 2013), in *Perophora japonica* (Pérez-Portela, Turon & Bishop, 2012; Perophoridae) and in botryllid ascidians, such as *Botrylloides violaceus* Westerman, Dijkstra & Harris, 2009), *Botrylloides nigrum/Botrylloides leachii* (Sheets et al., 2016) and *Botryllus schlosseri* (Ben-Shlomo, Douek & Rinkevich, 2001). Aside from the role chimerism may play in biological invasiveness there is also the persistent broader question of what direct individual fitness advantage accrues from fusions between colonies of differing genotypes, an issue that has been thoroughly discussed in the literature (Rinkevich, 2004; Rinkevich & Yankelevich, 2004; Rinkevich, 2011; Rinkevich & Weissman, 1992; Rinkevich & Weissman, 1987; Rinkevich, 1996; Buss, 1982; Grosberg & Quinn, 1986; Rinkevich, 2002). We speculate that restricted fusion times, as reported here and in other cases (Bishop & Sommerfeldt, 1999; Santelices et al., 2017), may have evolved to reduce the inclusive fitness costs of long term competitive interactions between cell lineages within chimeras while still conferring immediate selective advantages on the two interacting genotypes, perhaps thereby allowing conspecific colonies to fast colonizing new substrate areas. Certainly it is clear that the popular ‘individual-focused’ application of the concept of natural selection needs modification when dealing with highly social animals like colonial marine invertebrates.

In summary, we propose that the movement of *D. vexillum* colonies documented in this study, coupled with transient allogeneic inter-colony fusions, play a significant role in the bioinvasive success of *D. vexillum.* Such movements (Sellers, Fagerberg & Litvaitis, 2013; Lambert, 2002; López-Legentil et al., 2013; Pérez-Portela, Turon & Bishop, 2012; Westerman, Dijkstra & Harris, 2009; Sheets et al., 2016) represent a clearly definable phenotypic trait that can be evoked to explain the striking success of *D. vexillum* both in establishing in new locations and then rapidly spreading from initial establishment sites. This study also re-emphasizes a more general need to consider social interactions when trying to understand bioinvasive processes (Smith et al., 2012; Van Wilgenburg, Torres & Tsutsui, 2010). Finally, as with other colonial ascidians, *D. vexillum* is presented with the trade-offs inherent in chimeric colony formation. On the one hand the increased genetic heterogeneity within such chimeras is likely to be beneficial to the colony overall (i.e., ameliorated fitness in harsh environments; (Rinkevich, 2004; Rinkevich, 2011)) but accompanying such benefits is the risk of somatic/germ cell competition and parasitism, a risk that may be limited by transient chimerism. It is further suggested that colony mobility associated with inter-colony conflict avoidance behaviours may play a role in the bioinvasiveness of other colonial marine invertebrates and therefore warrants further study.

##  Supplemental Information

10.7717/peerj.5006/supp-1 Figure S1 Photographic images of *D. vexillum* experimental pairings *Set I* Pairings were established between ramets from four different *D. vexillum* colonies, denoted A–D, in duplicate. Photographic images of the 12 pairwise combinations were taken daily revealing the outcomes of the 6 possible pairwise combinations between the four genotypes. Descriptive summaries of the outcomes of the pairing experiments are given in Table 1. At the end of each pairing the *D. vexillum* colonies were dissected into the segments (denoted by lower-case letters: a, b, c, etc.), with the boundaries indicated by red lines. The dissected sections were genotyped at polymorphic microsatellite loci to determine the relative amounts of the paired ramet genotypes to each dissected region (for the semi-quantitative ratio assessment methodology see materials and methods). The cotton threads that held the *D. vexillum* subclones to the slides were not removed until termination of the experiment and so provide landmarks for the positioning of the ramets. Abbreviations: reverse, side of glass slide opposite to that on which the pairing was established; nd, not done.

10.7717/peerj.5006/supp-2 Figure S2 Images of the *Set II* experimental pairing of ramets *D. vexillum* H x I Representative images over 12 days and separated by 1 day, were extracted from the time-lapse film of the ramet H x I pairing (supplementary materials movie-S1). The images show an initial fusion of the Hand I ramets subsequently followed by separation movements.

10.7717/peerj.5006/supp-3 Figure S3 Images of the *Set II* experimental pairing of ramets *D. vexillum* H x L Representative images over 10 days and separated by 1 day, were extracted from the time-lapse film of the ramet H x L pairing (supplementary materials movie-S2). The images show a lack of fusion of the H and L ramets that was followed by separation movements.

10.7717/peerj.5006/supp-4 Supplemental Information 1 Table S1 and S2. Details of the polymorphic microsatellite loci used to genotype D. vexillum ramets 1 in this study

10.7717/peerj.5006/supp-5 Movie S1 Time lapse movie of the *Set II* experimental pairing of *D. vexillum* ramets H x I The movie is a composite of single-frame images taken at 5 minute intervals over 12 days (see materials and methods for further details).

10.7717/peerj.5006/supp-6 Movie S2 Time lapse movie of the *Set II* experimental pairing of *D. vexillum* ramets H x L The movie is a composite of single-frame images taken at 5 minute intervals over 10 days (see materials and methods for further details).

10.7717/peerj.5006/supp-7 Supplemental Information 2 Fidler et al. associated GenBank entries Sequences corresponding to GenBank entries KU167099 and KU167100.

## Additional Information and Declarations

### DNA Deposition

The following information was supplied regarding the deposition of DNA sequences: Two new microsatellite sequences described here are accessible via GenBank accession numbers: KU167099 and KU167100.

### Data Availability

The following information was supplied regarding data availability: The raw data are provided in the Supplemental Files.
