# Supplementary material for: Efficient dispersal and substrate acquisition traits in a marine invasive species via transient chimerism and colony mobility
Source: PeerJ. 2018 Jun 13;6:e5006. doi: 10.7717/peerj.5006 (PMC6004106; doi:10.7717/peerj.5006)

Pairing A x B – 1

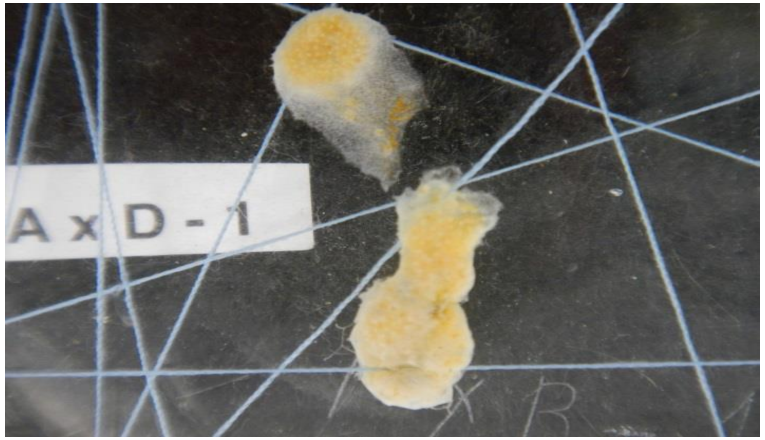

AxB-1: 03-10-2013

(A x D label is an error)

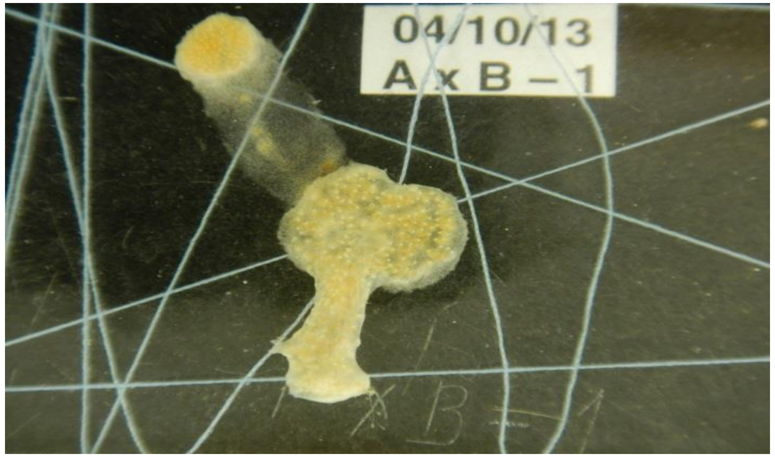

AxB-1: 04-10-2013

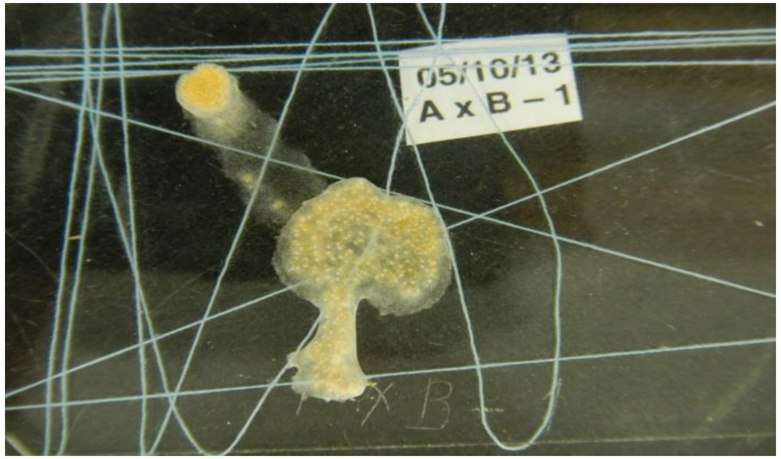

AxB-1: 06-10-2013

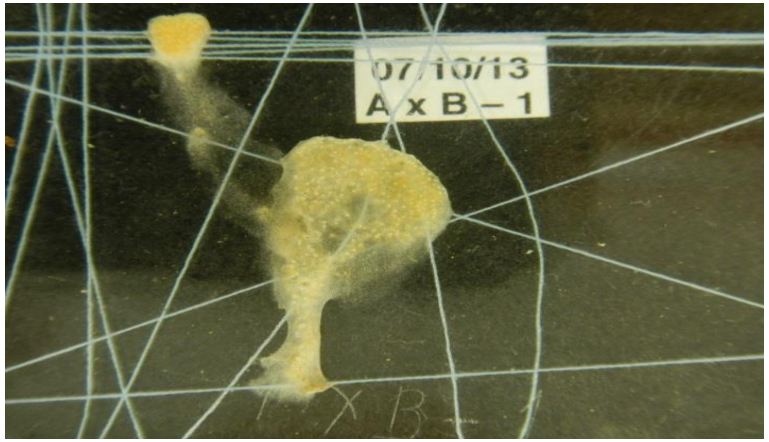

AxB-1: 07-10-2013

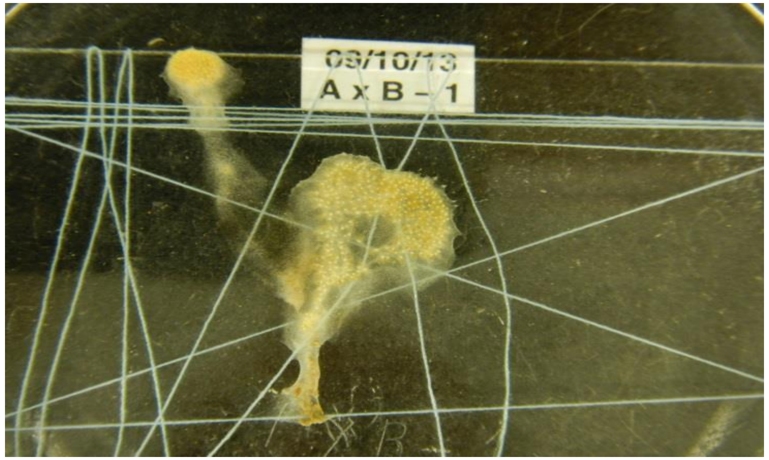

AxB-1: 08-10-2013

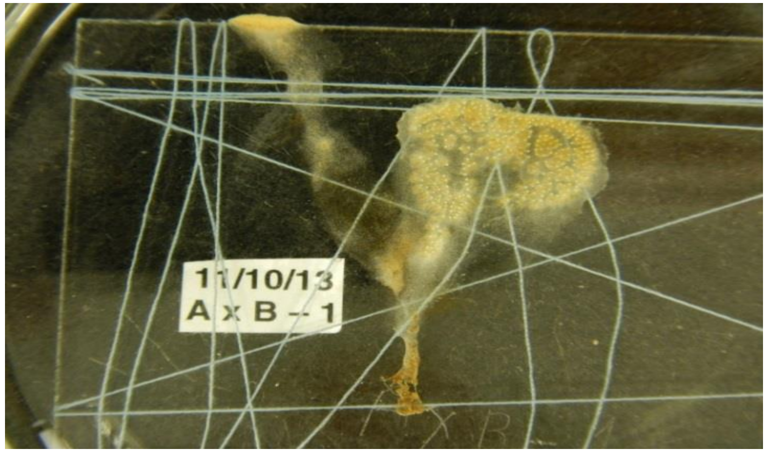

AxB-1: 11-10-2013

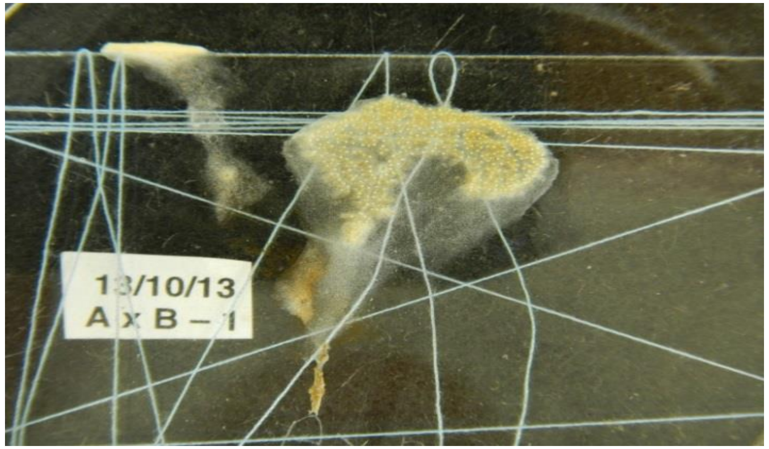

AxB-1: 13-10-2013

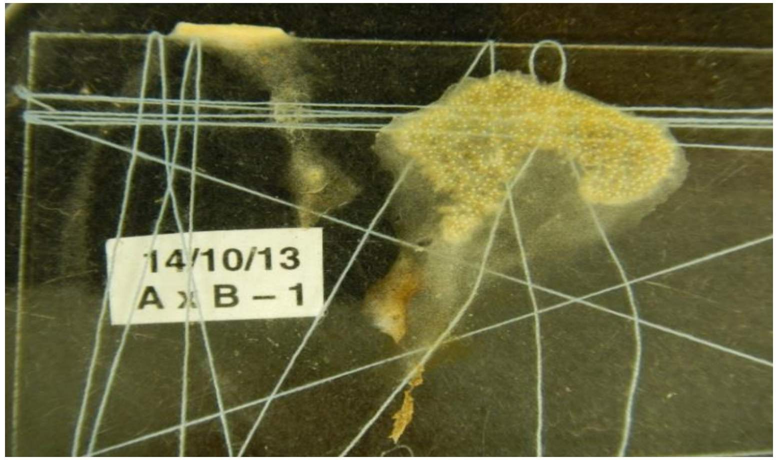

AxB-1: 14-10-2013

AxB-1: 11 day pairing genotyping schematic

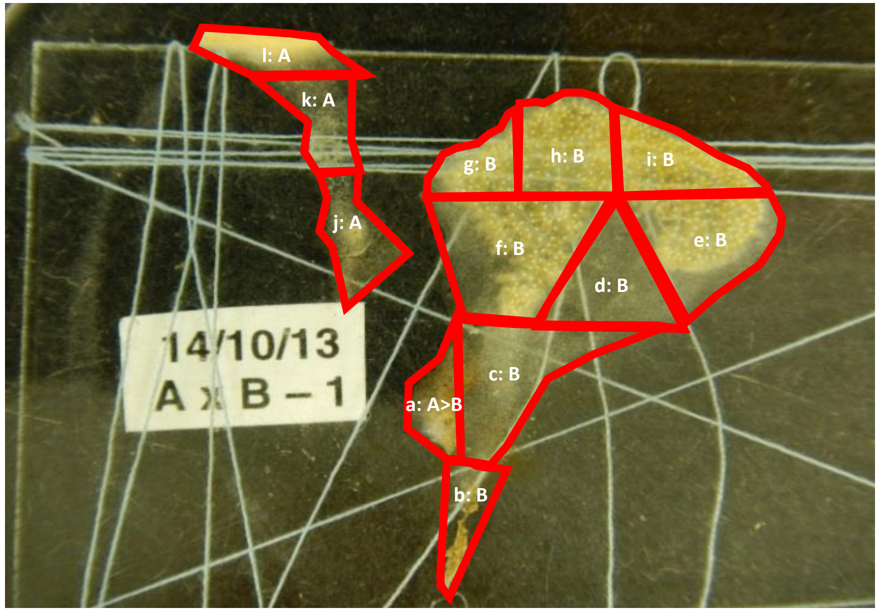

Pairing A x B – 2

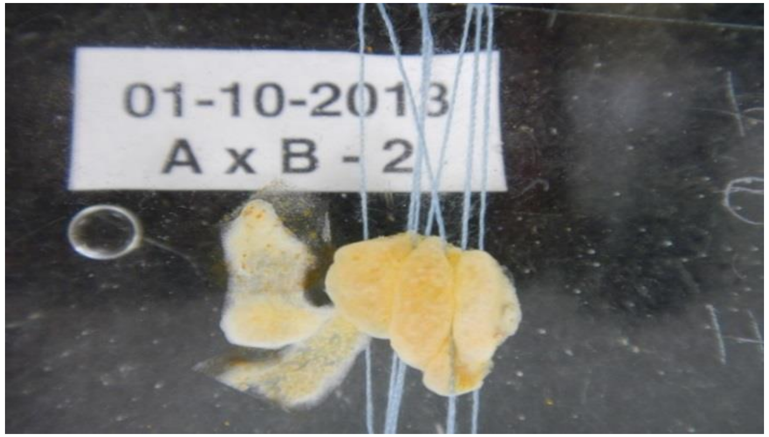

AxB-2: 01-10-2013

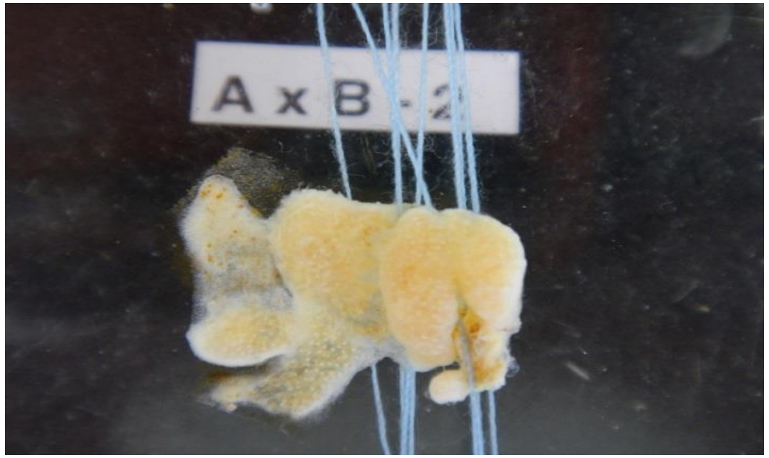

AxB-2: 02-10-2013

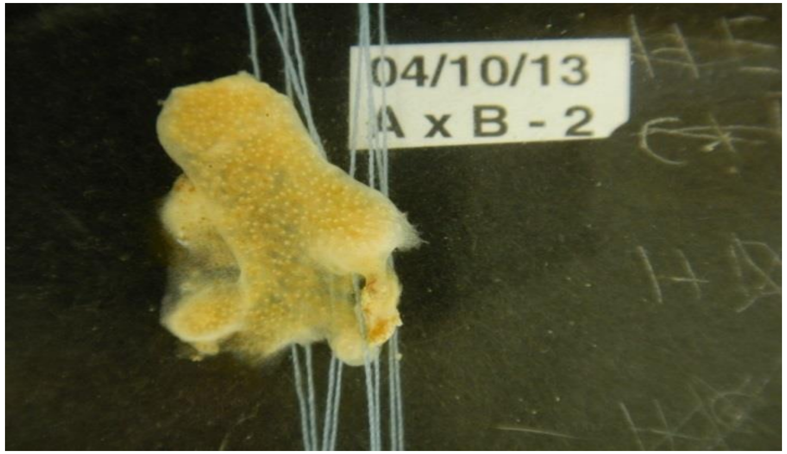

AxB-2: 04-10-2013

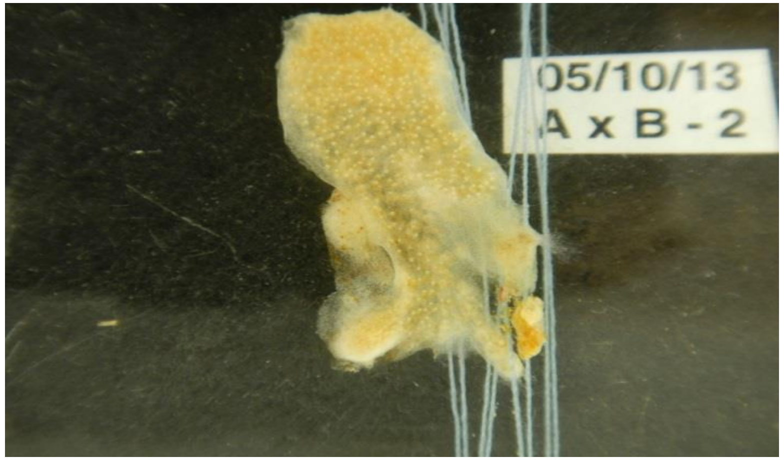

AxB-2: 05-10-2013

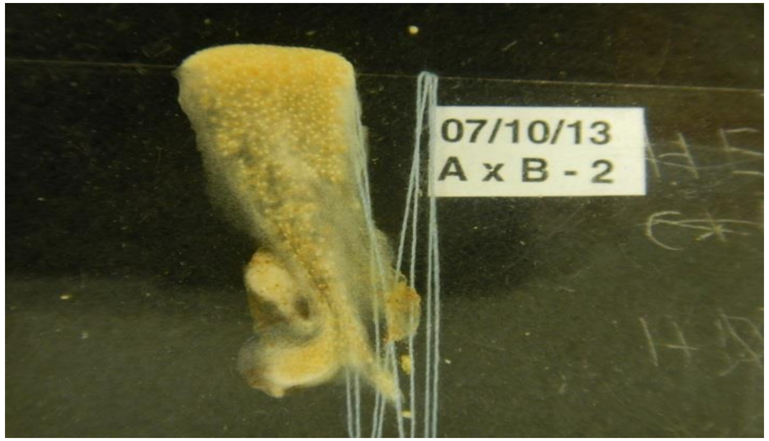

AxB-2: 07-10-2013

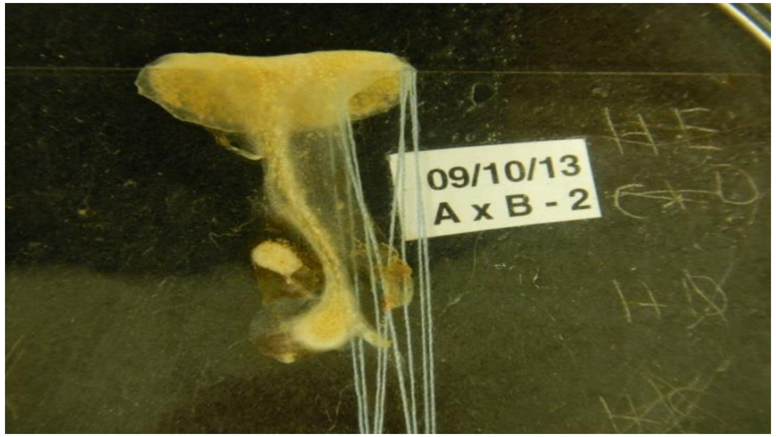

AxB-2: 09-10-2013

AxB-2: 8 day pairing genotyping summary schematic

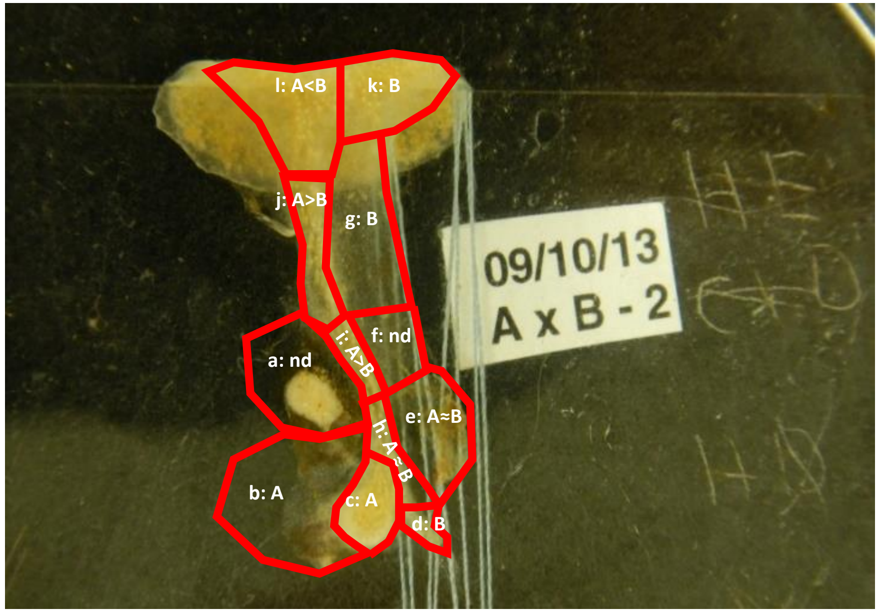

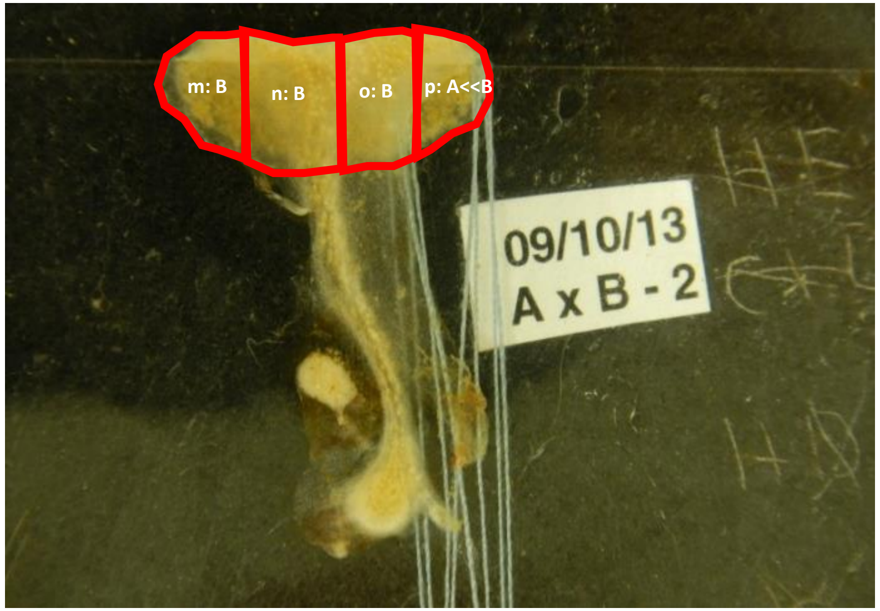

Pairing A x C - 1

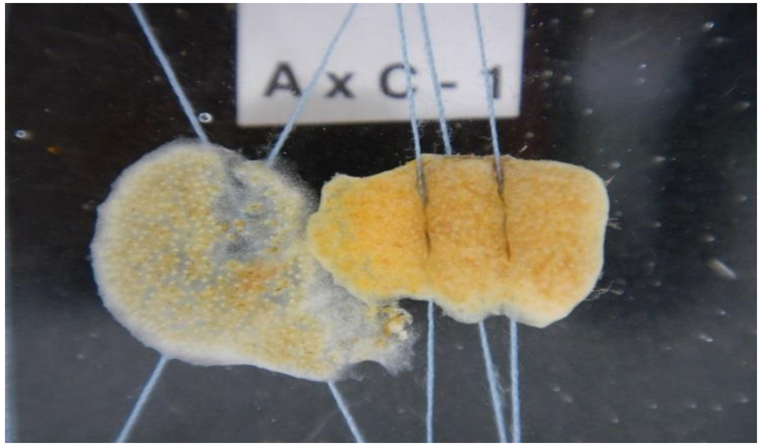

AxC-1: 01-10-2013

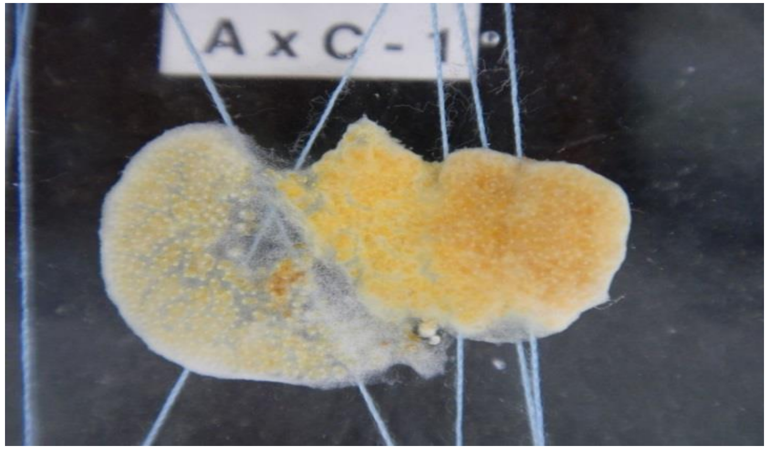

AxC-1: 02-10-2013

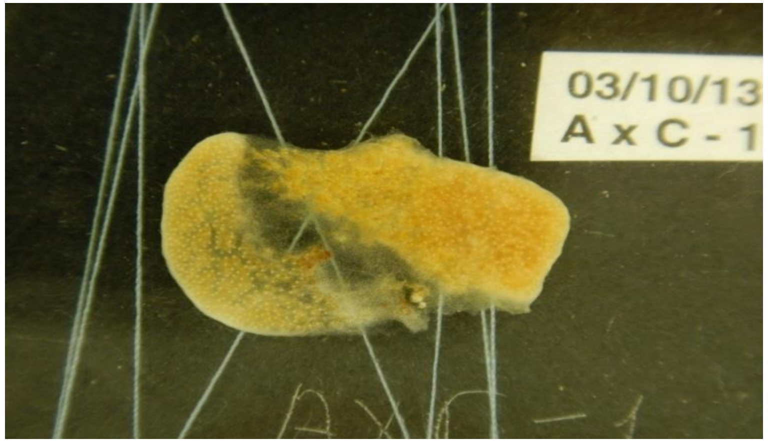

AxC-1: 03-10-2013

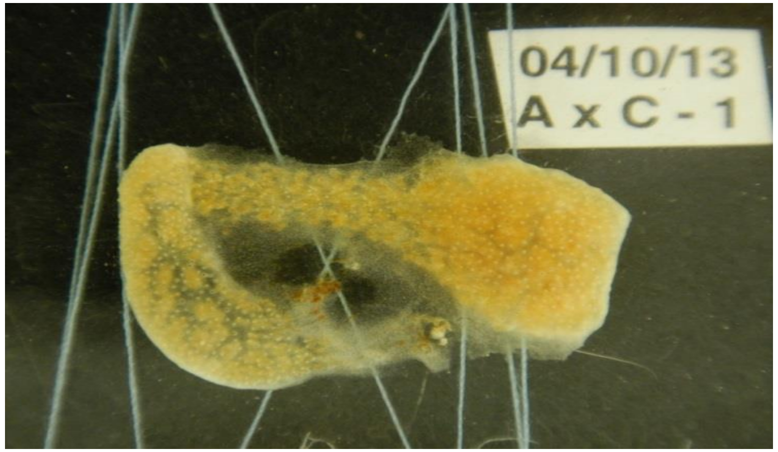

AxC-1: 04-10-2013

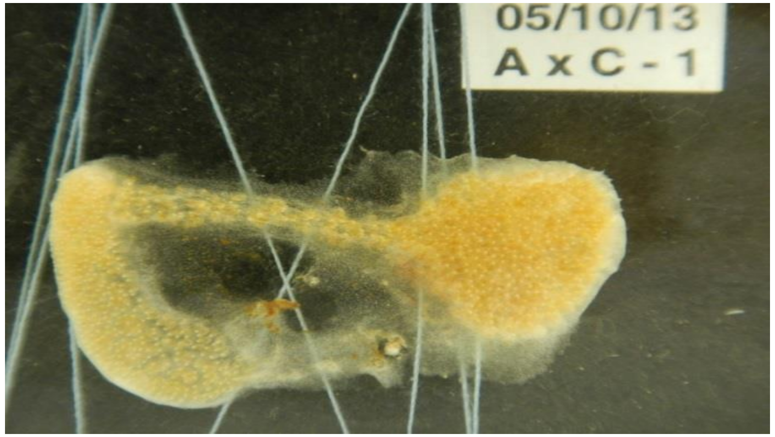

AxC-1: 05-10-2013

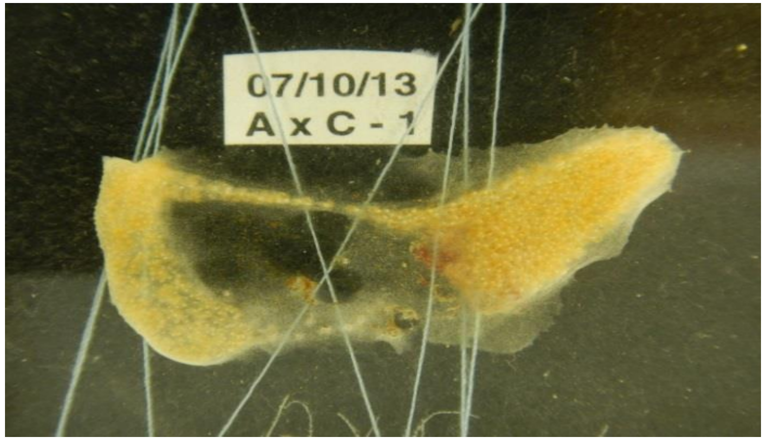

A xC-1: 07-10-2013

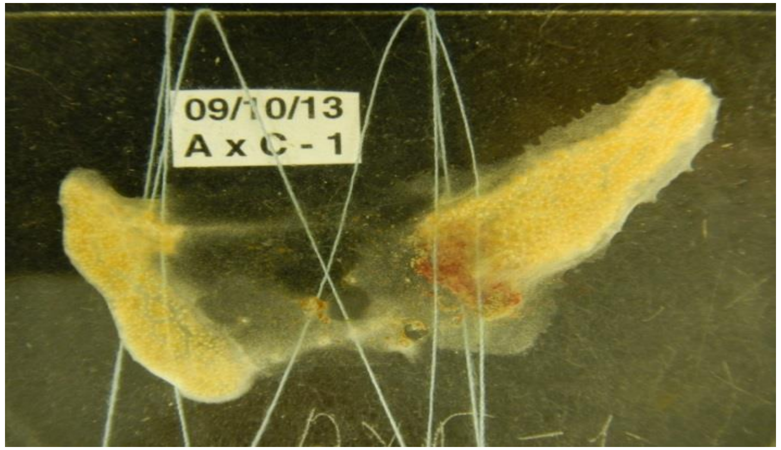

AxC-1: 09-10-2013

AxC-1: 8 day pairing genotyping schematic

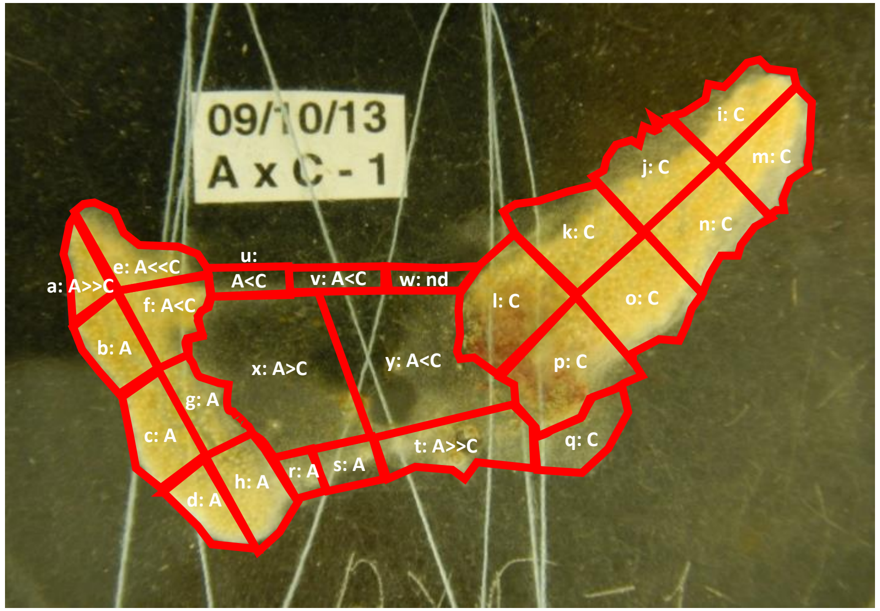

Pairing A x C - 2

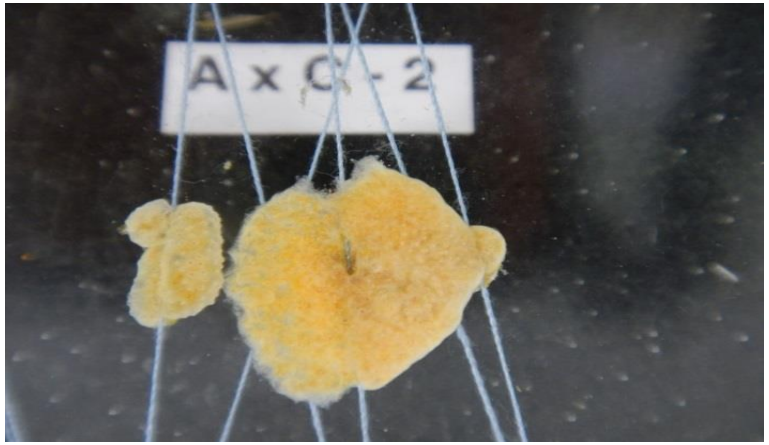

AxC-2: 01-10-2013

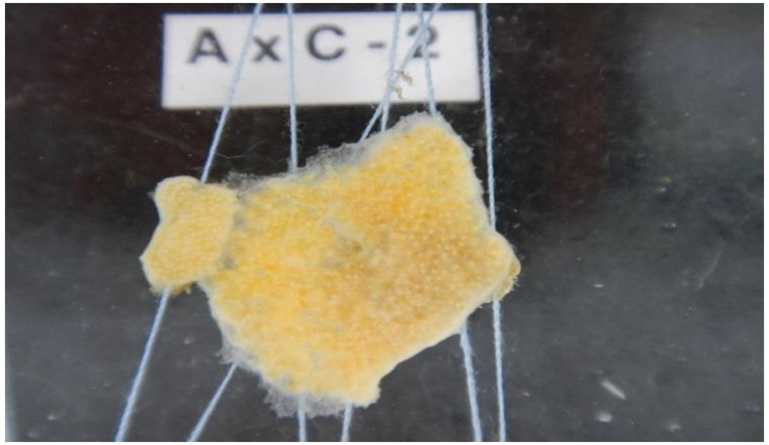

AxC-2: 02-10-2013

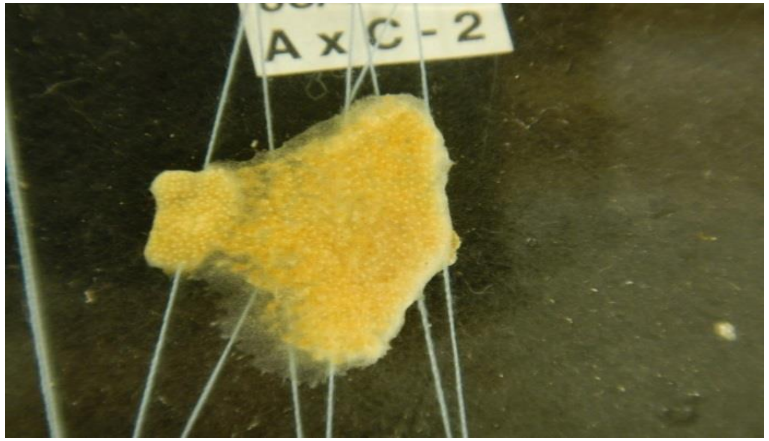

AxC-2: 03-10-2013

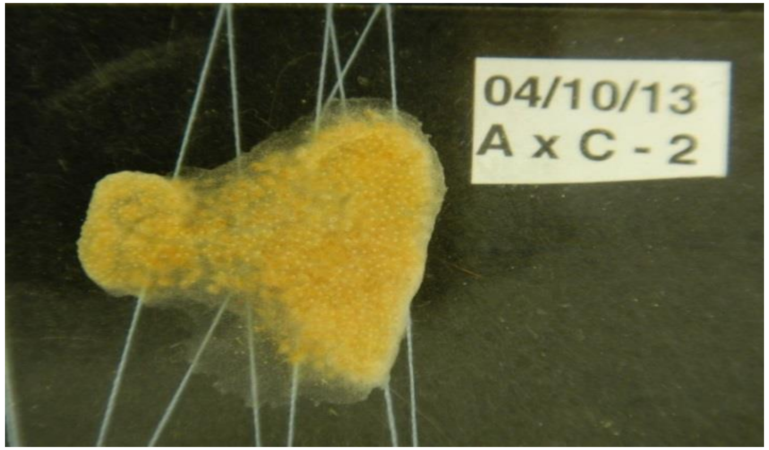

AxC-2: 04-10-2013

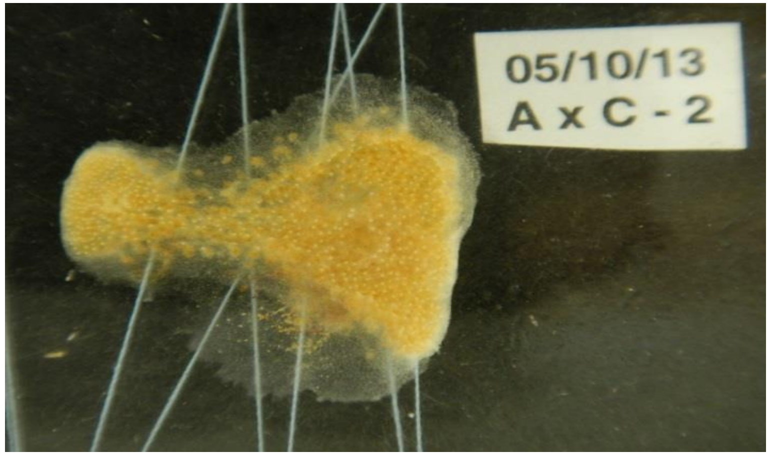

AxC-2: 05-10-2013

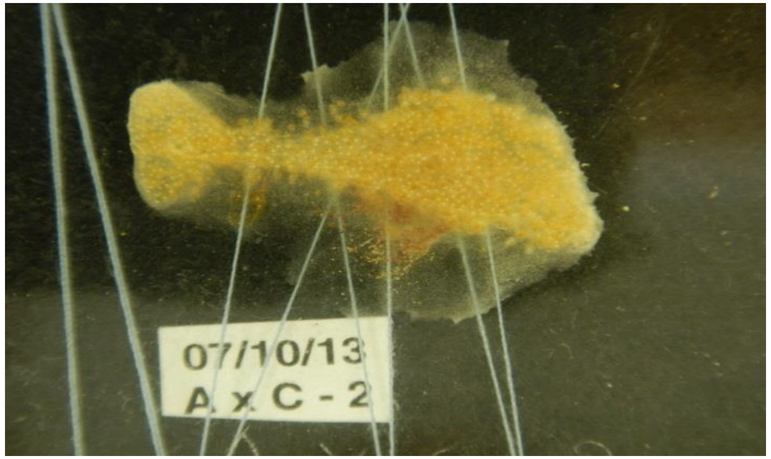

AxC-2: 07-10-2013

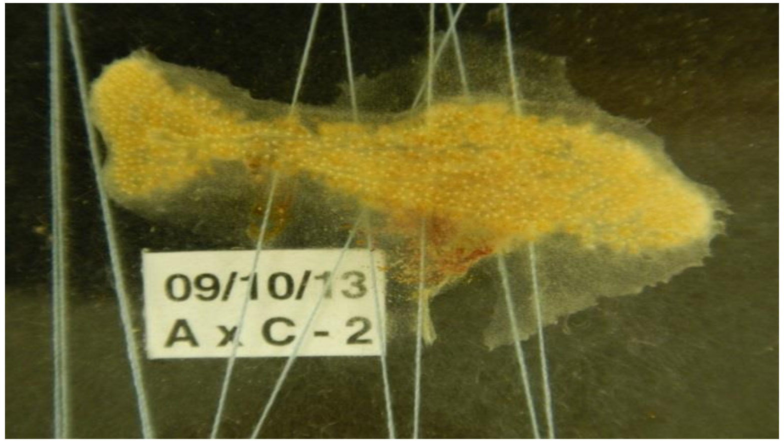

AxC-2: 09-10-2013

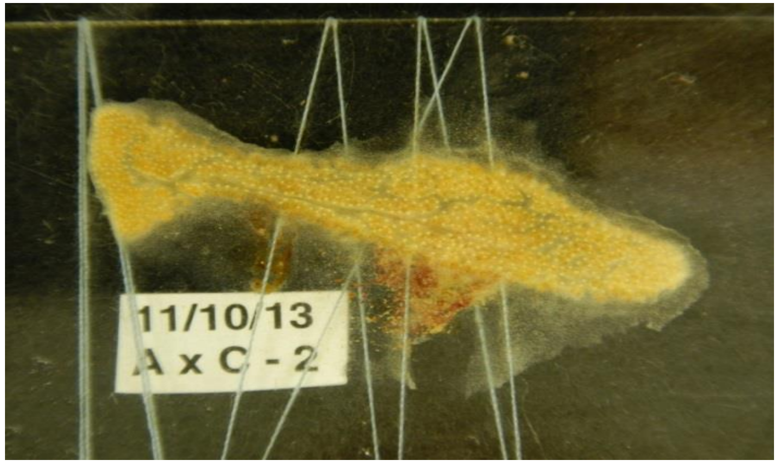

AxC-2: 11-10-2013

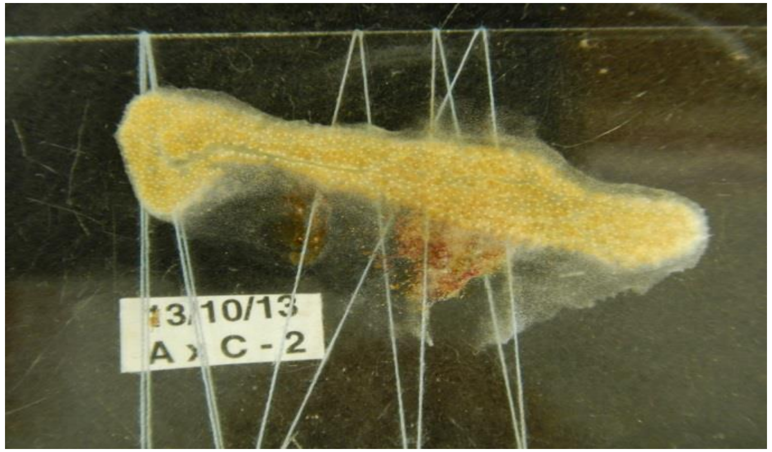

AxC-2: 13-10-2013

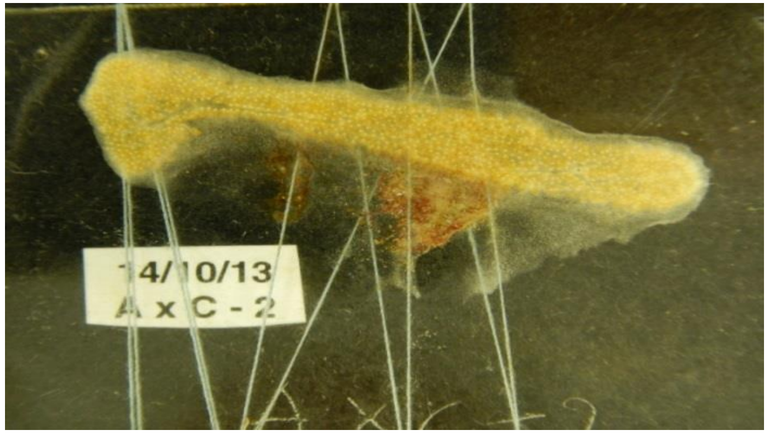

AxC-2: 14-10-2013

AxC-2: 13 day pairing genotyping schematic

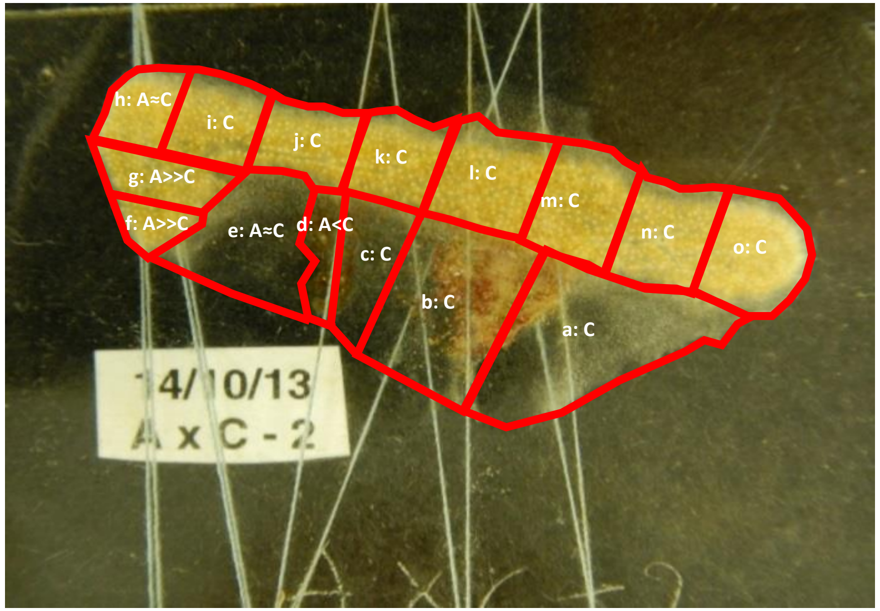

Pairing A x D – 1

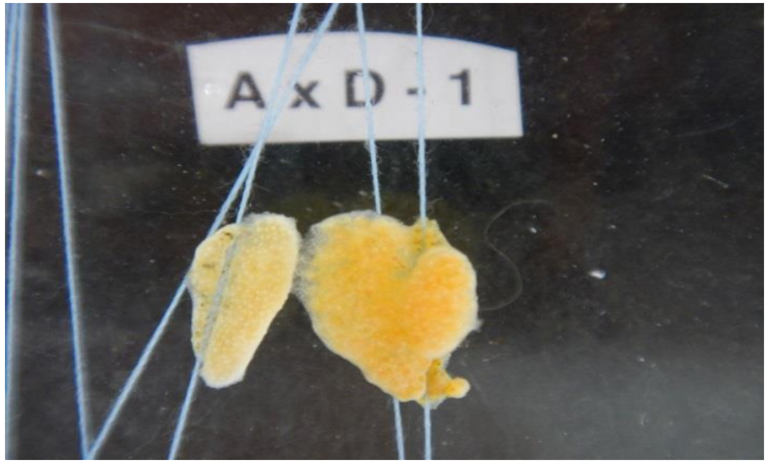

AxD-1: 02-10-2013

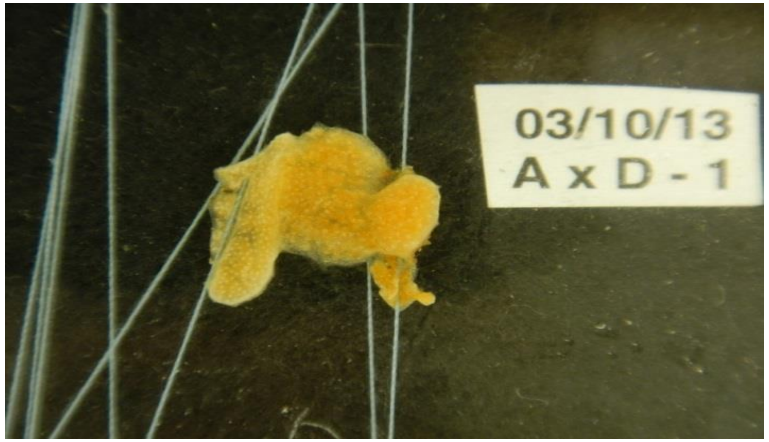

AxD-1: 03-10-2013

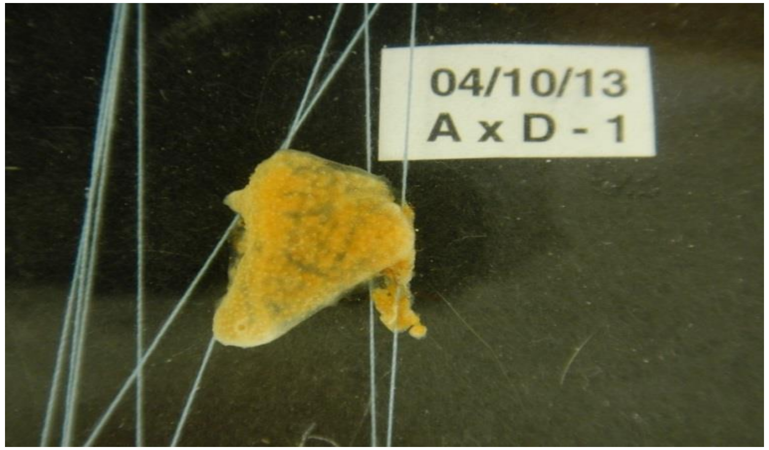

AxD-1: 04-10-2013

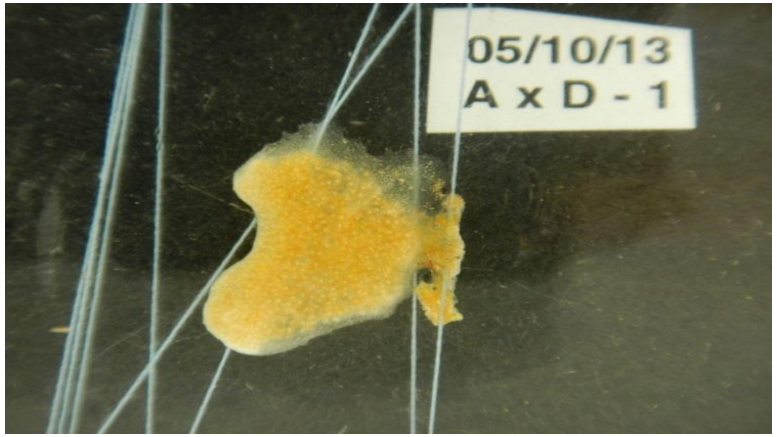

AxD-1: 05-10-2013

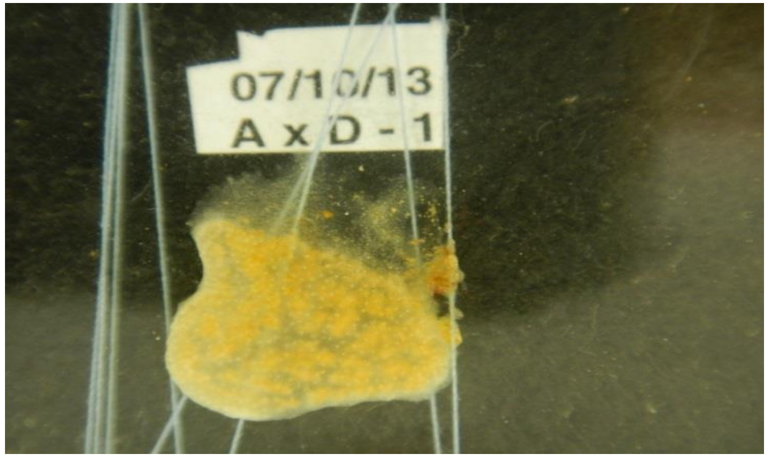

AxD-1: 07-10-2013

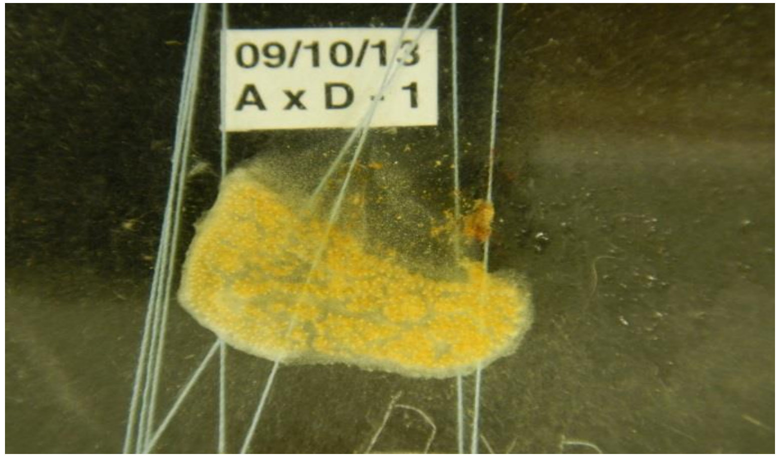

AxD-1: 09-10-2013

AxD-1: 7 day pairing genotyping schematic

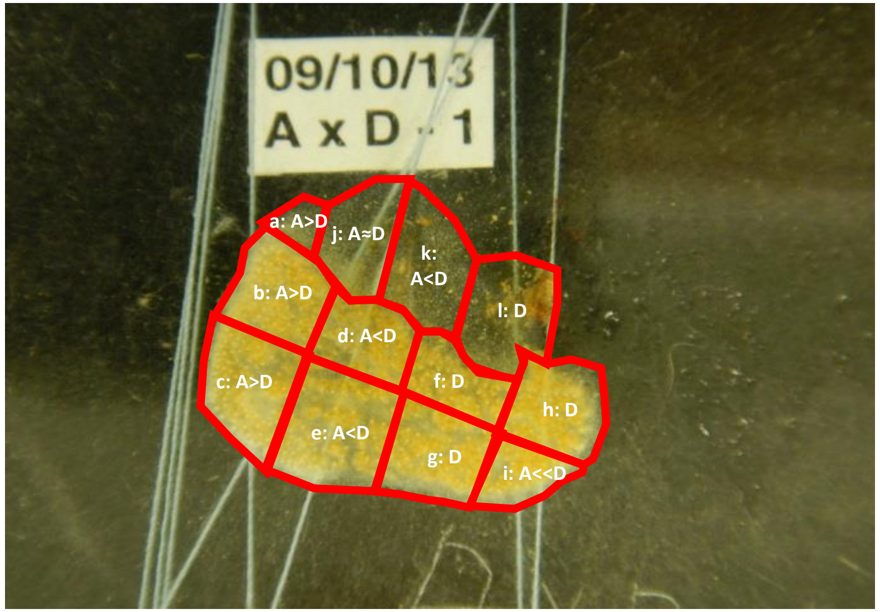

Pairing A x D –2

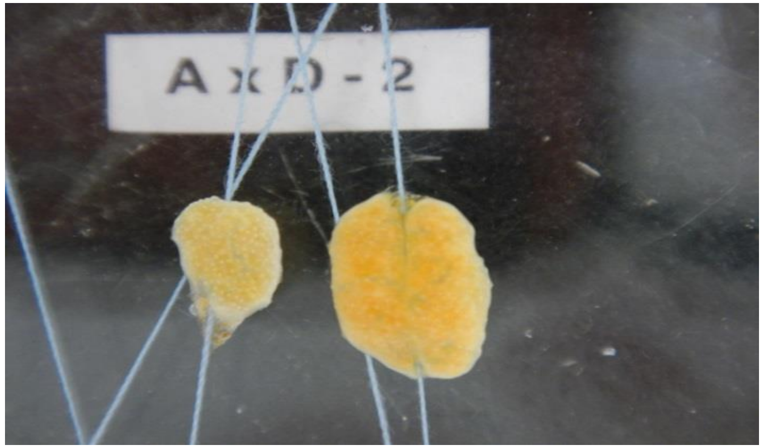

AxD-2: 02-10-2013

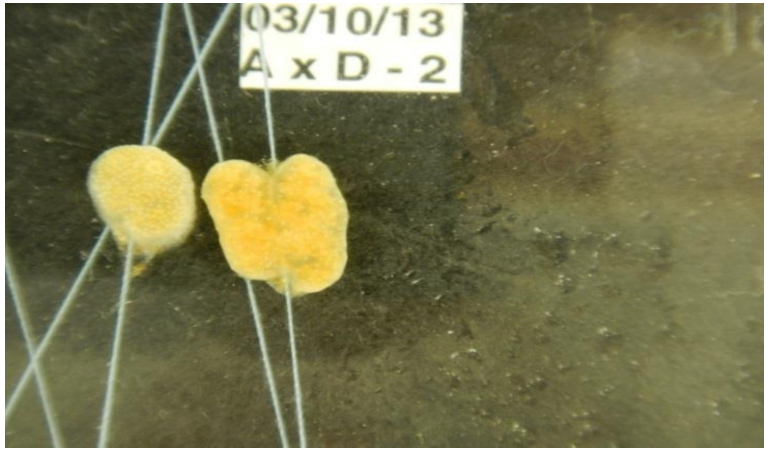

AxD-2: 03-10-2013

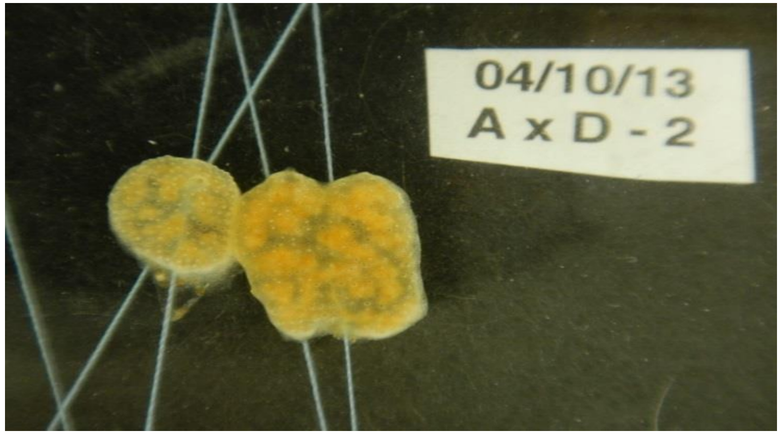

AxD-2: 04-10-2013

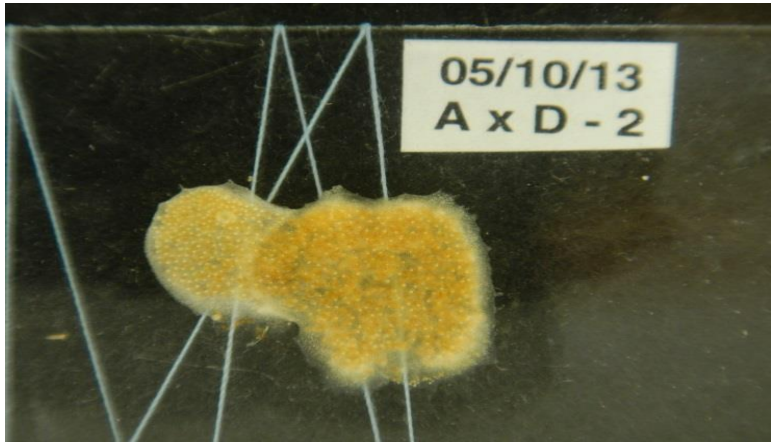

AxD-2: 05-10-2013

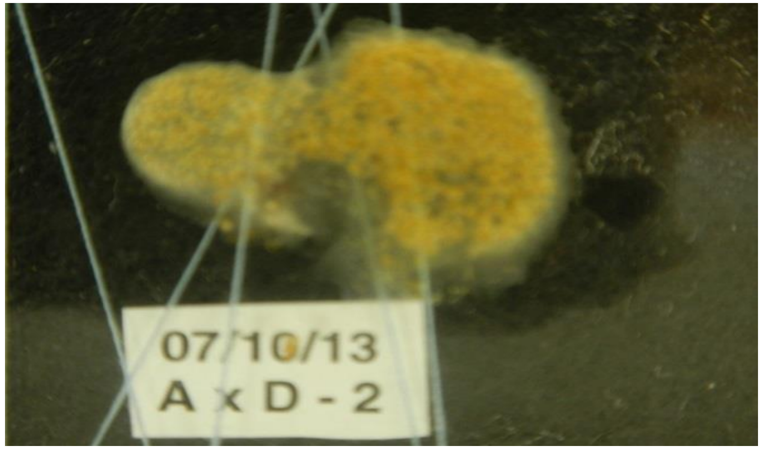

AxD-2: 07-10-2013

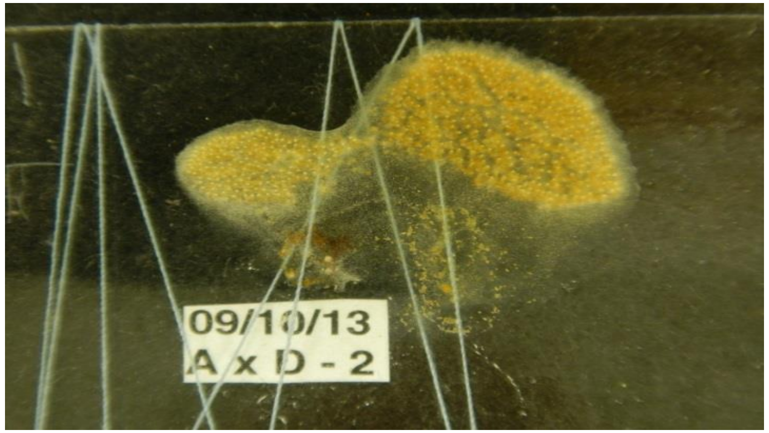

AxD-2: 09-10-2013

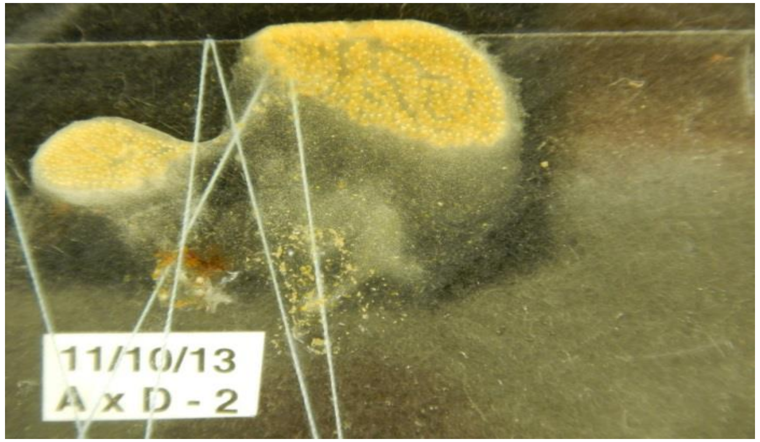

AxD-2: 11-10-2013

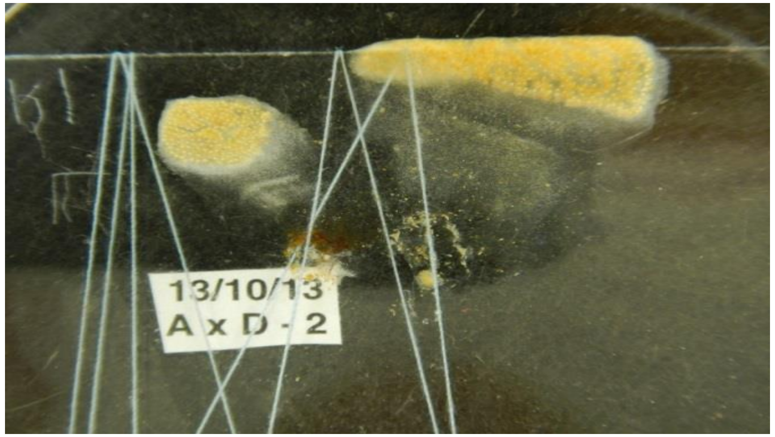

AxD-2: 13-10-2013

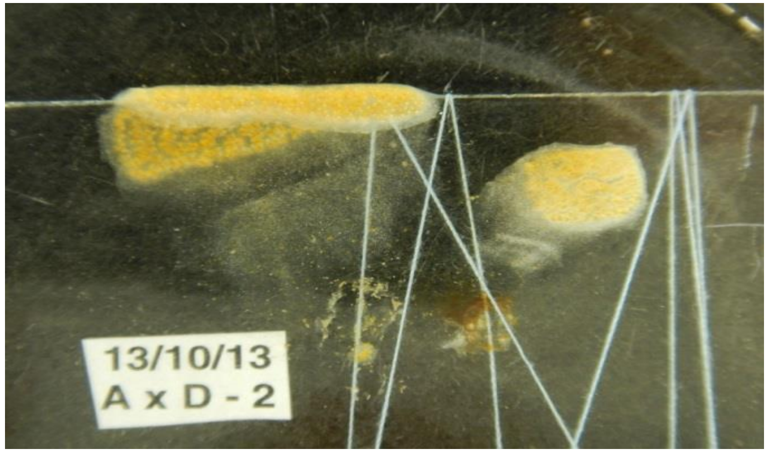

AxD-2: 13-10-2013 (reverse)

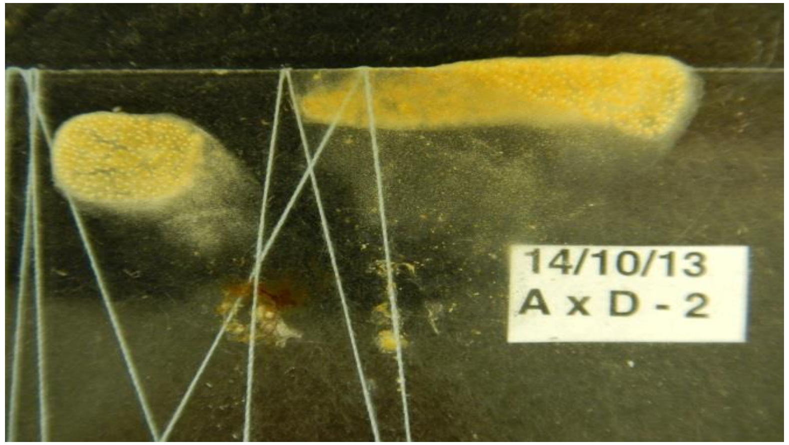

AxD-2: 14-10-2013

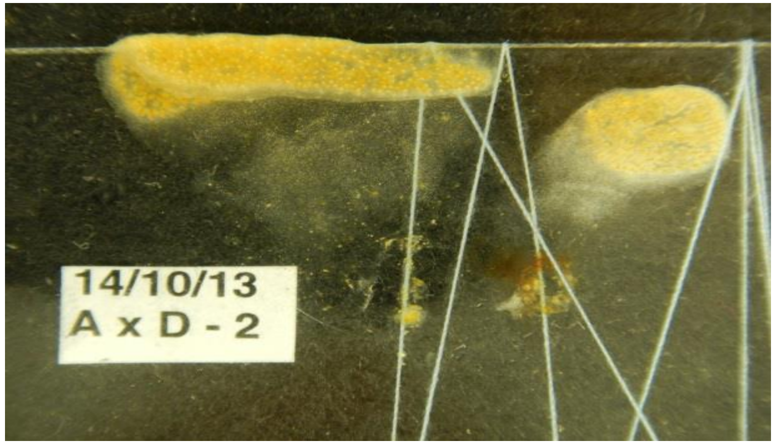

AxD-2: 14-10-2013 (reverse)

AxD-2: 12 day pairing genotyping schematic

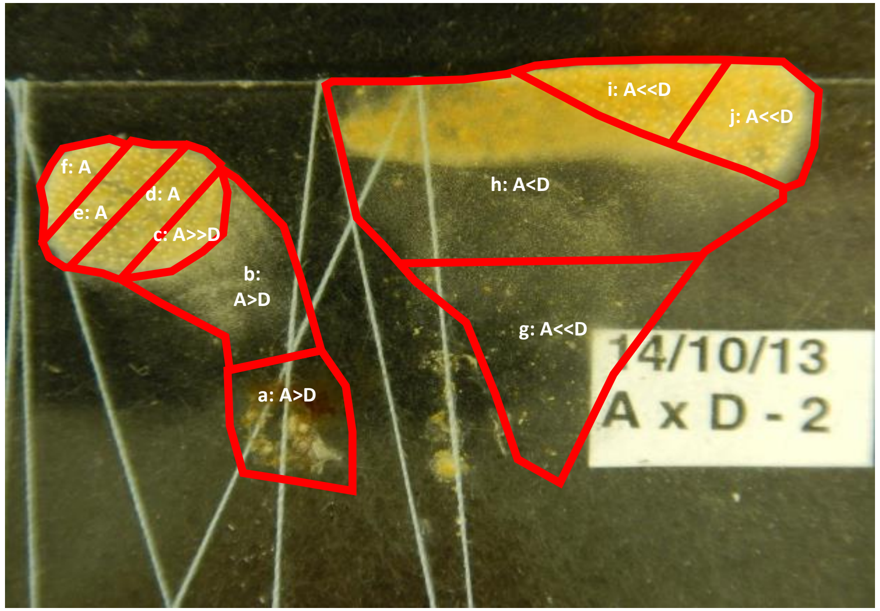

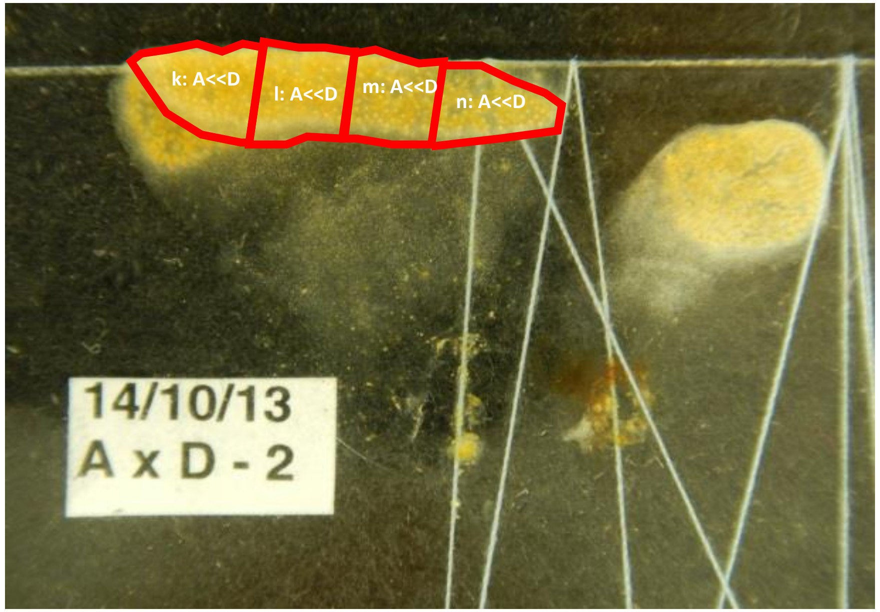

Pairing B x C – 1

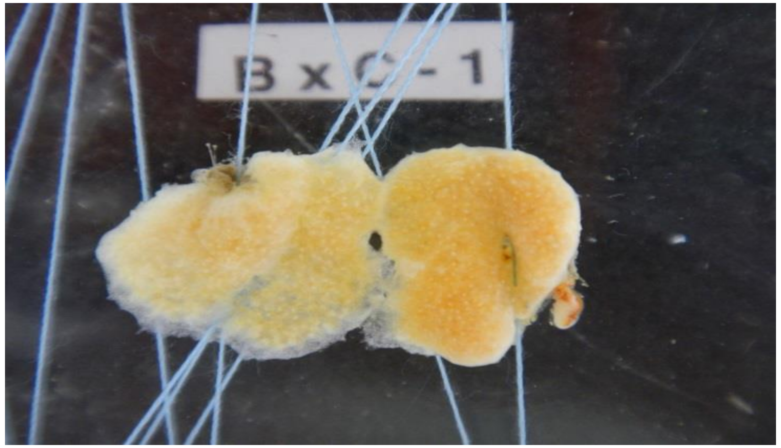

BxC-1: 02-10-2013

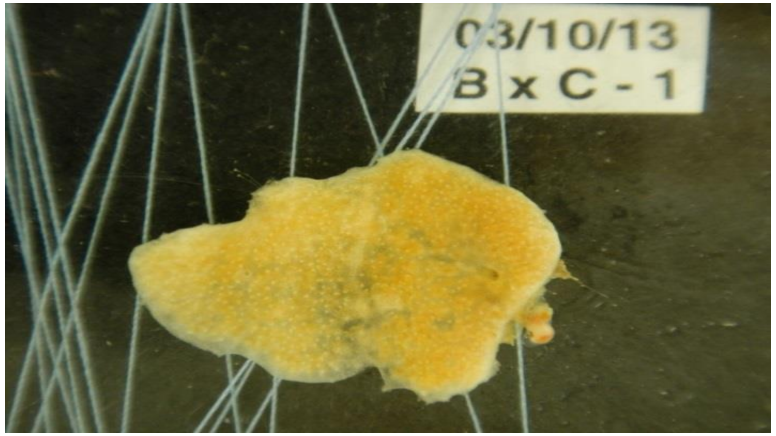

BxC-1: 03-10-2013

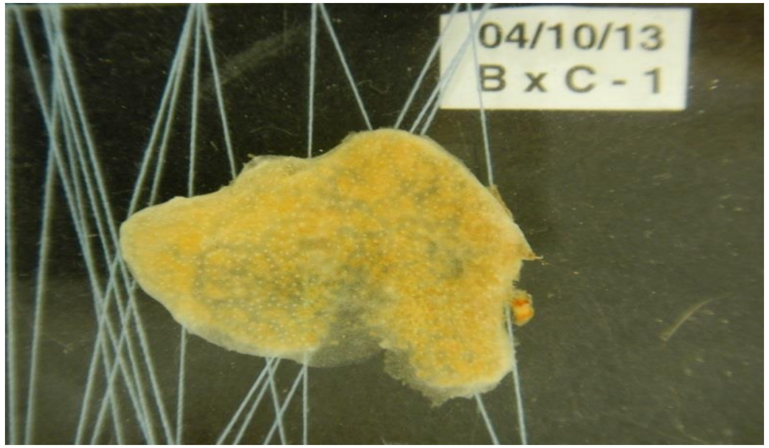

BxC-1: 04-10-2013

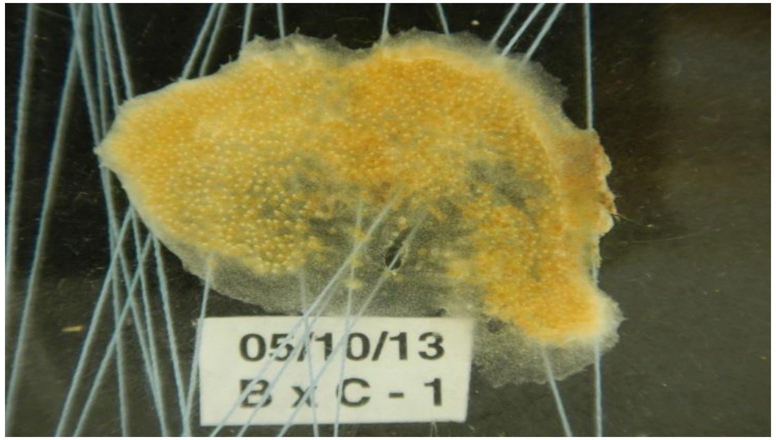

BxC-1: 05-10-2013

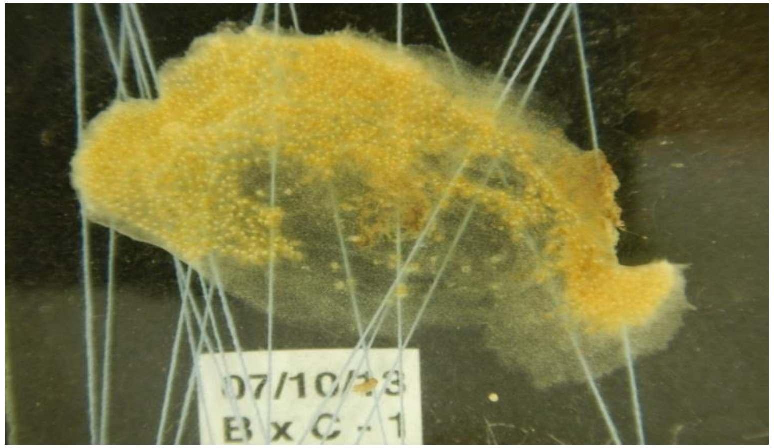

BxC-1: 07-10-2013

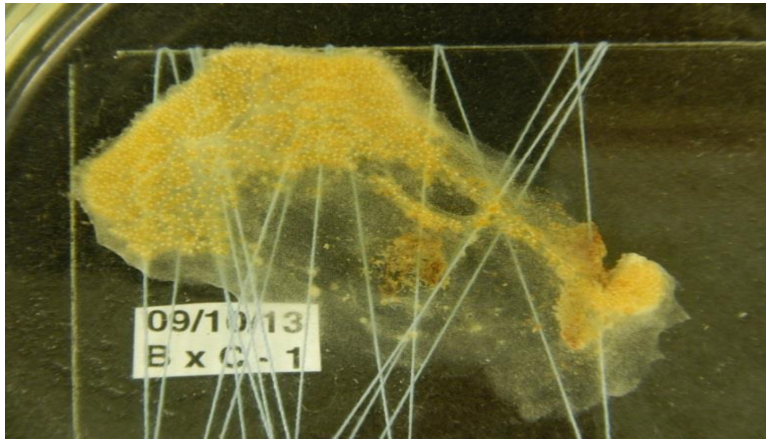

BxC-1: 09-10-2013

BxC-1: 7 day pairing genotyping schematic

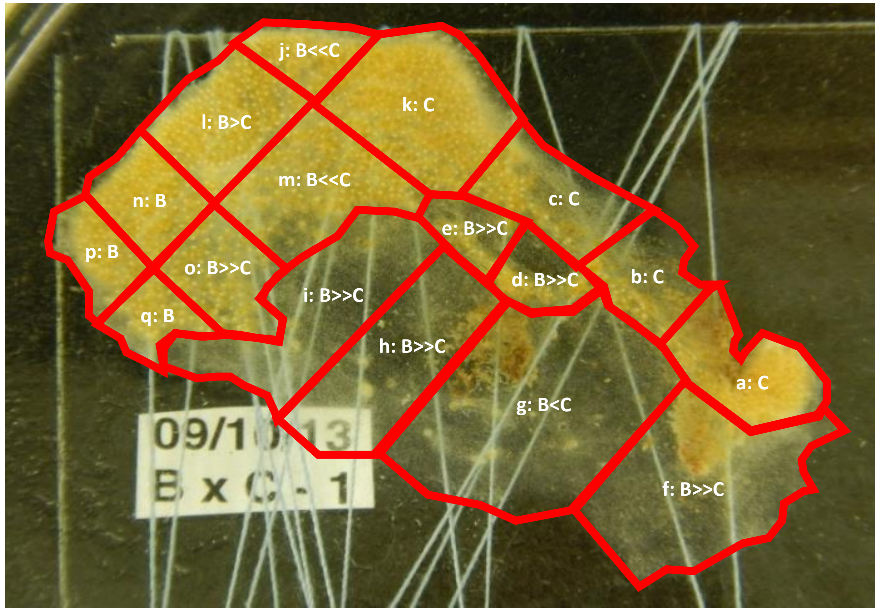

Pairing B x C – 2

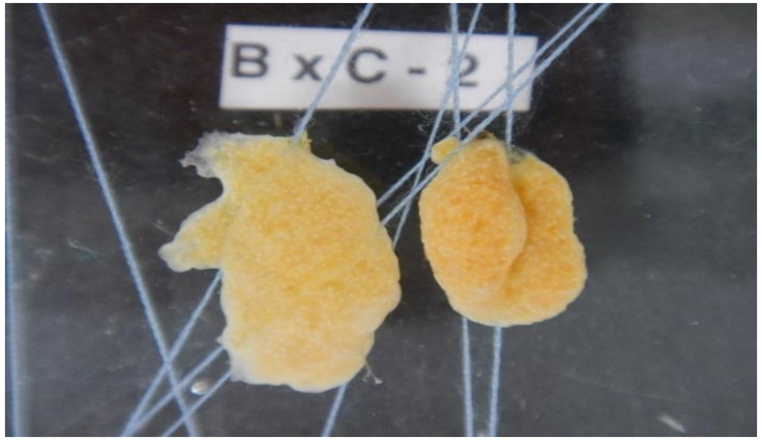

BxC-2: 02-10-2013

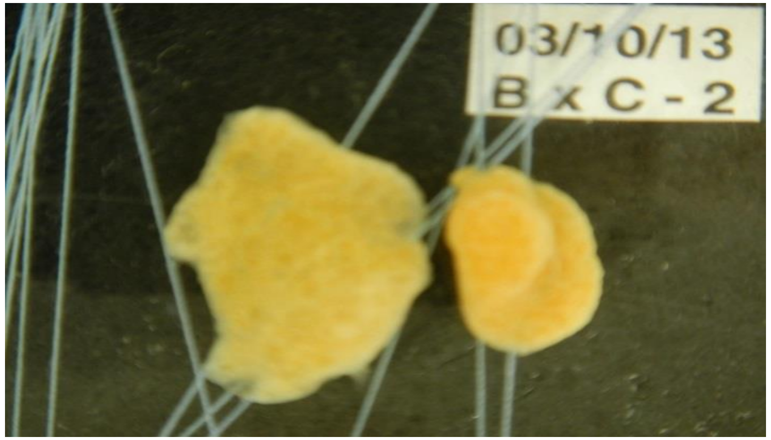

BxC-2: 03-10-2013

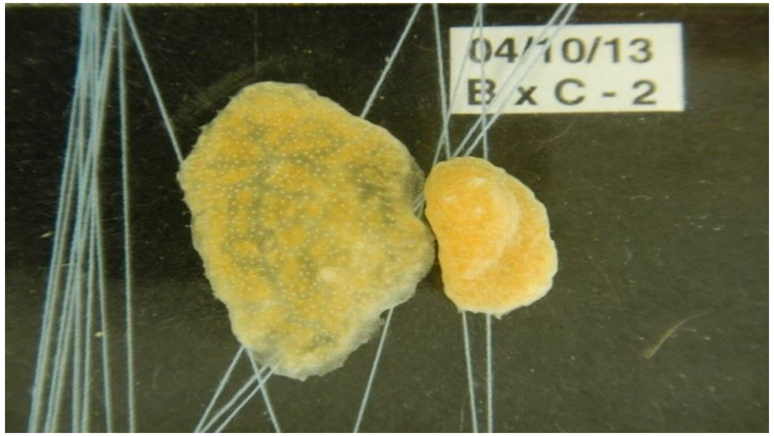

BxC-2: 04-10-2013

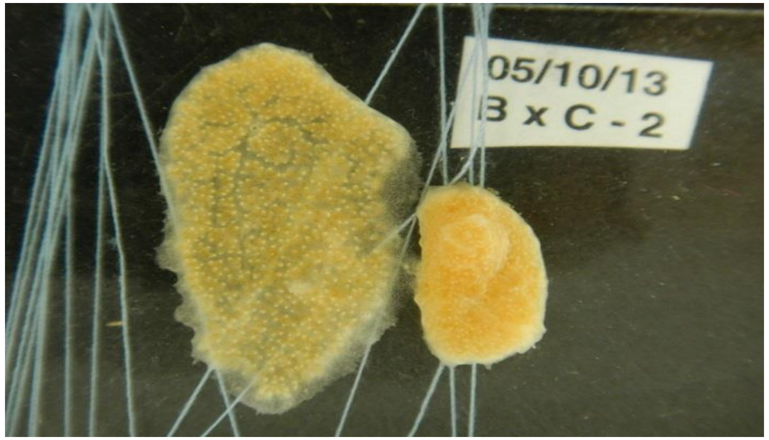

BxC-2: 05-10-2013

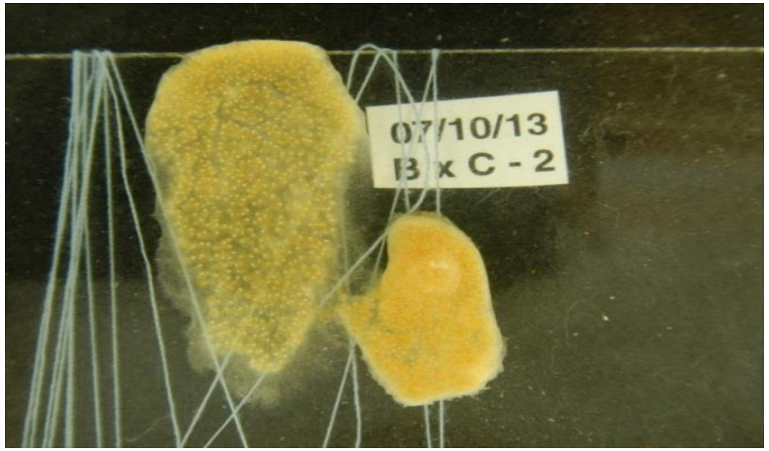

BxC-2: 07-10-2013

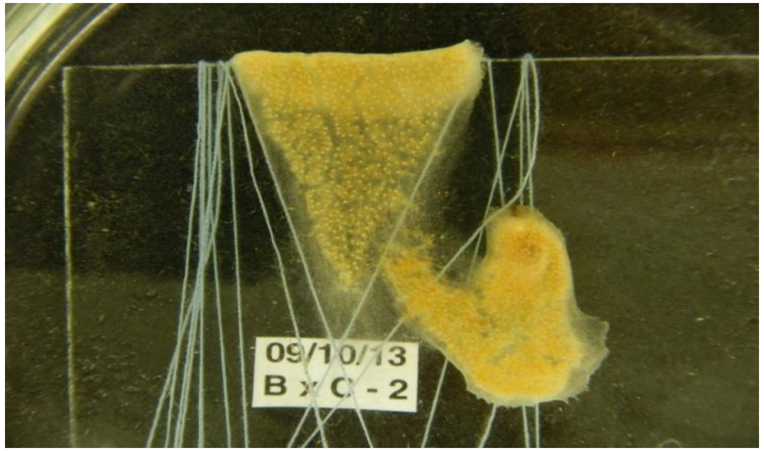

BxC-2: 09-10-2013

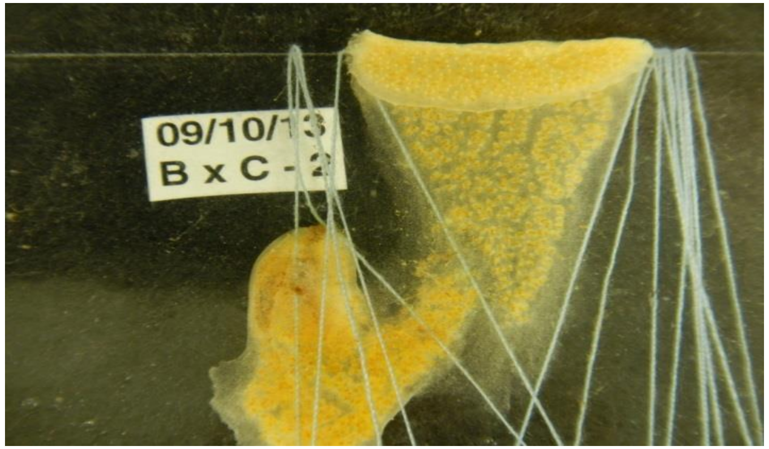

BxC-2: 09-10-2013 (reverse)

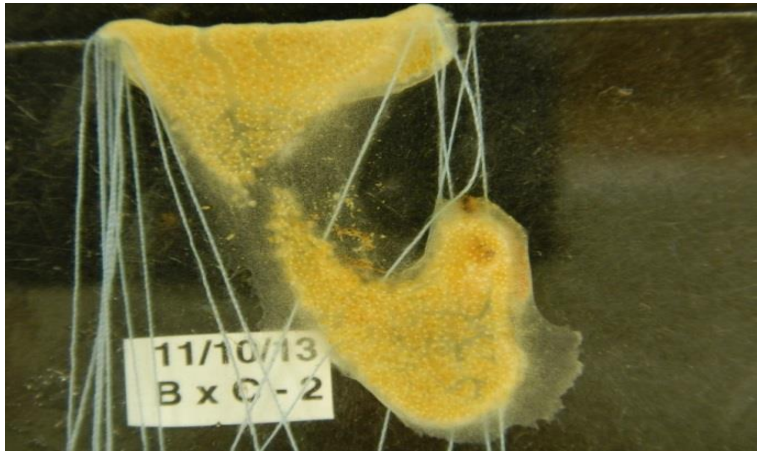

BxC-2: 11-10-2013

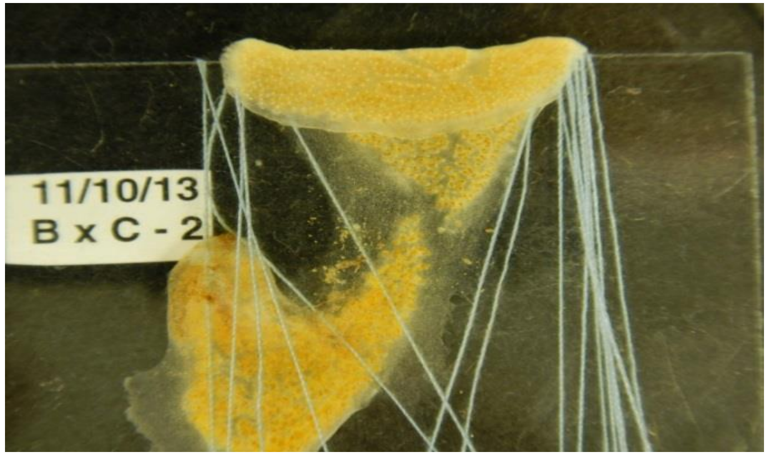

BxC-2: 11-10-2013 (reverse)

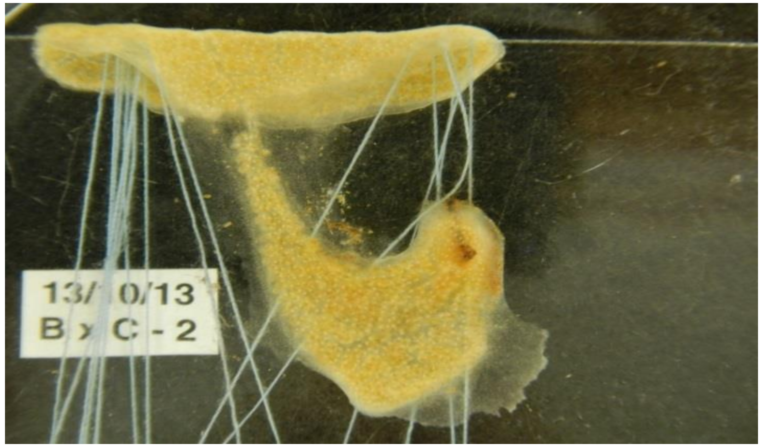

BxC-2: 13-10-2013

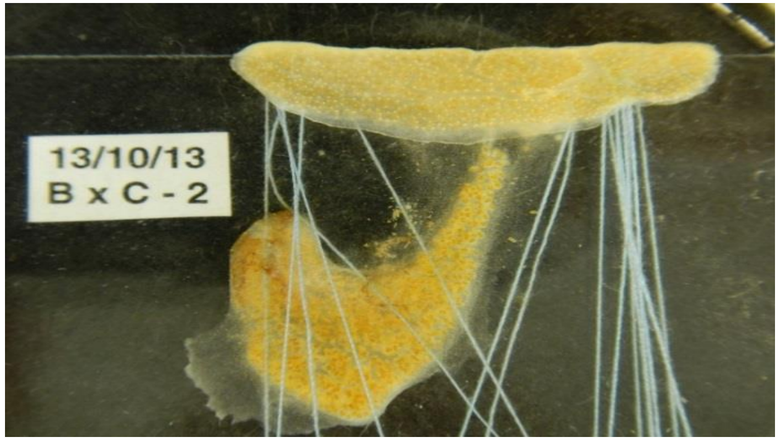

B x C-2: 13-10-2013 (reverse)

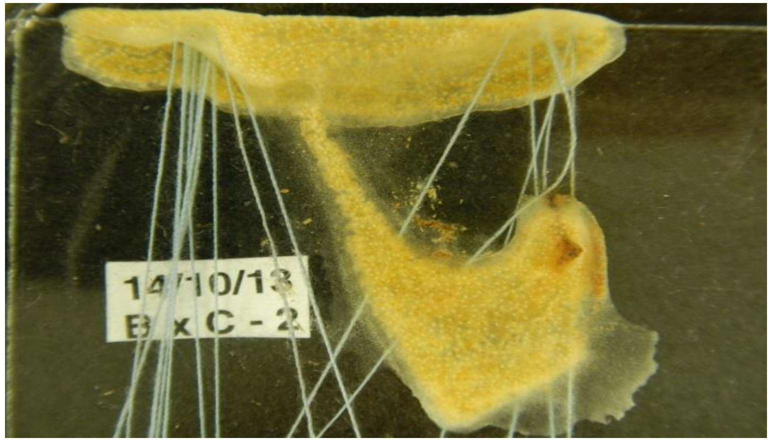

BxC-2: 14-10-2013

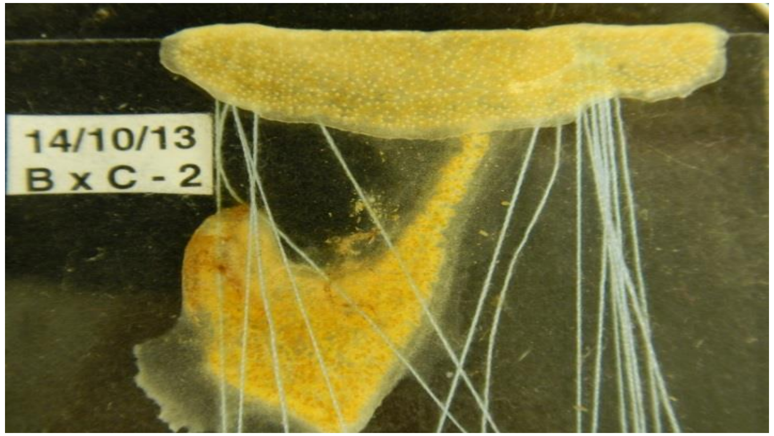

BxC-2: 14-10-2013 (reverse)

BxC-2: 12 day pairing genotyping schematic

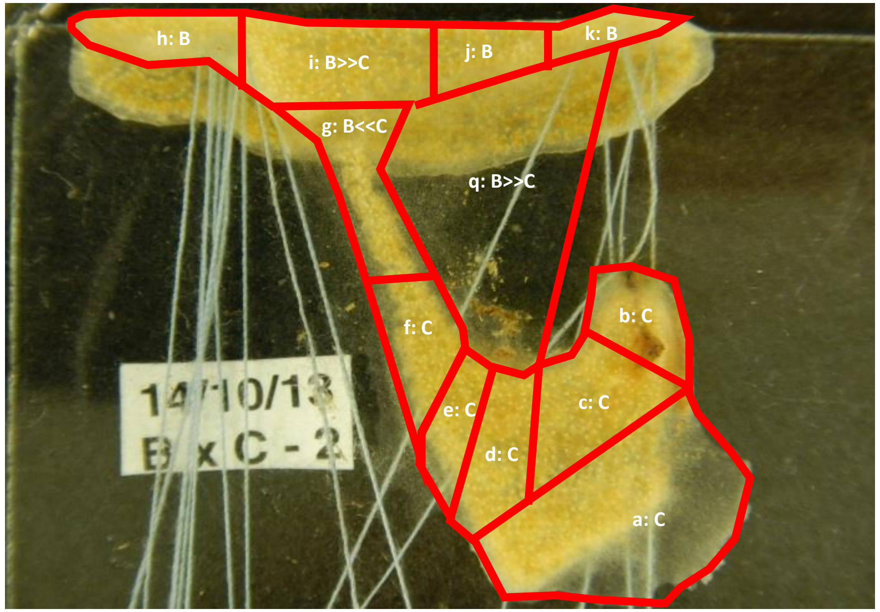

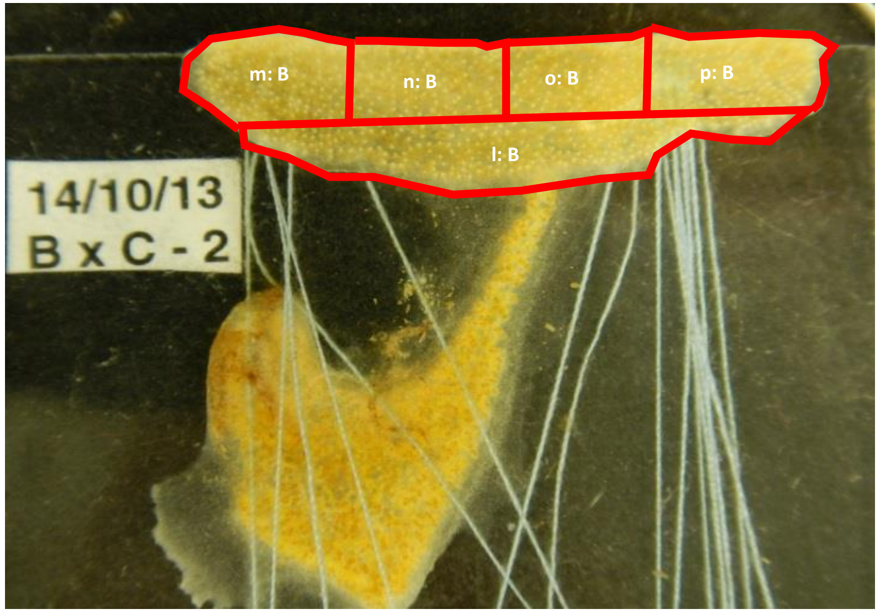

Pairing B x D – 1

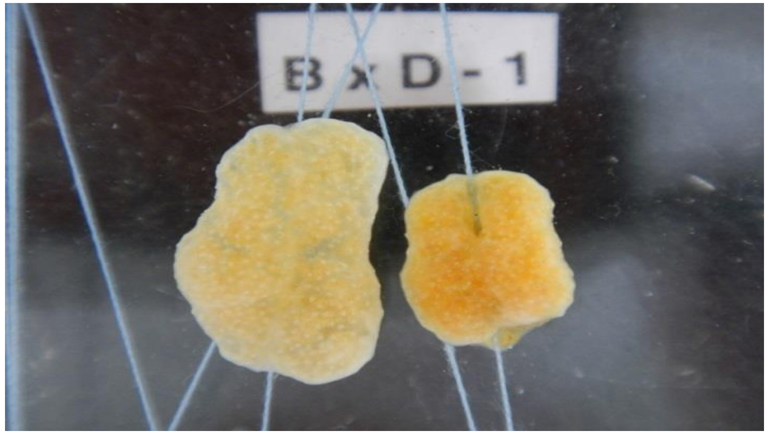

BxD-1: 02-10-2013

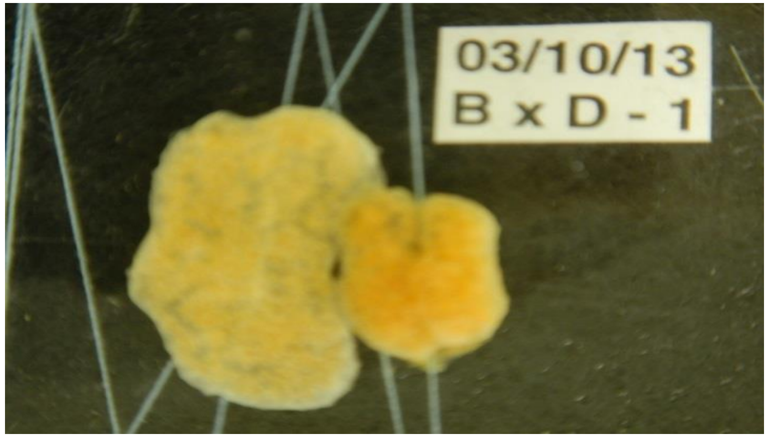

BxD-1: 03-10-2013

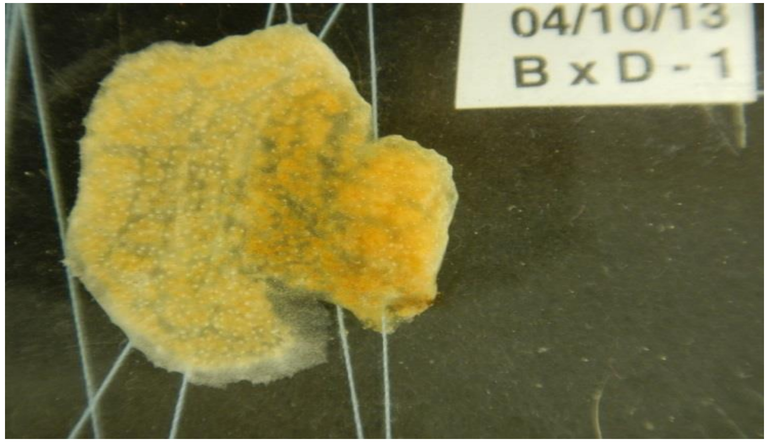

BxD-1: 04-10-2013

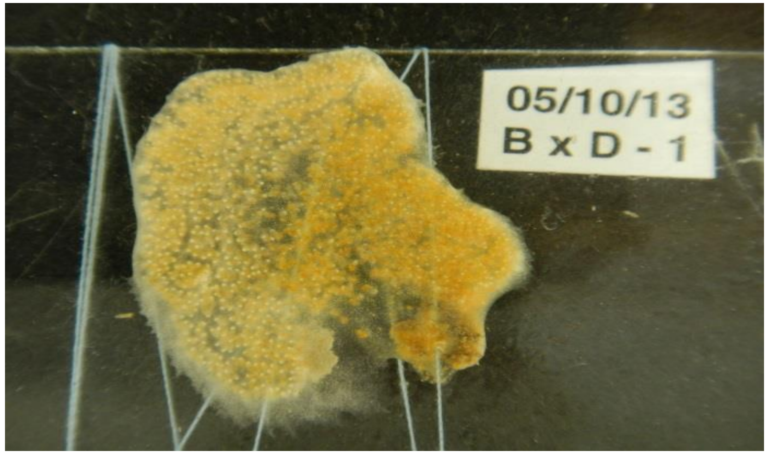

BxD-1: 05-10-2013

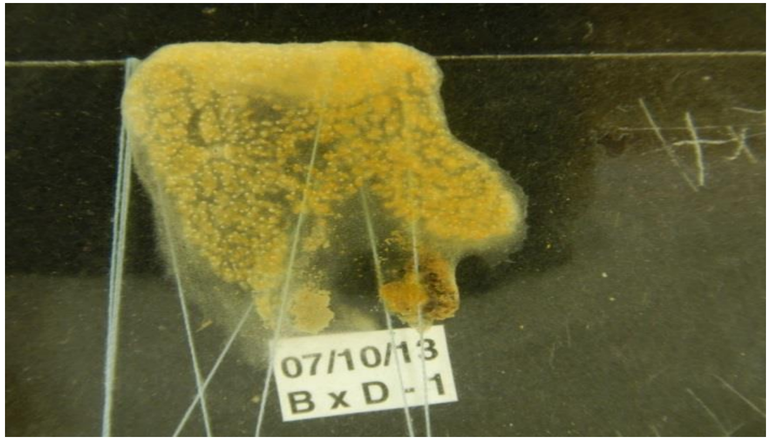

BxD-1: 07-10-2013

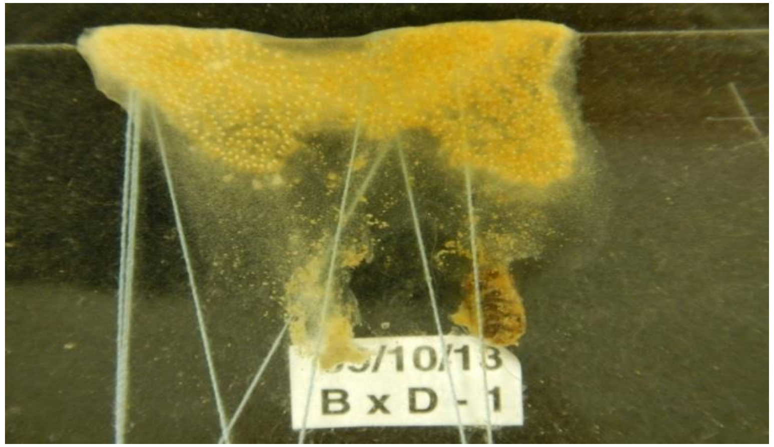

BxD-1: 09-10-2013

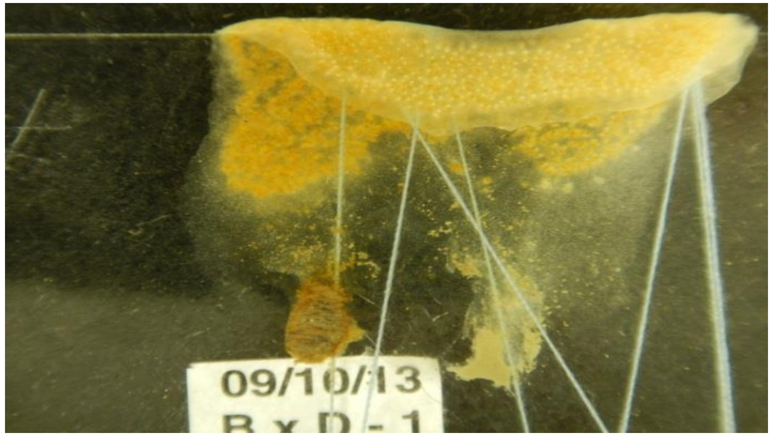

BxD-1: 09-10-2013 (reverse)

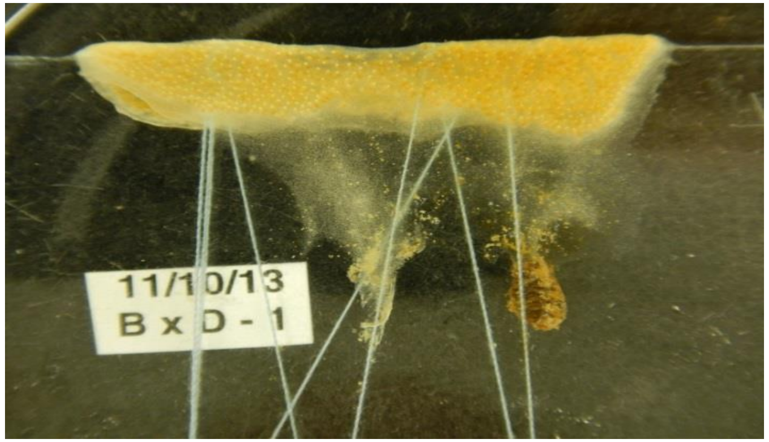

BxD-1: 11-10-2013

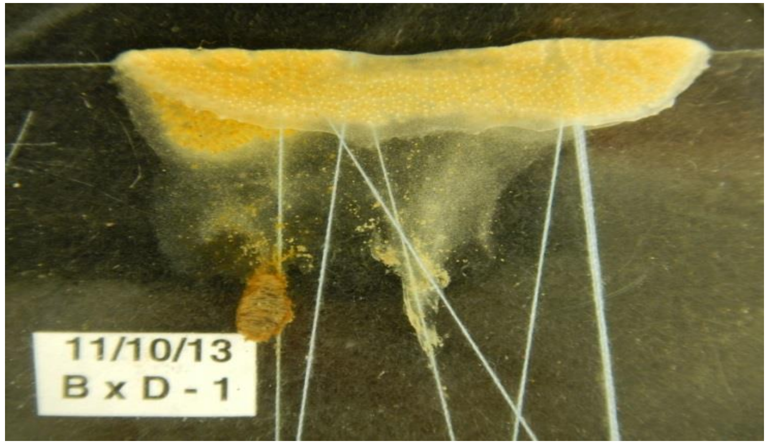

BxD-1: 11-10-2013 (reverse)

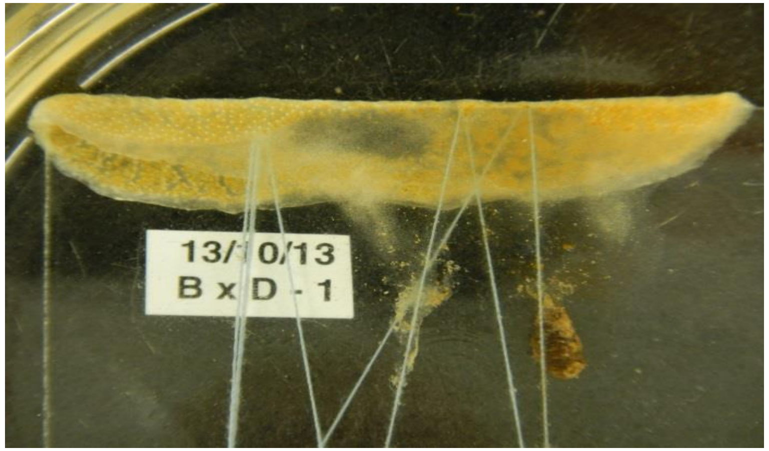

BxD-1: 13-10-2013

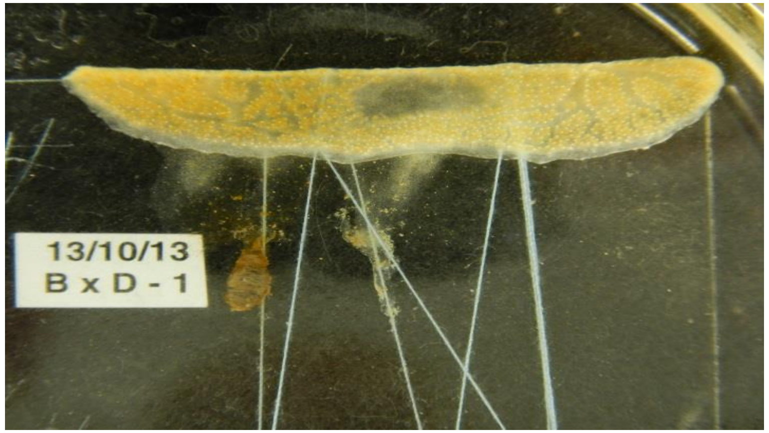

BxD-1: 13-10-2013 (reverse)

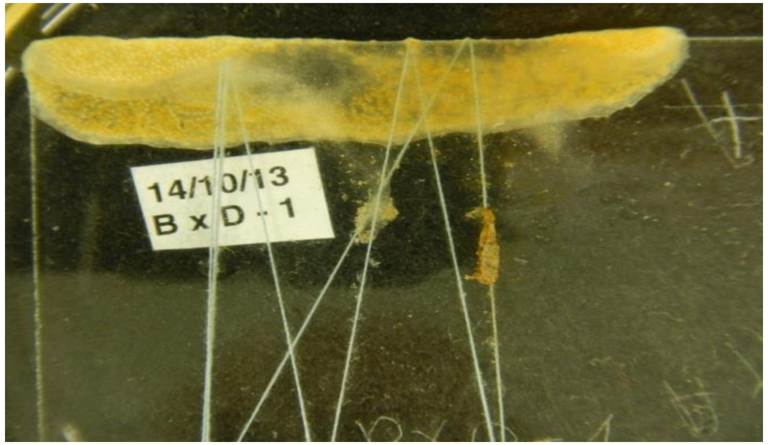

BxD-1: 14-10-2013

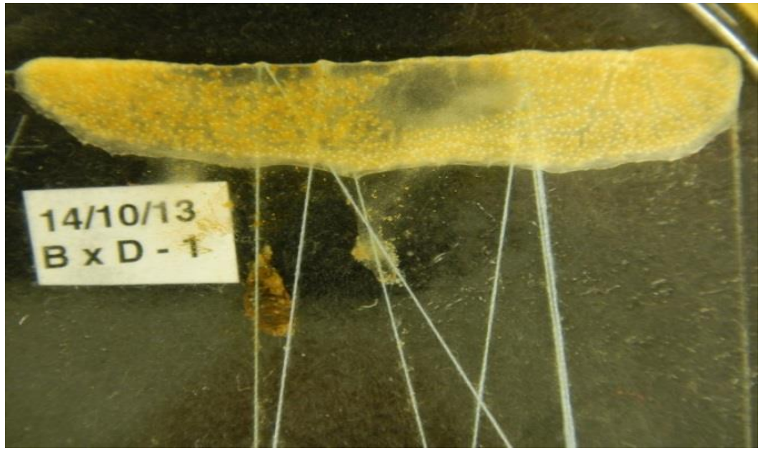

BxD-1: 14-10-2013 (reverse)

BxD–1: 12 day pairing genotyping schematic

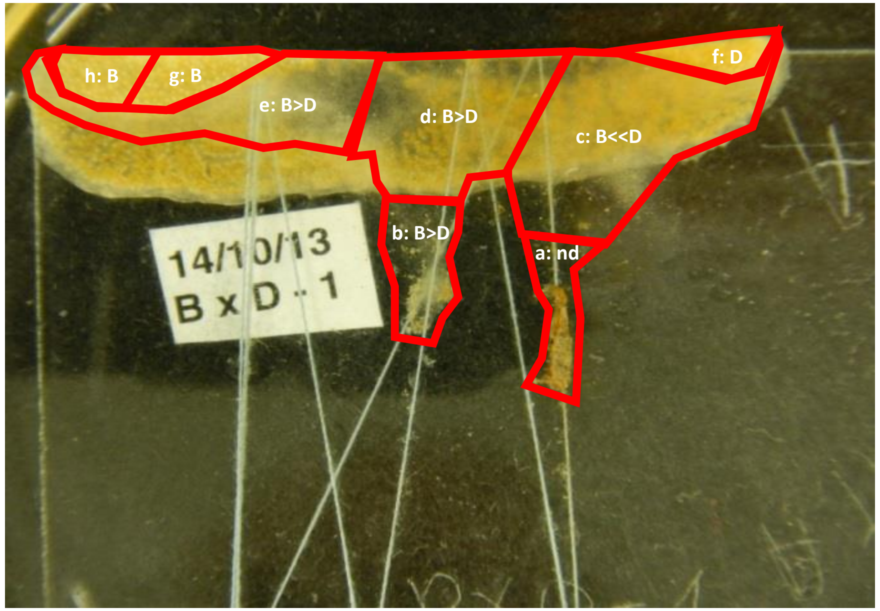

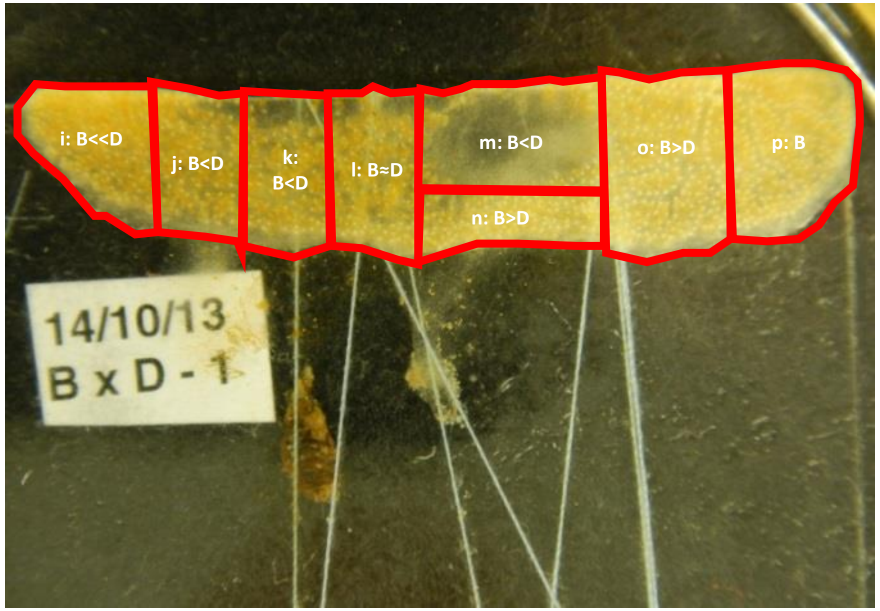

Pairing B x D – 2

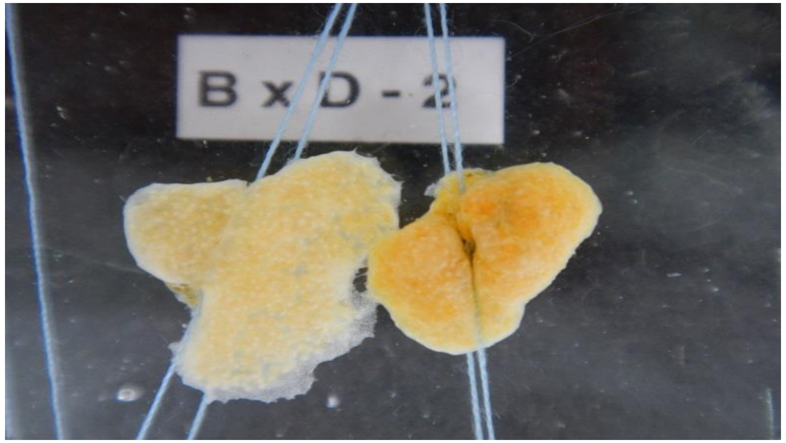

BxD-2: 02-10-2013

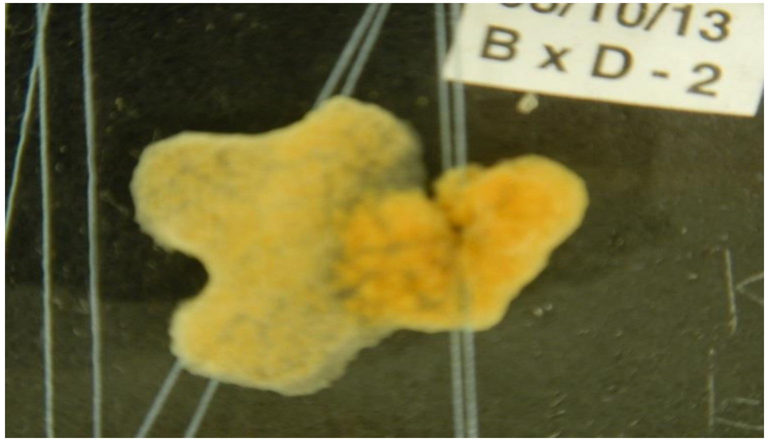

BxD-2: 03-10-2013

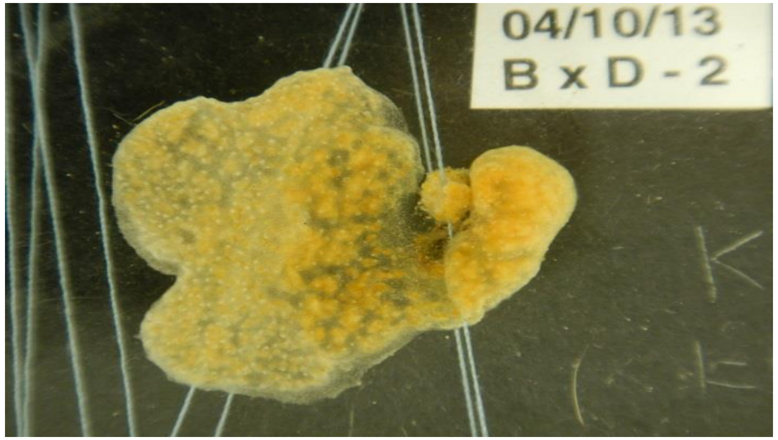

BxD-2: 04-10-2013

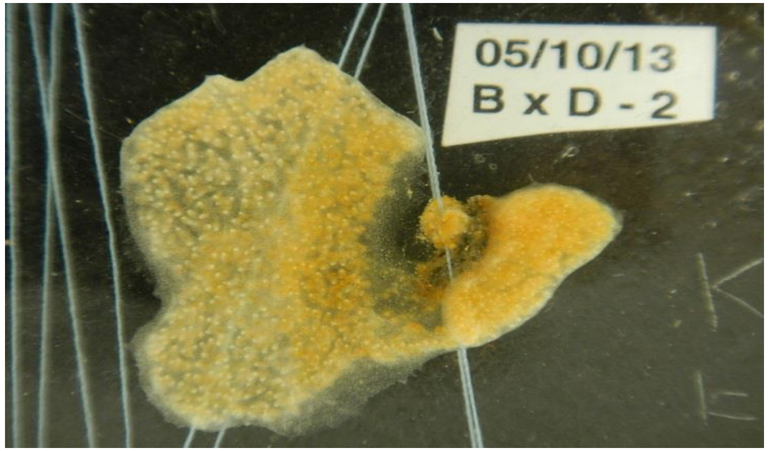

BxD-2: 05-10-2013

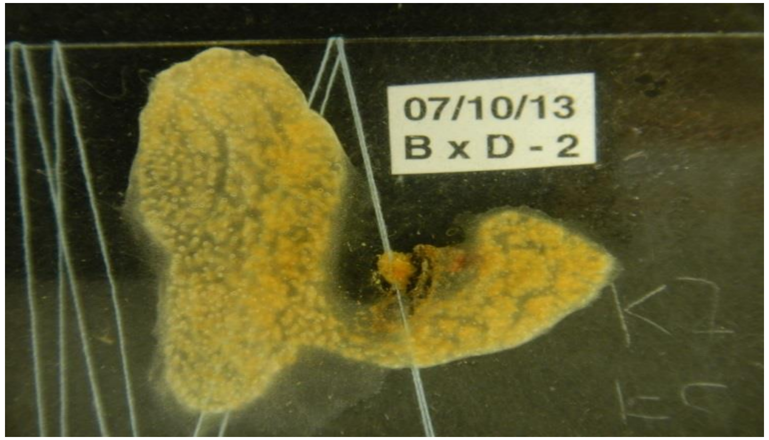

BxD-2: 07-10-2013

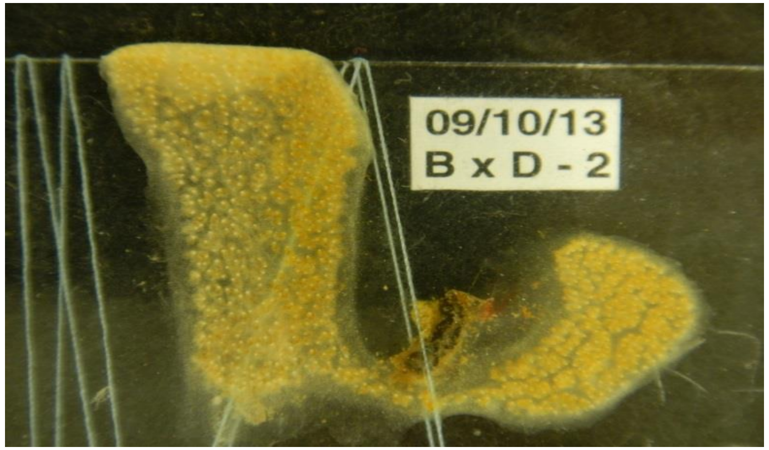

BxD-2: 09-10-2013

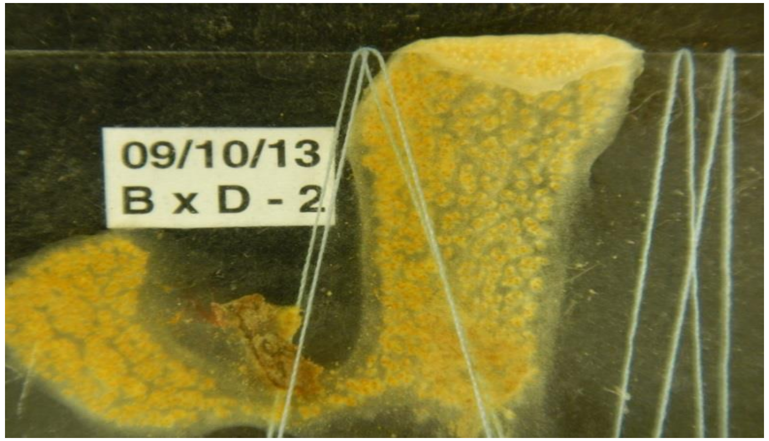

BxD-2: 09-10-2013 (reverse)

BxD-2: 7 day pairing genotyping schematic

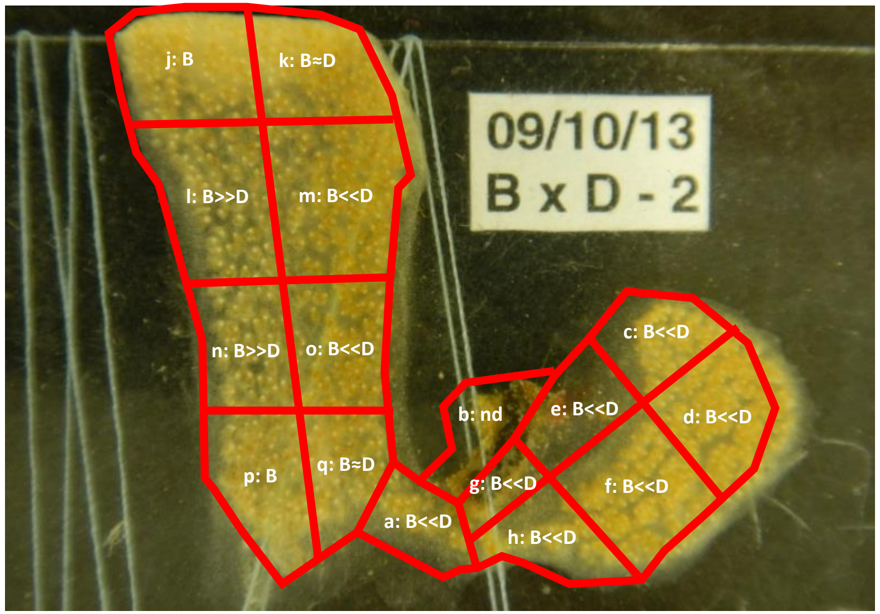

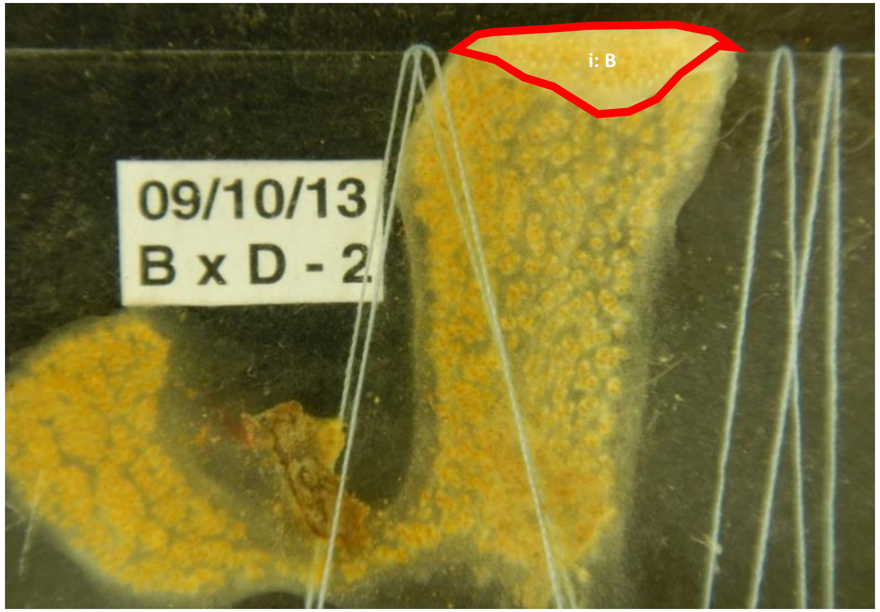

Pairing C x D – 1

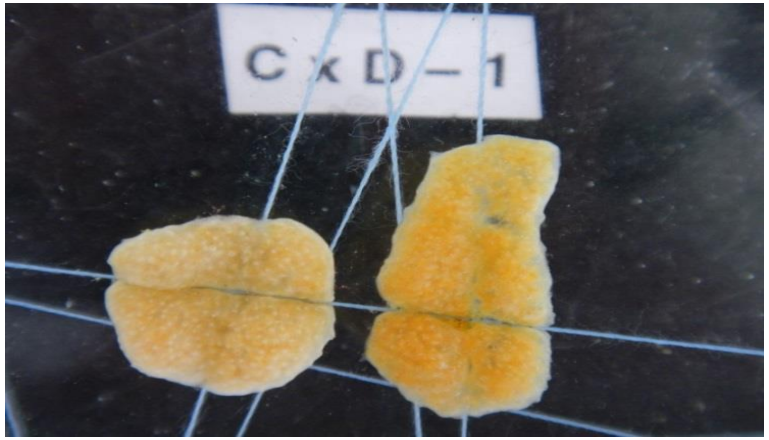

CxD-1: 02-10-2013

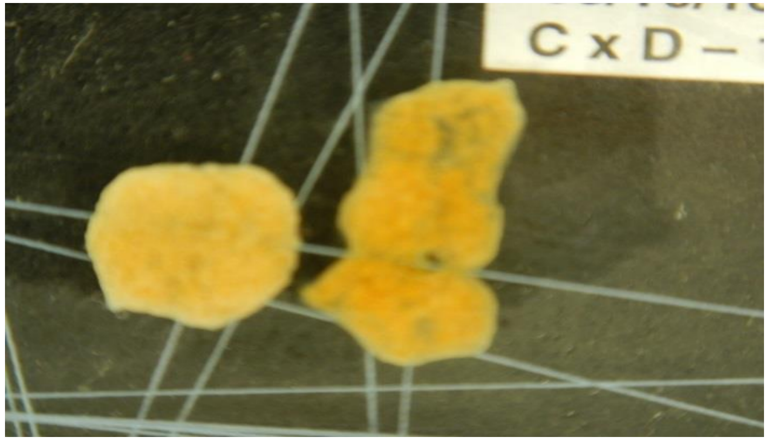

CxD-1: 03-10-2013

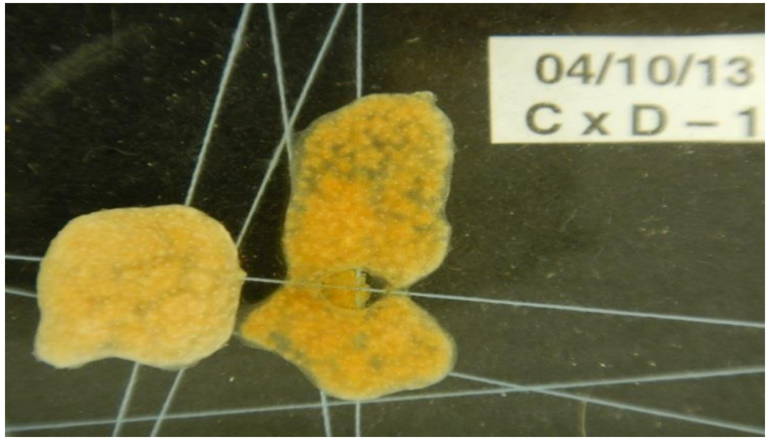

CxD-1: 04-10-2013

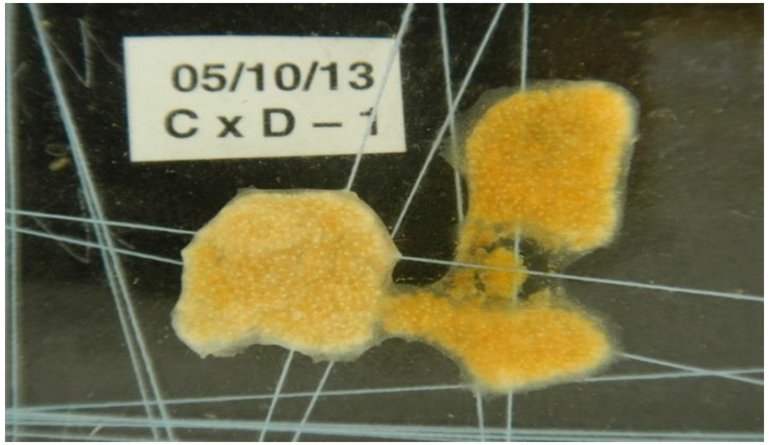

CxD–1: 05-10-2013

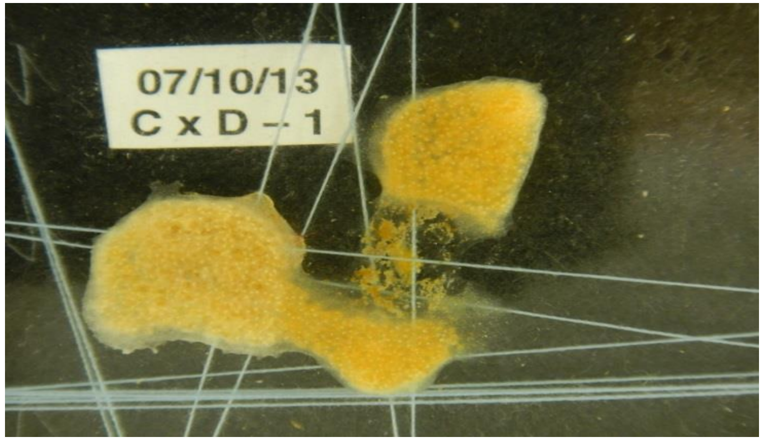

CxD-1: 07-10-2013

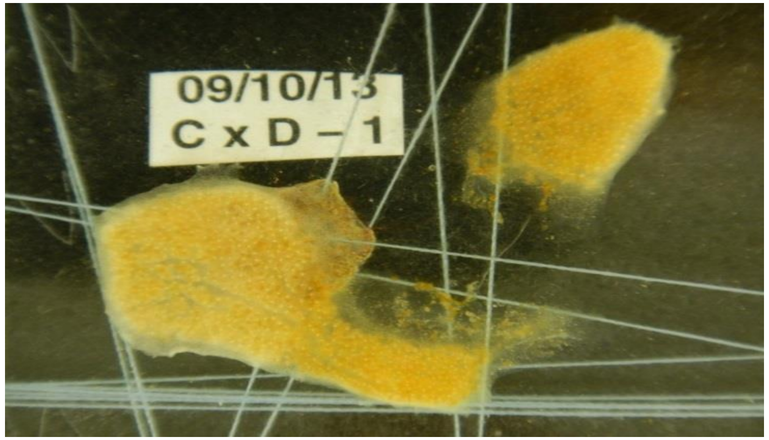

CxD-1: 09-10-2013

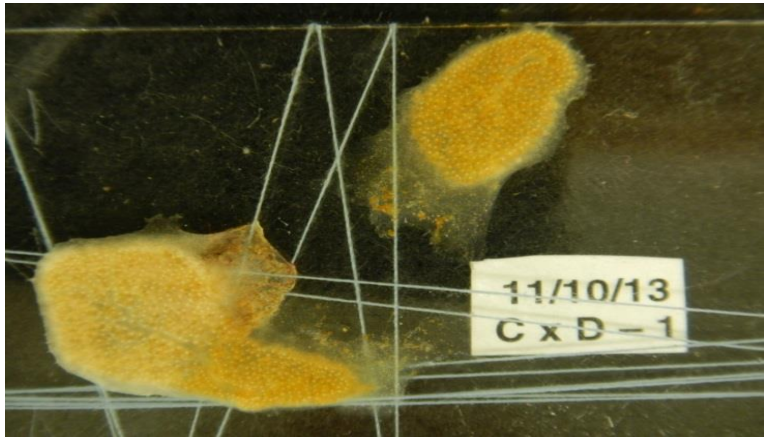

CxD-1: 11-10-2013

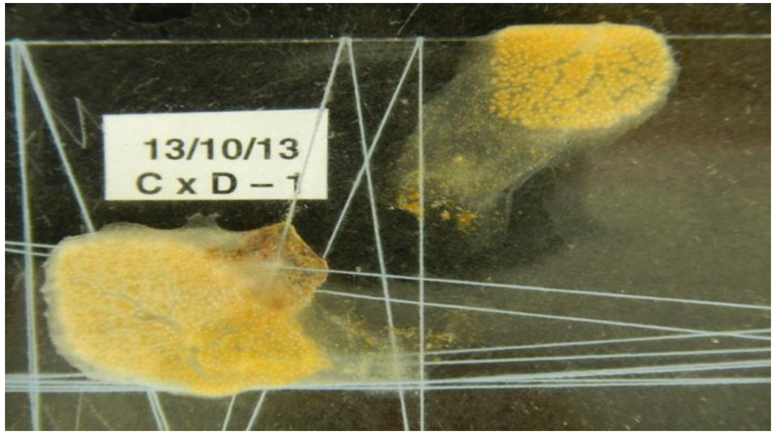

CxD-1: 13-10-2013

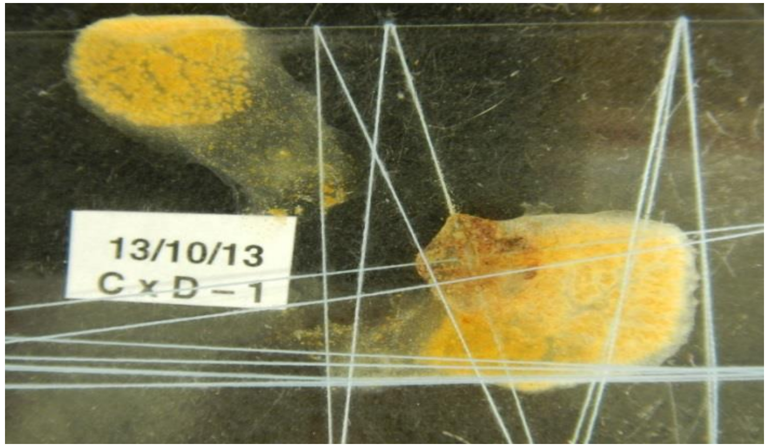

CxD-1: 13-10-2013 (reverse)

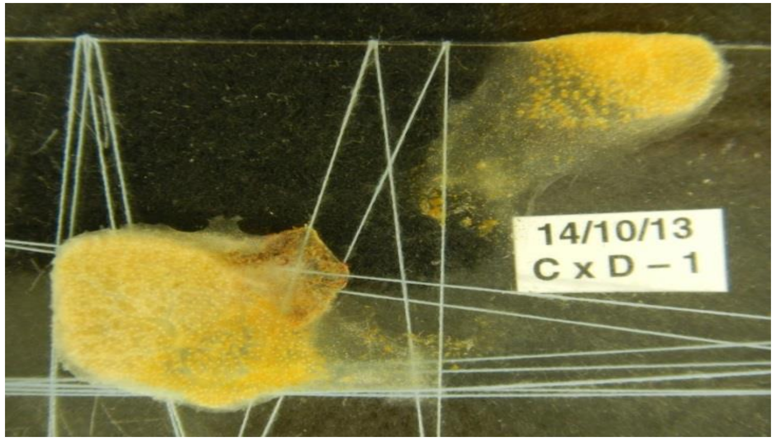

CxD-1: 14-10-2013

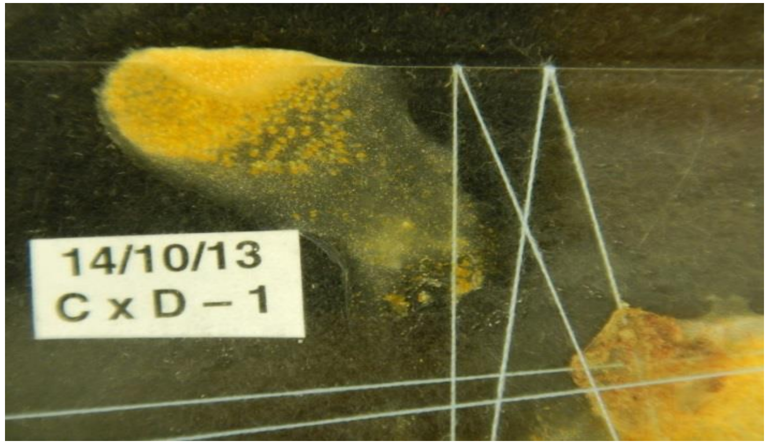

CxD-1: 14-10-2013 (reverse)

CxD-1: 12 day pairing: genotyping schematic

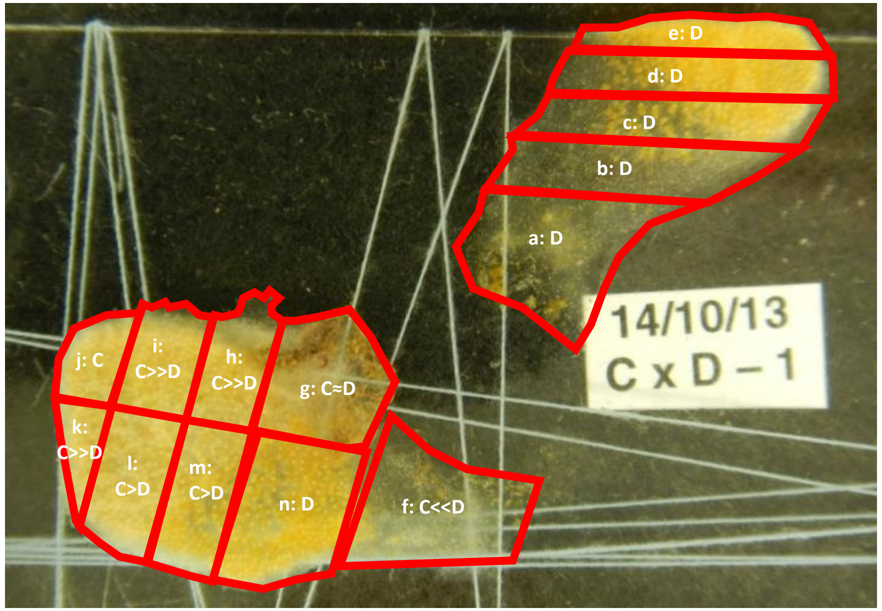

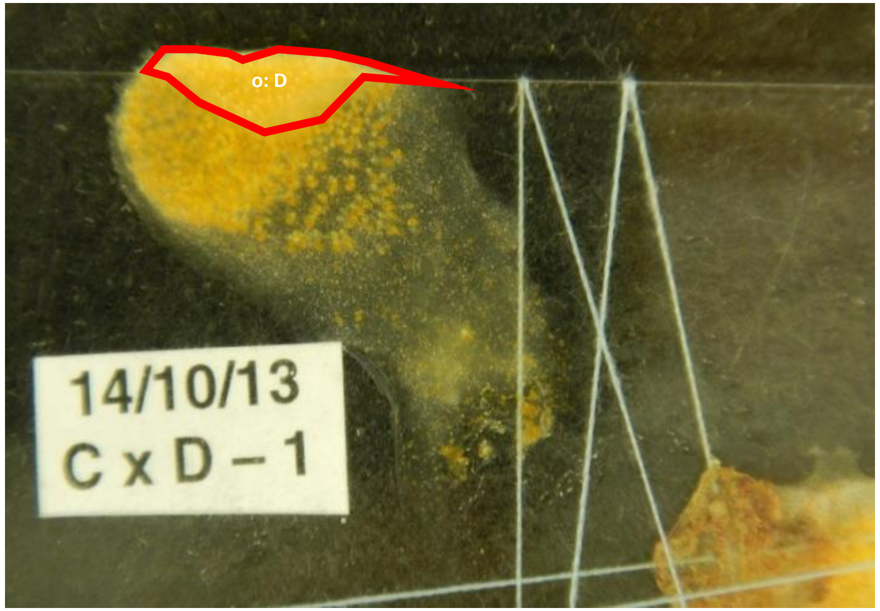

Pairing C x D – 2

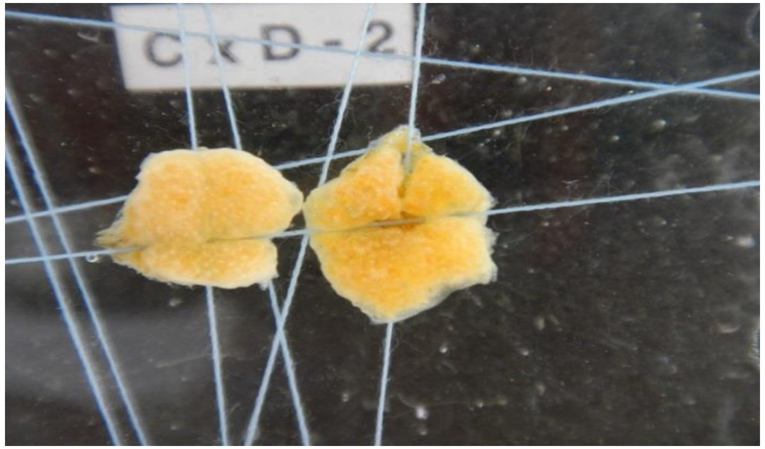

CxD-2: 02-10-2013

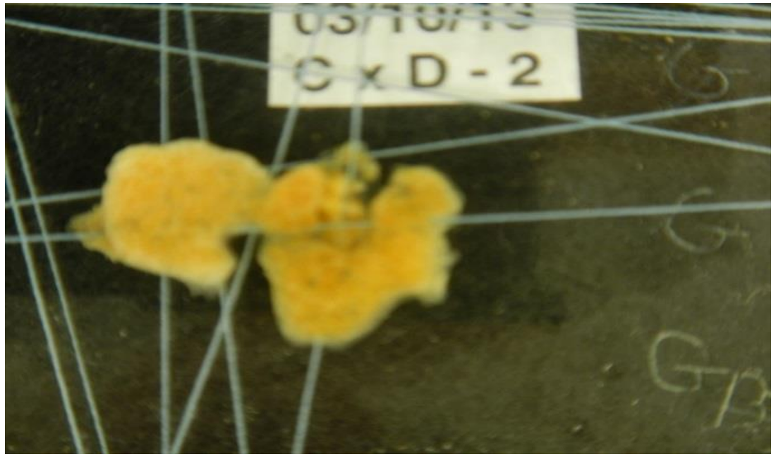

CxD-2: 03-10-2013

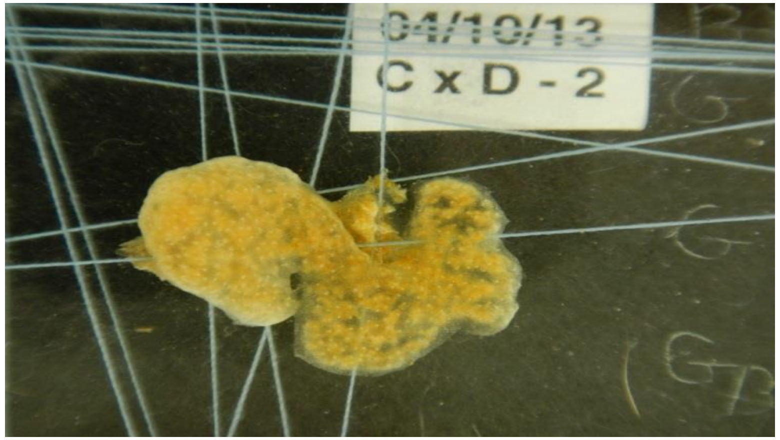

CxD-2: 04-10-2013

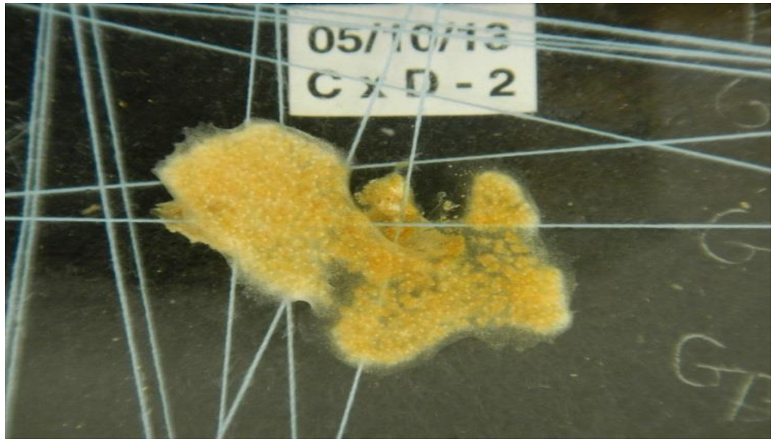

CxD-2: 05-10-2013

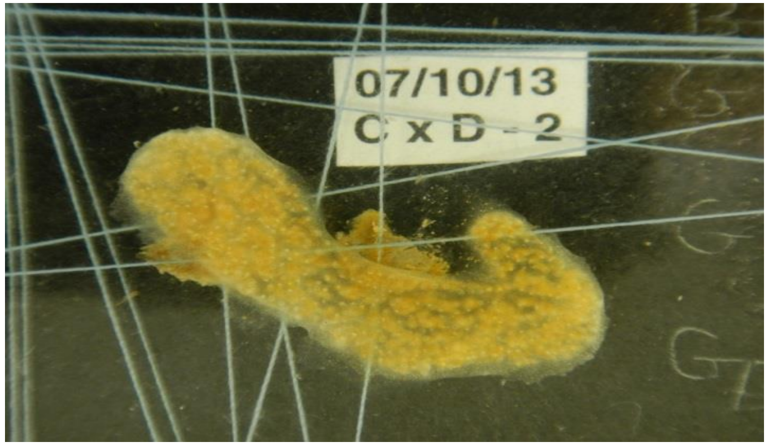

CxD-2: 07-10-2013

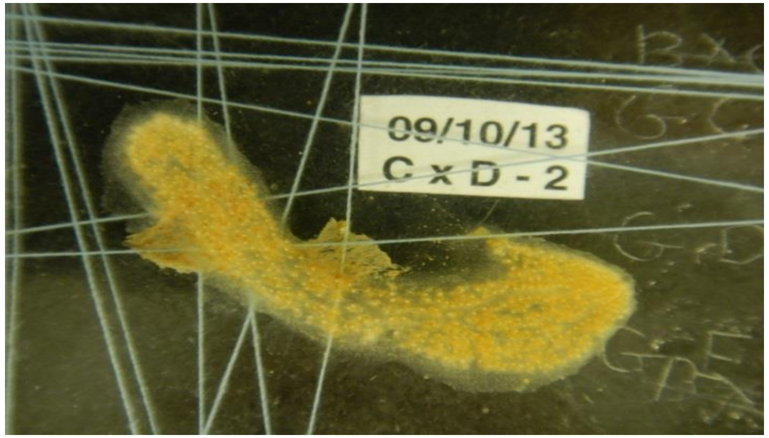

CxD-2: 09-10-2013

CxD-2: 7 day pairing genotyping schematic

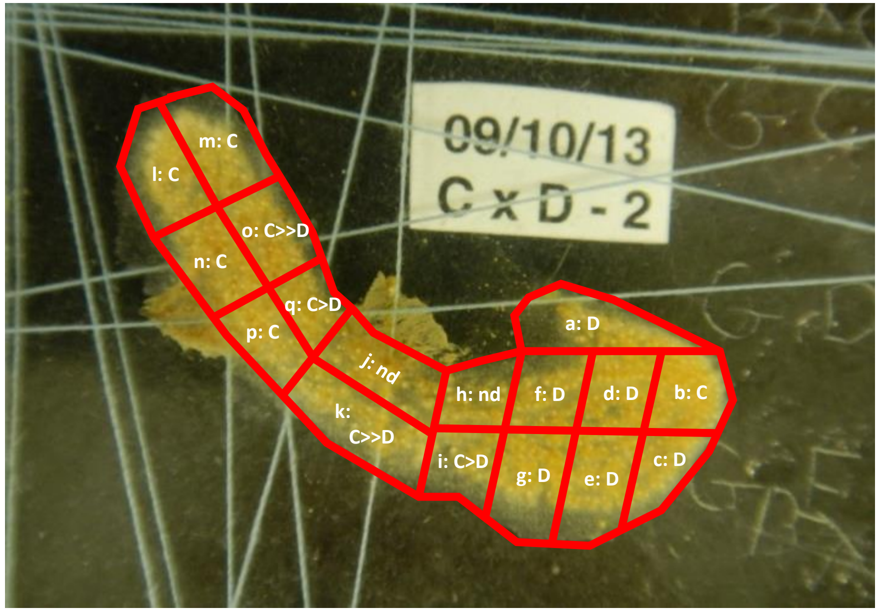

Supplement: Figure S1 — Pairings were established between ramets from four different D. vexillum colonies, denoted A–D, in duplicate. Photographic images of the 12 pairwise combinations were taken daily revealing the outcomes of the 6 possible pairwise combinations between the four genotypes. Descriptive summaries of the outcomes of the pairing experiments are given in Table 1. At the end of each pairing the D. vexillum colonies were dissected into the segments (denoted by lower-case letters: a, b, c, etc.), with the boundaries indicated by red lines. The dissected sections were genotyped at polymorphic microsatellite loci to determine the relative amounts of the paired ramet genotypes to each dissected region (for the semi-quantitative ratio assessment methodology see materials and methods). The cotton threads that held the D. vexillum subclones to the slides were not removed until termination of the experiment and so provide landmarks for the positioning of the ramets. Abbreviations: reverse, side of glass slide opposite to that on which the pairing was established; nd, not done. [file peerj-06-5006-s001.pdf]
