# Supplementary figures and images for: Efficient dispersal and substrate acquisition traits in a marine invasive species via transient chimerism and colony mobility
Source: PeerJ. 2018 Jun 13;6:e5006. doi: 10.7717/peerj.5006 (PMC6004106; doi:10.7717/peerj.5006)

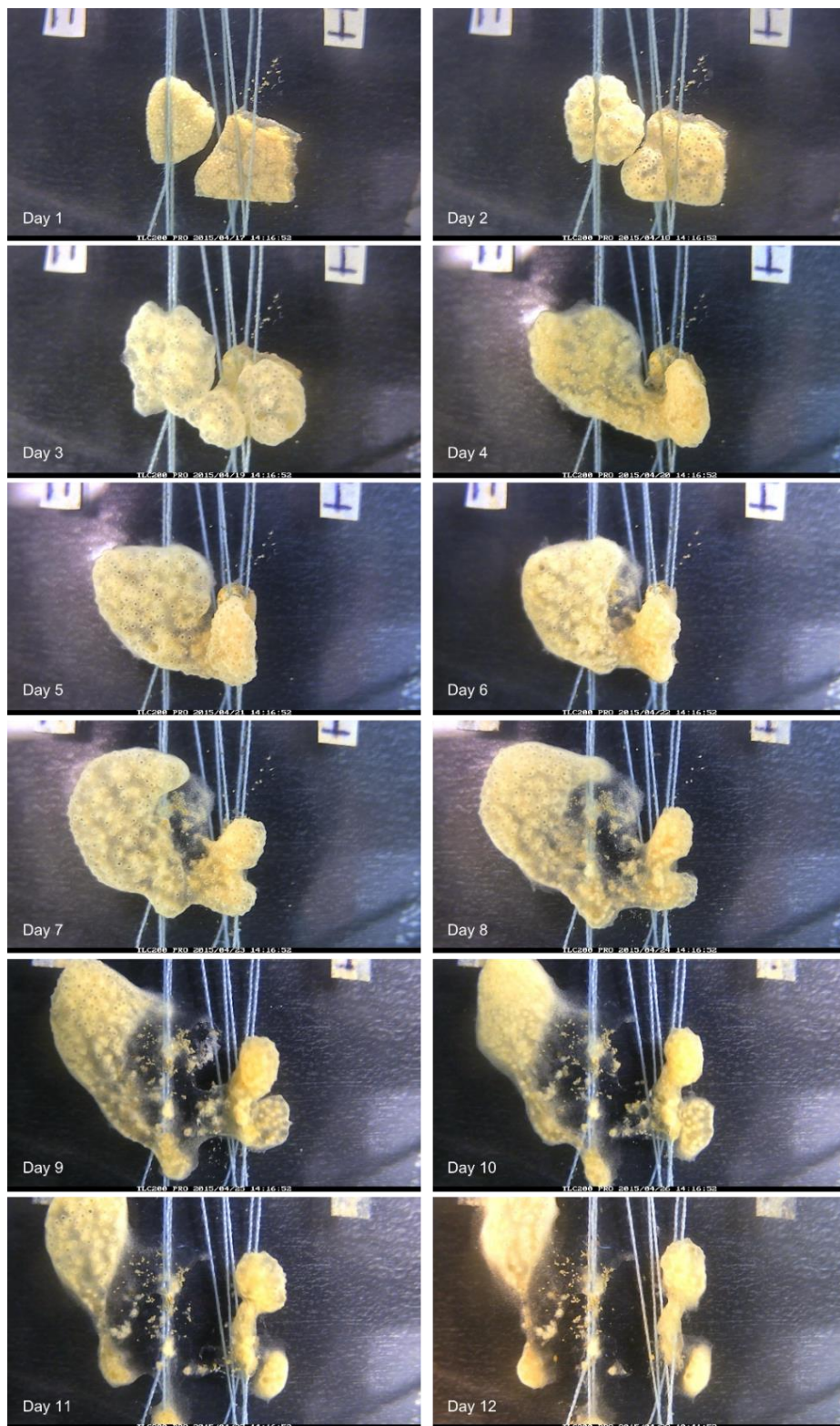

Fidler *et al.* Fig. S2

Supplement: Figure S2 — Representative images over 12 days and separated by 1 day, were extracted from the time-lapse film of the ramet H x I pairing (supplementary materials movie-S1). The images show an initial fusion of the Hand I ramets subsequently followed by separation movements. [file peerj-06-5006-s002.pdf]

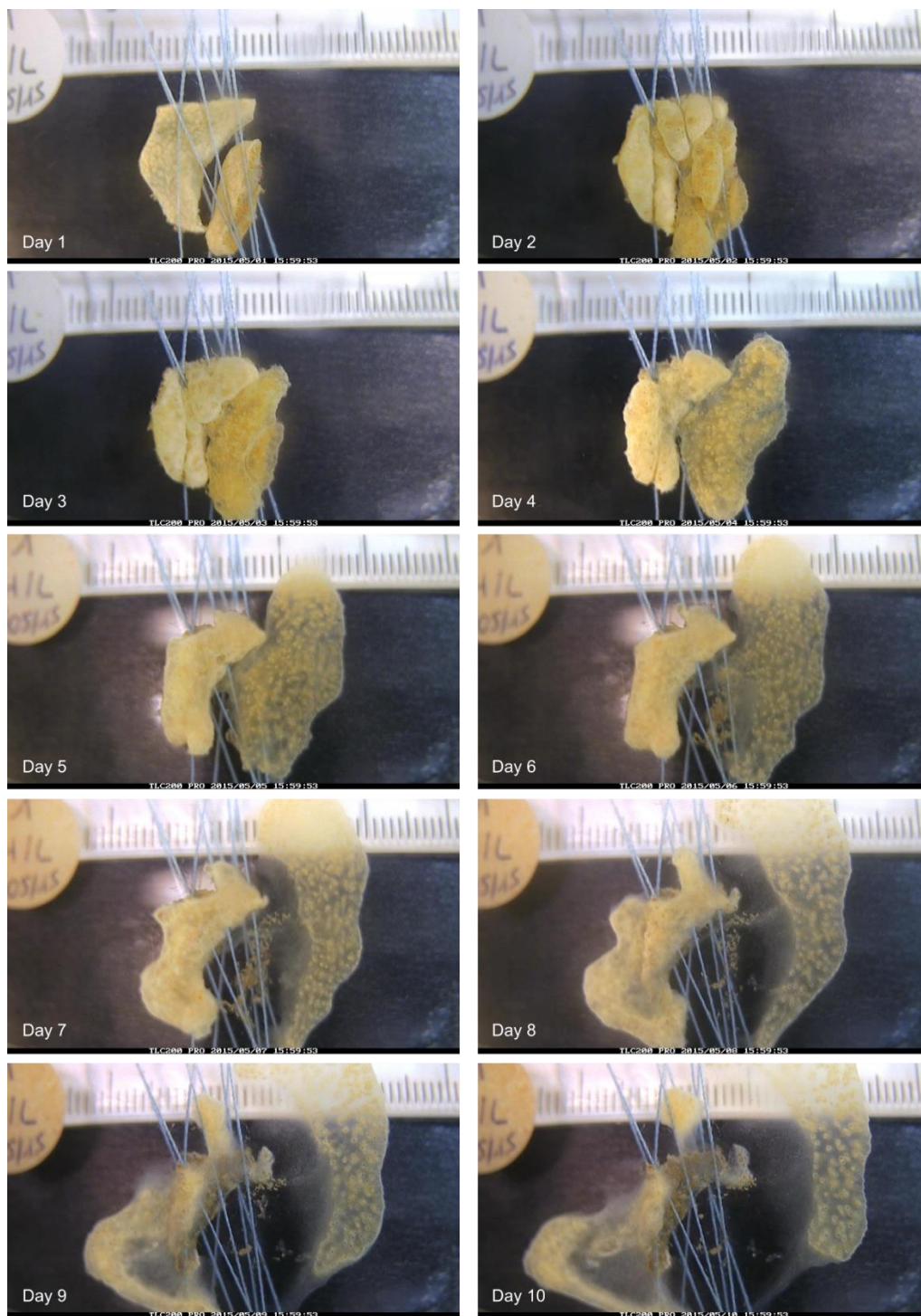

Fidler *et al.* Figure S3

Supplement: Figure S3 — Representative images over 10 days and separated by 1 day, were extracted from the time-lapse film of the ramet H x L pairing (supplementary materials movie-S2). The images show a lack of fusion of the H and L ramets that was followed by separation movements. [file peerj-06-5006-s003.pdf]
