# Supplementary material for: Efficient dispersal and substrate acquisition traits in a marine invasive species via transient chimerism and colony mobility
Source: PeerJ. 2018 Jun 13;6:e5006. doi: 10.7717/peerj.5006 (PMC6004106; doi:10.7717/peerj.5006)
Supplement: Supplemental Information 1 [file peerj-06-5006-s004.pdf]

Table S1. Details of the polymorphic microsatellite loci used to genotype *D. vexillum* ramets in this study .*Abbreviations*: ng, not genotyped; bp, base pairs.

Locus	Reference / GenBank acc. no.	Repeat	Primer orientation	PCR primer sequences (5' - 3')	Dye for generic M13 primer	Alleles of the four genotyped colonies (bp)
DVEX18	KU167099	(TGA)	Forward	TGTAAAACGACGGCCAGTTGTTTCAGGCGAGTTCATCG	6-FAM™	A (226/232)
						B (226/232)
						C (226)
			Reverse	CAATGAACAAACGCAACCGC		D (232)
						H (226 / 232)
						I (228)
DVEX19	KU167100	(CACG)	Forward	TGTAAAACGACGGCCAGTCTGGTCCAAATAACGAACGATTG	NED™	A (241)
						B (241)
						C (241 / 245)
			Reverse	TTTACGAGCAGCCAACGAAC		D (245)
						H (253)
						I (253 / 261)
DVEX23	Abbott *et al.* (2010) HM921432	(TTTG)	Forward	TGTAAAACGACGGCCAGTGTTGAAGTAGTGGCGGACAA	VIC®	A (217 / 221)
						B (225 / 230)
			Reverse	TGAAGACCATCAACGAGAGC		C (217 / 221)
						D (217 / 230)
DVEX32	Abbott *et al.* (2010) HM921437	(AAC)	Forward	TGTAAAACGACGGCCAGTCTGCTGCTCACTGAATCACA	PET™	A (253)
						B (251 / 259)
			Reverse	CGATGACTTTTCTCCCCATT		C (251 / 259)
						D, ng
DVEX05	Abbott *et al.* (2010) HM921428	(AC)	Forward	TGTAAAACGACGGCCAGTGCAAAACAGTTCGTGCATTT	NED™	A (160)
						B, ng
			Reverse	CCATCGGCTTTATGACATTG		C (160 / 162)
						D (156 / 160)

Table S2. Summary of the microsatellite loci used to discriminate *D. vexillum* zooid genotypes in the fusion experiments. Letters A, B, C, D, H and I refer to the different ramets used while details of the microsatellite loci are given in Table S1. Abbreviations: *np*, not performed; *iso*, isogeneic combination.

	**A**	**B**	**C**	**D**	**H**
**A**	iso	-	-	-	-
**B**	DVEX23<br>DVEX32	iso	-	-	-
**C**	DVEX18<br>DVEX05<br>DVEX32	DVEX18<br>DVEX19<br>DVEX23	iso	-	-
**D**	DVEX19<br>DVEX23	DVEX19<br>DVEX23	DVEX05<br>DVEX23	iso	-
**H**	np	np	np	np	iso
**I**	np	np	np	np	DVEX18<br>DVEX19
