# Supplementary material for: Efficient dispersal and substrate acquisition traits in a marine invasive species via transient chimerism and colony mobility
Source: PeerJ. 2018 Jun 13;6:e5006. doi: 10.7717/peerj.5006 (PMC6004106; doi:10.7717/peerj.5006)
Supplement: Supplemental Information 2 — Sequences corresponding to GenBank entries KU167099 and KU167100. [file peerj-06-5006-s007.pdf]

Dear GenBank Submitter:

Thank you for your submission.

Based on the data submitted to us, the scheduled release date for your submission is:

Nov 19, 2019

However, if the accession number is published prior to that date, the sequence will be released upon publication.

Note that the entire sequence will be released when the article citing this accession number is published or on the above release date, whichever comes first. If this is not what you intended, please notify us immediately so that we can discuss your submission in more detail.

If this date is not correct, please let us know as soon as possible, otherwise this submission will be released on the date indicated above. The data will become available from our different servers within a few days of release. The data are simultaneously made available to EMBL in Europe and the DNA Data Bank of Japan.

Minor changes may have been made to your original submission in order to conform to database annotation conventions. In particular, please review:

- Spelling
- Citation data (page span, etc.)
- Nomenclature ('official' gene names, product labels, etc.)
- Taxonomic and source data
- Feature spans and descriptions (particularly non-coding regions)

Additional edits may have been made to your submission, including:

- Exon spans adjusted to conform to the splice donor/acceptor consensus sequences, GT and AG, respectively
- Strings of N's at the ends of sequences and linker past the polyA tail removed
- Any mRNA or ribosomal RNA sequences submitted on the minus-strand have been reverse-complemented

If your submission contained unpublished organism names, the scientific names have been changed to temporary names. Please notify us when the organism names are published and we will update them accordingly.

Please send any revisions, including bibliographic information (e.g., conversion from unpublished to published), biological data (e.g., new features), or sequence data to:

gb-admin@ncbi.nlm.nih.gov

Since the flatfile record is a display format only and is not an editable format of the data, do not make changes directly to a flatfile. For complete information about different methods to update a sequence record, see: http://www.ncbi.nlm.nih.gov/Genbank/update.html

An accession number has been assigned to each nucleotide sequence and was previously provided to you. Note that during the processing, we have assigned protein identifiers to any proteins within the submission. This is fielded as /protein_id.

We strongly recommend that these numbers appear in any publication
which reports or discusses these data, so that readers may easily
retrieve your data from our databases.

Thank you once again for your submission.

Please reply using the original subject line.
This will allow for faster processing of your correspondence.

Sincerely,

DeAnne Olsen Cravaritis, Ph.D.
GenBank Direct Submission Staff
gb-admin@ncbi.nlm.nih.gov

GenBank flat file:

```
LOCUS       KU167099                 495 bp    DNA     linear   INV 18-NOV-2015
DEFINITION  Didemnum vexillum microsatellite DVEX18 sequence.
ACCESSION   KU167099
VERSION     KU167099
KEYWORDS    .
SOURCE      Didemnum vexillum
  ORGANISM  Didemnum vexillum
            Eukaryota; Metazoa; Chordata; Tunicata; Ascidiacea; Enterogona;
            Aplousobranchia; Didemnidae; Didemnum.
REFERENCE   1  (bases 1 to 495)
  AUTHORS   Bacq-Labreuil,A., Rinkevich,B. and Fidler,A.E.
  TITLE     Direct Submission
  JOURNAL   Submitted (18-NOV-2015) Institute of Marine Science, University of
            Auckland, Private Bag 92019, Auckland 1142, New Zealand
COMMENT     ##Assembly-Data-START##
            Sequencing Technology :: Sanger dideoxy sequencing
            ##Assembly-Data-END##
FEATURES             Location/Qualifiers
     source          1..495
                     /organism="Didemnum vexillum"
                     /mol_type="genomic DNA"
                     /db_xref="taxon:516032"
                     /PCR_primers="fwd_name: fwd_primer1, fwd_seq:
                     tgtttcaggcgagttcatcg, rev_name: rev_primer1, rev_seq:
                     caatgaacaaacgcaaccgc"
     repeat_region   251..271
                     /rpt_type=tandem
                     /satellite="microsatellite:DVEX18"
ORIGIN
        1 attcattaat cttaacataa gtaataggtc gtagtttttt tattttaaat aaatcagcct
       61 tttttatatt caatttttttt gaaaaattaa gaattcgaaa gaaactgatg acgtgatgtg
      121 actagccatg aaattaattt ttcaaaatag aattgtttgt gaagtaggct gattgatttc
      181 aaaaaactaa gaccctttgt caaacaatct gcaatataat ttgatcgatg tttcaggcga
      241 gttcatcgag tgatgatgat gatgatgatg atgtcggtga tgttgaagtt gaaacgtttc
      301 atcgaagtgt ttcattaaaa agtgatcaca atacgttgtc tgttgaaaga aatcaaccag
      361 gtaagttaca gagcgaggtt ttctatcgta gtttcgagat attttaagaa acttgactgt
      421 tgcgagcggt tgcgtttgtt cattgagaaa cccaatgctg ctgaggcatt cttgcagagg
      481 actgttggaa atatg
//
LOCUS       KU167100                 457 bp    DNA     linear   INV 18-NOV-2015
DEFINITION  Didemnum vexillum microsatellite DVEX19 sequence.
ACCESSION   KU167100
VERSION     KU167100
KEYWORDS    .
SOURCE      Didemnum vexillum
  ORGANISM  Didemnum vexillum
            Eukaryota; Metazoa; Chordata; Tunicata; Ascidiacea; Enterogona;
            Aplousobranchia; Didemnidae; Didemnum.
```

```
REFERENCE   1  (bases 1 to 457)
  AUTHORS   Bacq-Labreuil,A., Rinkevich,B. and Fidler,A.E.
  TITLE     Direct Submission
  JOURNAL   Submitted (18-NOV-2015) Institute of Marine Science, University of
            Auckland, Private Bag 92019, Auckland 1142, New Zealand
COMMENT     ##Assembly-Data-START##
            Sequencing Technology :: Sanger dideoxy sequencing
            ##Assembly-Data-END##
FEATURES             Location/Qualifiers
     source          1..457
                     /organism="Didemnum vexillum"
                     /mol_type="genomic DNA"
                     /db_xref="taxon:516032"
                     /PCR_primers="fwd_name: fwd_primer2, fwd_seq:
                     ctggtccaaataacgaacgattg, rev_name: rev_primer2, rev_seq:
                     tttacgagcagccaacgaac"
     repeat_region   231..258
                     /rpt_type=tandem
                     /satellite="microsatellite:DVEX19"
ORIGIN
        1 atatttttg aaaatgtaat aaaccgaatt tacaactcta gatttcttcc tgaacaacca
       61 cattatcaat tgtacagtga aatctttaaa ccataatttc ctgtaaaaaa caagtgtgag
      121 gatacttaat tgtaaactga agcaggtagt gaaacttatt tatctggtcc aaataacgaa
      181 cgattgattt tttaactttt taaggtaaaa aattaaaaca cacacgcaca cacgcacgca
      241 cgcacgcacg cacgcacgca aacaagcaag cacaaacaca cgcacgcaca cgtttacttt
      301 aagttttaag aacagccaac ctcaacacga aatttatttc ttttcatatt taattttctt
      361 tataagaacc aataaatttg gtgttcgttg gctgctcgta aattttttag aatttttcgg
      421 ctttcatttg ttaatatgtt gtttgaaatt tttaaca
```
